# Supplementary material for: Alignment-Integrated Reconstruction of Ancestral Sequences Improves Accuracy
Source: Genome Biol Evol. 2020 Aug 12;12(9):1549–65. doi: 10.1093/gbe/evaa164 (PMC7523730; doi:10.1093/gbe/evaa164)
Supplement: evaa164_Supplementary_Data [file evaa164_supplementary_data.pdf]

Supplementary Information

Supplementary Tables

domain	tree depth	max distance to root	branch lengths		
			median	mean (stdev)	maximum
CARD	39	5.11	0.0141	0.067 (0.179)	3.19
DSRM1	43	4.41	0.0173	0.099 (0.227)	2.63
DSRM2	44	4.41	0.0156	0.092 (0.212)	2.37
DSRM3	43	3.50	0.0029	0.042 (0.152)	2.34
RD	35	3.33	0.0055	0.038 (0.101)	1.90

Table S1. Empirical protein domain family phylogenies used to simulate sequence data. We simulated protein sequence data along empirical phylogenies with the indicated properties. Tree depth indicates the number of nodes on the longest path from the root to any leaf node on the phylogeny. Max distance to root indicates the largest cumulative number of expected substitutions/site along any path from the root to any leaf node. Branch length summary statistics are also indicated.

domain	alignment	alignment_len	P(gap)	P(variable)	P(parsimony)
CARD	correct	530.5 (5.4513)	0.84 (0.0016)	0.32 (0.0031)	0.25 (0.0024)
1273 sequences	structaligned	283.8 (5.3329)	0.71 (0.0044)	0.88 (0.0075)	0.68 (0.0051)
	aligned_clustalw	123.6 (1.7333)	0.33 (0.0060)	0.98 (0.0014)	0.97 (0.0014)
	aligned_mafft	365.9 (10.0316)	0.77 (0.0076)	0.85 (0.0051)	0.67 (0.0107)
	aligned_msaprobs	270.5 (15.3096)	0.69 (0.0160)	0.92 (0.0041)	0.82 (0.0008)
	aligned_muscle	428.1 (14.1378)	0.81 (0.0079)	0.91 (0.0085)	0.77 (0.0143)
	aligned_probalign	353.6 (18.4272)	0.76 (0.0107)	0.93 (0.0069)	0.85 (0.0150)
	aligned_probcons	422.0 (14.5304)	0.80 (0.0063)	0.87 (0.0078)	0.64 (0.0231)
	aligned_tcoffee	394.9 (8.0380)	0.79 (0.0056)	0.80 (0.0155)	0.61 (0.0064)
DSRM1	correct	180.4 (4.8378)	0.62 (0.0089)	0.50 (0.0097)	0.45 (0.0104)
1376 sequences	structaligned	124.1 (2.7787)	0.45 (0.0117)	0.89 (0.0113)	0.77 (0.0166)
	aligned_clustalw	90.0 (1.3250)	0.25 (0.0105)	0.98 (0.0045)	0.95 (0.0082)
	aligned_mafft	270.3 (10.9990)	0.75 (0.0111)	0.89 (0.0150)	0.72 (0.0219)
	aligned_msaprobs	159.7 (6.4894)	0.57 (0.0163)	0.85 (0.0186)	0.70 (0.0189)
	aligned_muscle	204.5 (11.3463)	0.66 (0.0173)	0.92 (0.0138)	0.78 (0.0160)
	aligned_probalign	232.2 (13.5809)	0.70 (0.0183)	0.93 (0.0104)	0.85 (0.0218)
	aligned_probcons	160.5 (4.5662)	0.58 (0.0113)	0.79 (0.0200)	0.64 (0.0186)
	aligned_tcoffee	197.3 (7.9233)	0.65 (0.0127)	0.79 (0.0241)	0.64 (0.0215)
DSRM2	correct	263.1 (8.0518)	0.74 (0.0070)	0.42 (0.0061)	0.34 (0.0066)
1497 sequences	structaligned	156.2 (5.0789)	0.56 (0.0118)	0.89 (0.0114)	0.75 (0.0116)
	aligned_clustalw	97.8 (1.7876)	0.30 (0.0104)	0.99 (0.0048)	0.95 (0.0077)
	aligned_mafft	302.2 (6.4770)	0.77 (0.0043)	0.88 (0.0074)	0.72 (0.0150)
	aligned_msaprobs	187.0 (3.9861)	0.63 (0.0068)	0.85 (0.0169)	0.68 (0.0181)
	aligned_muscle	247.5 (13.4075)	0.72 (0.0157)	0.92 (0.0137)	0.78 (0.0155)
	aligned_probalign	265.5 (16.1983)	0.73 (0.0218)	0.93 (0.0137)	0.83 (0.0256)
	aligned_probcons	197.1 (7.8407)	0.65 (0.0125)	0.82 (0.0114)	0.64 (0.0219)
	aligned_tcoffee	253.8 (14.2773)	0.72 (0.0156)	0.79 (0.0178)	0.65 (0.0147)
DSRM3	correct	86.2 (1.8123)	0.19 (0.0172)	0.84 (0.0143)	0.82 (0.0170)
718 sequences	structaligned	84.9 (1.7729)	0.18 (0.0169)	0.91 (0.0120)	0.86 (0.0124)
	aligned_clustalw	77.3 (0.9667)	0.10 (0.0099)	0.98 (0.0050)	0.95 (0.0076)
	aligned_mafft	109.5 (6.8219)	0.35 (0.0408)	0.86 (0.0215)	0.72 (0.0352)
	aligned_msaprobs	86.2 (2.2988)	0.19 (0.0212)	0.89 (0.0184)	0.84 (0.0177)
	aligned_muscle	85.9 (1.5878)	0.19 (0.0158)	0.93 (0.0114)	0.86 (0.0137)

	aligned_probalign	125.2 (5.9643)	0.44 (0.0279)	0.87 (0.0145)	0.69 (0.0265)
	aligned_probcons	86.0 (2.0494)	0.19 (0.0187)	0.91 (0.0126)	0.85 (0.0156)
	aligned_tcoffee	107.9 (4.6078)	0.35 (0.0271)	0.80 (0.0189)	0.71 (0.0230)
RD	correct	256.1 (7.2823)	0.54 (0.0115)	0.54 (0.0115)	0.52 (0.0109)
935 sequences	structaligned	195.3 (3.9131)	0.39 (0.0095)	0.81 (0.0084)	0.71 (0.0080)
	aligned_clustalw	162.9 (2.2083)	0.27 (0.0085)	0.96 (0.0058)	0.93 (0.0051)
	aligned_mafft	197.5 (4.9424)	0.40 (0.0119)	0.87 (0.0080)	0.76 (0.0129)
	aligned_msaprobs	208.8 (4.9549)	0.43 (0.0116)	0.75 (0.0126)	0.65 (0.0119)
	aligned_muscle	197.3 (3.9834)	0.40 (0.0121)	0.89 (0.0066)	0.77 (0.0118)
	aligned_probalign	209.0 (4.1339)	0.43 (0.0101)	0.74 (0.0080)	0.66 (0.0099)
	aligned_probcons	208.1 (4.4857)	0.43 (0.0111)	0.76 (0.0113)	0.67 (0.0131)
	aligned_tcoffee	212.4 (5.6592)	0.44 (0.0130)	0.78 (0.0115)	0.71 (0.0143)

Table S2. Structure-guided and sequence-alignment methods typically underestimate correct alignment lengths and gap proportions while overestimating the proportions of variable and parsimony-informative sites. We simulated alignments of 5 protein domain families, using empirically-derived simulation conditions, and aligned the resulting replicate sequence data using structure-guided and 7 different sequence-alignment methods (see Methods). For each alignment method and protein domain, we report the mean and standard error (in parentheses, over 10 replicates) in total alignment length (alignment_len), the proportions of gap characters (P(gap)), variable sites (P(variable)) and parsimony-informative sites (P(parsimony)). The number of sequences in each protein domain tree is also reported.

domain	alignment	meanDistance	medianDistance	highestMode	SEmean
CARD	structaligned	6.44E-01	6.55E-01	6.55E-01	5.15E-03
	aligned_clustalw	7.46E-01	7.59E-01	7.58E-01	8.43E-03
	aligned_mafft	7.42E-01	7.42E-01	7.44E-01	2.42E-03
	aligned_msaprobs	6.74E-01	6.63E-01	6.62E-01	8.05E-03
	aligned_muscle	7.94E-01	7.97E-01	7.97E-01	5.73E-03
	aligned_probalign	7.79E-01	7.78E-01	7.74E-01	5.25E-03
	aligned_probcons	6.47E-01	6.52E-01	6.54E-01	6.44E-03
	aligned_tcoffee	7.02E-01	7.06E-01	7.07E-01	4.16E-03
DSRM1	structaligned	1.03E-01	9.29E-02	7.76E-02	1.42E-02
	aligned_clustalw	3.36E-01	3.33E-01	3.22E-01	3.75E-02
	aligned_mafft	3.83E-01	3.92E-01	3.78E-01	3.23E-02
	aligned_msaprobs	1.93E-01	1.95E-01	2.05E-01	2.36E-02
	aligned_muscle	3.18E-01	3.18E-01	2.40E-01	3.19E-02
	aligned_probalign	5.38E-01	5.06E-01	4.85E-01	3.20E-02
	aligned_probcons	1.65E-01	1.57E-01	1.28E-01	2.59E-02
	aligned_tcoffee	2.65E-01	2.87E-01	3.22E-01	2.23E-02
DSRM2	structaligned	1.73E-01	1.57E-01	1.35E-01	2.66E-02
	aligned_clustalw	4.20E-01	4.42E-01	4.46E-01	2.24E-02
	aligned_mafft	4.97E-01	4.85E-01	4.82E-01	2.64E-02
	aligned_msaprobs	2.73E-01	2.55E-01	2.47E-01	1.93E-02
	aligned_muscle	4.03E-01	4.03E-01	3.91E-01	2.32E-02
	aligned_probalign	5.44E-01	5.34E-01	5.53E-01	2.69E-02
	aligned_probcons	2.43E-01	2.47E-01	2.44E-01	2.19E-02
	aligned_tcoffee	4.27E-01	4.18E-01	4.11E-01	1.62E-02
DSRM3	structaligned	2.08E-02	1.50E-02	1.27E-02	6.14E-03
	aligned_clustalw	1.10E-01	9.95E-02	6.88E-02	2.02E-02
	aligned_mafft	8.08E-02	3.16E-02	2.62E-02	2.83E-02
	aligned_msaprobs	5.54E-02	2.70E-02	2.83E-02	2.01E-02
	aligned_muscle	9.38E-02	5.12E-02	3.87E-02	2.82E-02
	aligned_probalign	2.50E-01	2.07E-01	1.57E-01	4.98E-02
	aligned_probcons	9.15E-02	3.84E-02	3.66E-02	3.37E-02
	aligned_tcoffee	1.00E-01	5.49E-02	5.22E-02	3.23E-02

RD	structaligned	1.93E-02	1.79E-02	1.35E-02	3.79E-03
	aligned_clustalw	1.68E-01	1.64E-01	1.58E-01	1.06E-02
	aligned_mafft	5.17E-02	5.39E-02	5.53E-02	4.18E-03
	aligned_msaprobs	4.37E-02	4.80E-02	5.35E-02	5.06E-03
	aligned_muscle	5.32E-02	5.79E-02	6.90E-02	7.82E-03
	aligned_probalign	4.96E-02	5.60E-02	5.79E-02	5.79E-03
	aligned_probcons	5.15E-02	5.67E-02	6.06E-02	7.01E-03
	aligned_tcoffee	9.12E-02	9.00E-02	8.99E-02	6.43E-03

Table S3. Alignment distances differ across alignment methods and protein domain families. We simulated alignments of 5 protein domain families, using empirically-derived simulation conditions, and aligned the resulting replicate sequence data using structure-guided and 7 different sequence-alignment methods (see Methods). For each sequence alignment, we measured the position-wise distance of that alignment to the correct simulated alignment, which estimates the probability of randomly selecting an incorrectly-aligned residue from the inferred alignment (see Methods). We estimated the distribution of alignment distances across replicate simulations using kernel density estimation. We report the mean (and standard error), median and mode of each alignment-distance distribution.

errorType	alignment	meanErrorRate	medianErrorRate	highestMode	SEmean
Total	aligned_clustalw	6.31E-02	6.12E-02	4.20E-02	3.41E-04
	aligned_mafft	5.89E-02	5.63E-02	5.68E-02	4.43E-04
	aligned_msaprobs	3.85E-02	2.99E-02	1.65E-02	3.61E-04
	aligned_muscle	4.97E-02	4.24E-02	3.21E-02	4.08E-04
	aligned_probalign	6.76E-02	6.09E-02	3.70E-02	6.06E-04
	aligned_probcons	3.84E-02	3.02E-02	1.73E-02	3.51E-04
	aligned_tcoffee	5.63E-02	5.16E-02	3.79E-02	3.17E-04
	structaligned	3.49E-02	2.45E-02	1.48E-02	3.86E-04
	correctaligned	7.85E-03	2.50E-03	8.23E-04	1.96E-04
	integratedaln	2.88E-02	2.07E-02	1.40E-02	2.81E-04
Residues	aligned_clustalw	2.66E-02	2.46E-02	2.53E-02	2.17E-04
	aligned_mafft	1.91E-02	1.81E-02	1.88E-02	1.99E-04
	aligned_msaprobs	1.61E-02	1.18E-02	5.30E-03	2.02E-04
	aligned_muscle	2.09E-02	1.42E-02	9.42E-03	2.32E-04
	aligned_probalign	2.57E-02	1.77E-02	9.42E-03	3.13E-04
	aligned_probcons	1.75E-02	1.36E-02	5.30E-03	2.17E-04
	aligned_tcoffee	1.59E-02	1.16E-02	5.89E-03	2.06E-04
	structaligned	1.31E-02	7.70E-03	4.12E-03	1.95E-04
	correctaligned	6.18E-03	8.00E-04	5.89E-04	1.95E-04
	integratedaln	1.52E-02	1.07E-02	5.30E-03	2.03E-04
Insertions	aligned_clustalw	1.82E-02	1.41E-02	1.25E-02	1.19E-04
	aligned_mafft	2.02E-02	1.87E-02	1.87E-02	1.65E-04
	aligned_msaprobs	1.12E-02	7.40E-03	6.18E-03	1.22E-04
	aligned_muscle	1.52E-02	1.20E-02	1.08E-02	1.54E-04
	aligned_probalign	2.10E-02	1.99E-02	1.45E-02	1.73E-04
	aligned_probcons	1.05E-02	7.50E-03	6.56E-03	1.13E-04
	aligned_tcoffee	2.03E-02	1.88E-02	1.62E-02	1.03E-04
	structaligned	1.10E-02	6.80E-03	6.37E-03	1.38E-04
	correctaligned	8.49E-04	0.00E+00	0.00E+00	1.95E-05
	integratedaln	3.65E-03	2.60E-03	2.32E-03	3.82E-05
Deletions	aligned_clustalw	1.83E-02	1.42E-02	1.27E-02	1.19E-04
	aligned_mafft	1.97E-02	1.86E-02	1.85E-02	1.58E-04
	aligned_msaprobs	1.12E-02	7.40E-03	6.24E-03	1.22E-04
	aligned_muscle	1.37E-02	1.17E-02	1.13E-02	1.21E-04
	aligned_probalign	2.09E-02	1.99E-02	1.44E-02	1.72E-04
	aligned_probcons	1.04E-02	7.40E-03	6.63E-03	1.10E-04
	aligned_tcoffee	2.01E-02	1.85E-02	1.62E-02	1.01E-04
	structaligned	1.09E-02	6.80E-03	6.44E-03	1.36E-04
	correctaligned	8.17E-04	0.00E+00	0.00E+00	1.87E-05
	integratedaln	9.99E-03	7.00E-03	6.44E-03	1.17E-04

Table S4. Ancestral sequence reconstruction (ASR) error rates are low when the correct alignment is known in advance, higher when the alignment is inferred using sequence-alignment methods, and intermediate when structure-guided alignments or alignment-integrated ASR is used. We simulated extant and ancestral sequences for 5 protein domain families, using empirically-derived conditions, and aligned the resulting extant sequence data using structure-guided and 7 different sequence-alignment methods (see Methods). We used each alignment to infer the most likely ancestral sequence at each node on the phylogeny. Additionally, alignment-integrated ancestral sequences were generated by combining inferences from the 7 sequence-alignment methods (see Methods). In each case, we compared the inferred ancestral sequences to the correct simulated sequence to estimate ancestral sequence reconstruction (ASR) error rates (see Methods). ASR errors were divided into 4 “errorType” categories: 1) residue errors, in which both correct and inferred ancestral sequences have a residue at a given position in the alignment, but the residues differ; 2) insertion errors, in which the inferred sequence has a residue at a given alignment position, but the correct sequence has

a gap state; 3) deletion errors, in which the inferred sequence has a gap, but the correct sequence has a residue, and 4) total errors, which combine residue- insertion- and deletion-errors. Sequence-wide error rates (errors/site) were computed by dividing the number of errors by the length of the pairwise alignment of the inferred and correct ancestral sequences. Error rate distributions were inferred by kernel density estimation. We report the mean (and standard error), median and mode of each ASR-error distribution.

domain	alignment	meanErrorRate	medianErrorRate	highestMode	SEmean
CARD	aligned_clustalw	9.21E-02	9.15E-02	8.95E-02	9.60E-05
	aligned_mafft	9.53E-02	1.03E-01	1.06E-01	4.68E-04
	aligned_msaprobs	7.50E-02	8.03E-02	5.17E-02	5.26E-04
	aligned_muscle	8.72E-02	9.83E-02	1.05E-01	6.51E-04
	aligned_probalign	8.57E-02	8.77E-02	6.22E-02	5.71E-04
	aligned_probcons	7.26E-02	7.52E-02	5.29E-02	4.63E-04
	aligned_tcoffee	8.34E-02	8.56E-02	6.62E-02	3.91E-04
	structaligned	8.04E-02	8.28E-02	6.45E-02	3.72E-04
	correctaligned	1.04E-02	8.40E-03	7.47E-03	1.86E-04
	integratedaln	5.56E-02	5.82E-02	4.27E-02	2.96E-04
DSRM1	aligned_clustalw	4.90E-02	4.07E-02	3.78E-02	4.34E-04
	aligned_mafft	5.52E-02	5.40E-02	3.49E-02	6.34E-04
	aligned_msaprobs	3.15E-02	2.61E-02	1.74E-02	4.33E-04
	aligned_muscle	4.47E-02	3.69E-02	2.81E-02	4.78E-04
	aligned_probalign	7.56E-02	6.61E-02	3.49E-02	1.00E-03
	aligned_probcons	2.76E-02	2.13E-02	1.71E-02	3.94E-04
	aligned_tcoffee	4.53E-02	4.08E-02	3.97E-02	3.97E-04
	structaligned	2.21E-02	1.63E-02	1.45E-02	3.70E-04
	correctaligned	7.49E-03	1.00E-03	9.68E-04	3.72E-04
	integratedaln	2.18E-02	1.78E-02	1.65E-02	3.53E-04
DSRM2	aligned_clustalw	5.44E-02	5.18E-02	4.33E-02	3.24E-04
	aligned_mafft	6.74E-02	6.41E-02	6.34E-02	4.69E-04
	aligned_msaprobs	3.26E-02	2.64E-02	2.52E-02	4.14E-04
	aligned_muscle	4.77E-02	3.71E-02	3.38E-02	4.48E-04
	aligned_probalign	6.58E-02	4.63E-02	4.22E-02	7.38E-04
	aligned_probcons	3.30E-02	3.03E-02	3.01E-02	3.50E-04
	aligned_tcoffee	5.61E-02	5.16E-02	5.05E-02	4.35E-04
	structaligned	3.01E-02	2.57E-02	2.50E-02	2.64E-04
	correctaligned	5.95E-03	8.00E-04	8.60E-04	2.87E-04
	integratedaln	2.36E-02	1.86E-02	1.72E-02	3.06E-04
DSRM3	aligned_clustalw	4.43E-02	2.35E-02	1.98E-02	1.40E-03
	aligned_mafft	2.91E-02	9.20E-03	6.59E-03	1.41E-03
	aligned_msaprobs	2.26E-02	9.20E-03	6.59E-03	1.09E-03
	aligned_muscle	3.11E-02	8.50E-03	6.59E-03	1.36E-03
	aligned_probalign	8.17E-02	1.94E-02	1.65E-02	3.39E-03
	aligned_probcons	3.03E-02	7.90E-03	5.76E-03	1.35E-03
	aligned_tcoffee	4.01E-02	2.28E-02	1.89E-02	1.25E-03
	structaligned	1.47E-02	5.90E-03	6.59E-03	1.02E-03
	correctaligned	8.09E-03	6.00E-04	8.23E-04	9.72E-04
	integratedaln	2.07E-02	5.10E-03	3.29E-03	1.10E-03
RD	aligned_clustalw	7.25E-02	6.84E-02	7.09E-02	7.57E-04
	aligned_mafft	2.42E-02	1.94E-02	1.64E-02	6.65E-04
	aligned_msaprobs	2.06E-02	1.59E-02	1.49E-02	5.60E-04
	aligned_muscle	2.33E-02	1.86E-02	1.74E-02	6.14E-04
	aligned_probalign	2.30E-02	1.97E-02	1.93E-02	5.54E-04
	aligned_probcons	2.22E-02	1.86E-02	1.69E-02	5.86E-04
	aligned_tcoffee	4.83E-02	4.44E-02	3.95E-02	5.45E-04
	structaligned	1.51E-02	9.70E-03	9.16E-03	5.51E-04
	correctaligned	7.83E-03	1.40E-03	1.45E-03	5.80E-04
	integratedaln	1.74E-02	1.32E-02	1.21E-02	5.53E-04

Table S5. Total ancestral sequence reconstruction (ASR) error rates are low when the correct alignment is known in advance, higher when the alignment is inferred using sequence-alignment methods, and intermediate when structure-guided alignments or alignment-integrated ASR is used. We simulated extant and ancestral sequences for 5 protein domain families, using empirically-derived conditions, and aligned the resulting extant sequence data using structure-guided and 7 different sequence-alignment methods (see

Methods). We used each alignment to infer the most likely ancestral sequence at each node on the phylogeny. Additionally, alignment-integrated ancestral sequences were generated by combining inferences from the 7 sequence-alignment methods (see Methods). In each case, we compared the inferred ancestral sequences to the correct simulated sequence to estimate ancestral sequence reconstruction (ASR) error rates (see Methods). ASR errors were divided into 4 "errorType" categories: 1) residue errors, in which both correct and inferred ancestral sequences have a residue at a given position in the alignment, but the residues differ; 2) insertion errors, in which the inferred sequence has a residue at a given alignment position, but the correct sequence has a gap state; 3) deletion errors, in which the inferred sequence has a gap, but the correct sequence has a residue, and 4) total errors, which combine residue- insertion- and deletion-errors. Sequence-wide error rates (errors/site) were computed by dividing the number of errors by the length of the pairwise alignment of the inferred and correct ancestral sequences. Error rate distributions were inferred by kernel density estimation. For each protein domain family, we report the mean (and standard error), median and mode of the total ASR-error distribution.

domain	alignment	meanErrorRate	medianErrorRate	highestMode	SEmean
CARD	aligned_clustalw	2.68E-02	2.62E-02	2.58E-02	7.65E-05
	aligned_mafft	2.07E-02	2.44E-02	2.67E-02	2.05E-04
	aligned_msaprobs	2.10E-02	2.38E-02	2.50E-02	2.25E-04
	aligned_muscle	2.50E-02	2.96E-02	3.18E-02	2.89E-04
	aligned_probalign	2.44E-02	2.48E-02	1.64E-02	2.03E-04
	aligned_probcons	2.29E-02	2.53E-02	2.64E-02	1.77E-04
	aligned_tcoffee	1.93E-02	2.03E-02	1.20E-02	1.70E-04
	structaligned	2.10E-02	2.45E-02	2.54E-02	1.82E-04
	correctaligned	3.37E-03	1.10E-03	8.70E-04	1.75E-04
	integratedaln	2.13E-02	2.48E-02	2.61E-02	2.05E-04
DSRM1	aligned_clustalw	2.61E-02	1.79E-02	1.66E-02	3.83E-04
	aligned_mafft	2.17E-02	1.83E-02	1.28E-02	3.60E-04
	aligned_msaprobs	1.61E-02	1.25E-02	1.13E-02	3.48E-04
	aligned_muscle	2.19E-02	1.59E-02	1.06E-02	3.75E-04
	aligned_probalign	2.83E-02	2.53E-02	7.45E-03	4.95E-04
	aligned_probcons	1.47E-02	9.70E-03	7.45E-03	3.47E-04
	aligned_tcoffee	1.39E-02	9.10E-03	7.45E-03	3.49E-04
	structaligned	1.08E-02	4.90E-03	4.26E-03	3.51E-04
	correctaligned	7.40E-03	9.00E-04	8.51E-04	3.71E-04
	integratedaln	1.31E-02	9.40E-03	6.81E-03	3.39E-04
DSRM2	aligned_clustalw	2.58E-02	2.37E-02	1.62E-02	2.68E-04
	aligned_mafft	2.25E-02	1.98E-02	1.85E-02	2.40E-04
	aligned_msaprobs	1.56E-02	1.06E-02	9.80E-03	3.08E-04
	aligned_muscle	2.03E-02	1.13E-02	9.44E-03	3.49E-04
	aligned_probalign	2.43E-02	1.44E-02	1.19E-02	3.79E-04
	aligned_probcons	1.62E-02	1.34E-02	1.32E-02	2.83E-04
	aligned_tcoffee	1.66E-02	1.14E-02	8.55E-03	3.46E-04
	structaligned	1.22E-02	7.80E-03	7.13E-03	2.48E-04
	correctaligned	5.78E-03	6.00E-04	5.35E-04	2.85E-04
	integratedaln	1.32E-02	8.60E-03	6.59E-03	2.86E-04
DSRM3	aligned_clustalw	2.86E-02	8.70E-03	7.07E-03	1.25E-03
	aligned_mafft	1.49E-02	5.10E-03	4.12E-03	9.87E-04
	aligned_msaprobs	1.52E-02	4.50E-03	2.95E-03	9.97E-04
	aligned_muscle	2.39E-02	5.30E-03	2.95E-03	1.21E-03
	aligned_probalign	4.30E-02	1.09E-02	5.89E-03	1.86E-03
	aligned_probcons	2.31E-02	4.15E-03	2.36E-03	1.20E-03
	aligned_tcoffee	1.91E-02	5.00E-03	2.36E-03	1.05E-03
	structaligned	9.25E-03	2.20E-03	2.36E-03	9.57E-04
	correctaligned	7.98E-03	5.00E-04	1.18E-03	9.66E-04
	integratedaln	1.79E-02	3.75E-03	1.77E-03	1.05E-03
RD	aligned_clustalw	2.68E-02	2.26E-02	2.28E-02	6.09E-04
	aligned_mafft	1.05E-02	5.40E-03	4.19E-03	5.67E-04
	aligned_msaprobs	1.10E-02	4.90E-03	4.95E-03	5.63E-04
	aligned_muscle	1.24E-02	6.40E-03	6.09E-03	5.78E-04
	aligned_probalign	1.25E-02	7.30E-03	6.47E-03	5.55E-04
	aligned_probcons	1.21E-02	6.60E-03	5.71E-03	5.69E-04
	aligned_tcoffee	1.09E-02	5.70E-03	4.95E-03	5.49E-04
	structaligned	9.76E-03	3.60E-03	3.81E-03	5.63E-04
	correctaligned	7.49E-03	1.00E-03	1.14E-03	5.75E-04
	integratedaln	1.11E-02	5.20E-03	4.95E-03	5.55E-04

Table S6. Ancestral sequence reconstruction (ASR) residue-reconstruction error rates are low when the correct alignment is known in advance, higher when the alignment is inferred using sequence-alignment methods, and intermediate when structure-guided alignments or alignment-integrated ASR is used. We simulated extant and ancestral sequences for 5 protein domain families, using empirically-derived conditions, and aligned the resulting extant sequence data using structure-guided and 7 different sequence-alignment

methods (see Methods). We used each alignment to infer the most likely ancestral sequence at each node on the phylogeny. Additionally, alignment-integrated ancestral sequences were generated by combining inferences from the 7 sequence-alignment methods (see Methods). In each case, we compared the inferred ancestral sequences to the correct simulated sequence to estimate ancestral sequence reconstruction (ASR) error rates (see Methods). ASR errors were divided into 4 “errorType” categories: 1) residue errors, in which both correct and inferred ancestral sequences have a residue at a given position in the alignment, but the residues differ; 2) insertion errors, in which the inferred sequence has a residue at a given alignment position, but the correct sequence has a gap state; 3) deletion errors, in which the inferred sequence has a gap, but the correct sequence has a residue, and 4) total errors, which combine residue- insertion- and deletion-errors. Sequence-wide error rates (errors/site) were computed by dividing the number of errors by the length of the pairwise alignment of the inferred and correct ancestral sequences. Error rate distributions were inferred by kernel density estimation. For each protein domain family, we report the mean (and standard error), median and mode of the residue ASR-error distribution.

domain	alignment	meanErrorRate	medianErrorRate	highestMode	SEmean
CARD	aligned_clustalw	3.26E-02	3.26E-02	3.22E-02	3.52E-05
	aligned_mafft	3.77E-02	3.81E-02	3.14E-02	1.67E-04
	aligned_msaprobs	2.70E-02	2.65E-02	2.05E-02	1.74E-04
	aligned_muscle	3.37E-02	3.56E-02	2.28E-02	2.60E-04
	aligned_probalign	3.08E-02	2.96E-02	2.30E-02	2.28E-04
	aligned_probcons	2.50E-02	2.42E-02	1.87E-02	1.70E-04
	aligned_tcoffee	3.23E-02	3.24E-02	2.71E-02	1.33E-04
	structaligned	2.98E-02	2.89E-02	2.59E-02	1.15E-04
	correctaligned	3.55E-03	3.30E-03	3.07E-03	1.99E-05
	integratedaln	8.74E-03	9.20E-03	9.81E-03	4.13E-05
DSRM1	aligned_clustalw	1.14E-02	1.13E-02	1.11E-02	3.90E-05
	aligned_mafft	1.70E-02	1.56E-02	1.13E-02	1.77E-04
	aligned_msaprobs	7.71E-03	6.50E-03	5.78E-03	7.39E-05
	aligned_muscle	1.17E-02	1.04E-02	9.45E-03	9.22E-05
	aligned_probalign	2.37E-02	2.03E-02	1.36E-02	2.75E-04
	aligned_probcons	6.48E-03	5.70E-03	5.23E-03	4.94E-05
	aligned_tcoffee	1.58E-02	1.55E-02	1.55E-02	7.17E-05
	structaligned	5.65E-03	5.60E-03	4.67E-03	2.61E-05
	correctaligned	5.14E-05	0.00E+00	0.00E+00	3.38E-06
	integratedaln	2.07E-03	1.80E-03	1.56E-03	2.06E-05
DSRM2	aligned_clustalw	1.43E-02	1.39E-02	1.30E-02	4.24E-05
	aligned_mafft	2.28E-02	2.21E-02	1.89E-02	1.47E-04
	aligned_msaprobs	8.56E-03	7.70E-03	7.62E-03	7.22E-05
	aligned_muscle	1.42E-02	1.28E-02	1.21E-02	8.42E-05
	aligned_probalign	2.08E-02	1.61E-02	1.51E-02	1.99E-04
	aligned_probcons	8.42E-03	8.20E-03	8.18E-03	5.31E-05
	aligned_tcoffee	2.00E-02	2.00E-02	2.03E-02	7.62E-05
	structaligned	8.93E-03	8.90E-03	8.96E-03	3.19E-05
	correctaligned	9.36E-05	0.00E+00	0.00E+00	5.13E-06
	integratedaln	2.50E-03	2.40E-03	2.35E-03	1.09E-05
DSRM3	aligned_clustalw	7.83E-03	7.80E-03	1.14E-02	1.08E-04
	aligned_mafft	7.10E-03	2.80E-03	1.74E-03	3.07E-04
	aligned_msaprobs	3.69E-03	2.20E-03	1.93E-03	9.47E-05
	aligned_muscle	3.55E-03	2.10E-03	1.54E-03	1.11E-04
	aligned_probalign	1.94E-02	5.60E-03	4.83E-03	7.89E-04
	aligned_probcons	3.55E-03	1.90E-03	1.74E-03	1.03E-04
	aligned_tcoffee	1.05E-02	8.50E-03	8.49E-03	1.62E-04
	structaligned	2.73E-03	1.40E-03	7.72E-04	1.05E-04
	correctaligned	4.55E-05	0.00E+00	0.00E+00	6.41E-06
	integratedaln	1.29E-03	1.00E-03	1.93E-04	4.31E-05
RD	aligned_clustalw	2.28E-02	2.26E-02	2.00E-02	1.02E-04
	aligned_mafft	6.83E-03	6.40E-03	3.84E-03	9.85E-05
	aligned_msaprobs	4.75E-03	4.70E-03	4.87E-03	4.96E-05
	aligned_muscle	5.36E-03	5.10E-03	5.83E-03	6.50E-05
	aligned_probalign	5.26E-03	5.10E-03	3.64E-03	5.73E-05
	aligned_probcons	5.04E-03	5.00E-03	3.22E-03	5.82E-05
	aligned_tcoffee	1.87E-02	1.81E-02	1.67E-02	8.59E-05
	structaligned	2.64E-03	2.70E-03	1.99E-03	2.90E-05
	correctaligned	1.72E-04	0.00E+00	0.00E+00	1.03E-05
	integratedaln	2.68E-03	2.60E-03	2.61E-03	3.26E-05

Table S7. Ancestral sequence reconstruction (ASR) insertion error rates are low when the correct alignment is known in advance, higher when the alignment is inferred using sequence-alignment methods, and intermediate when structure-guided alignments or alignment-integrated ASR is used. We simulated extant and ancestral sequences for 5 protein domain families, using empirically-derived conditions, and aligned the resulting extant sequence data using structure-guided and 7 different sequence-alignment methods (see

Methods). We used each alignment to infer the most likely ancestral sequence at each node on the phylogeny. Additionally, alignment-integrated ancestral sequences were generated by combining inferences from the 7 sequence-alignment methods (see Methods). In each case, we compared the inferred ancestral sequences to the correct simulated sequence to estimate ancestral sequence reconstruction (ASR) error rates (see Methods). ASR errors were divided into 4 “errorType” categories: 1) residue errors, in which both correct and inferred ancestral sequences have a residue at a given position in the alignment, but the residues differ; 2) insertion errors, in which the inferred sequence has a residue at a given alignment position, but the correct sequence has a gap state; 3) deletion errors, in which the inferred sequence has a gap, but the correct sequence has a residue, and 4) total errors, which combine residue- insertion- and deletion-errors. Sequence-wide error rates (errors/site) were computed by dividing the number of errors by the length of the pairwise alignment of the inferred and correct ancestral sequences. Error rate distributions were inferred by kernel density estimation. For each protein domain family, we report the mean (and standard error), median and mode of the insertion ASR-error distribution.

domain	alignment	meanErrorRate	medianErrorRate	highestMode	SEmean
CARD	aligned_clustalw	3.27E-02	3.27E-02	3.21E-02	3.25E-05
	aligned_mafft	3.70E-02	3.78E-02	3.13E-02	1.30E-04
	aligned_msaprobs	2.69E-02	2.62E-02	2.05E-02	1.73E-04
	aligned_muscle	2.85E-02	2.89E-02	2.25E-02	1.53E-04
	aligned_probalign	3.06E-02	2.95E-02	2.29E-02	2.25E-04
	aligned_probcons	2.46E-02	2.39E-02	1.87E-02	1.61E-04
	aligned_tcoffee	3.18E-02	3.19E-02	2.70E-02	1.27E-04
	structaligned	2.95E-02	2.86E-02	2.59E-02	1.05E-04
	correctaligned	3.44E-03	3.30E-03	3.17E-03	1.69E-05
	integratedaln	2.55E-02	2.47E-02	2.08E-02	1.42E-04
DSRM1	aligned_clustalw	1.15E-02	1.13E-02	1.12E-02	3.81E-05
	aligned_mafft	1.65E-02	1.52E-02	1.13E-02	1.57E-04
	aligned_msaprobs	7.70E-03	6.50E-03	5.77E-03	7.37E-05
	aligned_muscle	1.12E-02	1.02E-02	9.71E-03	7.36E-05
	aligned_probalign	2.36E-02	2.03E-02	1.36E-02	2.73E-04
	aligned_probcons	6.48E-03	5.70E-03	5.22E-03	4.91E-05
	aligned_tcoffee	1.56E-02	1.54E-02	1.55E-02	7.40E-05
	structaligned	5.68E-03	5.70E-03	4.67E-03	2.63E-05
	correctaligned	4.75E-05	0.00E+00	0.00E+00	3.71E-06
	integratedaln	6.61E-03	6.50E-03	4.85E-03	4.18E-05
DSRM2	aligned_clustalw	1.43E-02	1.39E-02	1.31E-02	4.18E-05
	aligned_mafft	2.21E-02	2.19E-02	1.88E-02	1.23E-04
	aligned_msaprobs	8.51E-03	7.70E-03	7.62E-03	7.12E-05
	aligned_muscle	1.32E-02	1.24E-02	1.20E-02	5.81E-05
	aligned_probalign	2.07E-02	1.60E-02	1.51E-02	1.98E-04
	aligned_probcons	8.39E-03	8.20E-03	8.23E-03	5.25E-05
	aligned_tcoffee	1.96E-02	1.99E-02	2.02E-02	7.65E-05
	structaligned	8.91E-03	8.90E-03	8.94E-03	3.09E-05
	correctaligned	6.94E-05	0.00E+00	0.00E+00	3.45E-06
	integratedaln	7.87E-03	7.60E-03	7.53E-03	4.42E-05
DSRM3	aligned_clustalw	7.89E-03	7.80E-03	1.15E-02	1.09E-04
	aligned_mafft	7.12E-03	2.80E-03	1.76E-03	3.09E-04
	aligned_msaprobs	3.70E-03	2.20E-03	1.95E-03	9.53E-05
	aligned_muscle	3.63E-03	2.10E-03	1.56E-03	1.14E-04
	aligned_probalign	1.94E-02	5.60E-03	4.88E-03	7.89E-04
	aligned_probcons	3.57E-03	1.90E-03	1.76E-03	1.05E-04
	aligned_tcoffee	1.05E-02	8.50E-03	8.58E-03	1.64E-04
	structaligned	2.75E-03	1.40E-03	7.80E-04	1.07E-04
	correctaligned	5.89E-05	0.00E+00	0.00E+00	8.09E-06
	integratedaln	1.57E-03	1.00E-03	9.75E-04	4.80E-05
RD	aligned_clustalw	2.29E-02	2.26E-02	2.01E-02	1.04E-04
	aligned_mafft	6.89E-03	6.40E-03	3.84E-03	1.01E-04
	aligned_msaprobs	4.78E-03	4.70E-03	4.80E-03	4.91E-05
	aligned_muscle	5.55E-03	5.40E-03	5.69E-03	6.96E-05
	aligned_probalign	5.29E-03	5.10E-03	3.64E-03	5.69E-05
	aligned_probcons	5.08E-03	5.00E-03	3.22E-03	5.82E-05
	aligned_tcoffee	1.87E-02	1.81E-02	1.66E-02	8.59E-05
	structaligned	2.67E-03	2.70E-03	1.99E-03	2.81E-05
	correctaligned	1.62E-04	0.00E+00	0.00E+00	7.31E-06
	integratedaln	3.68E-03	3.50E-03	3.02E-03	5.24E-05

Table S8. Ancestral sequence reconstruction (ASR) deletion error rates are low when the correct alignment is known in advance, higher when the alignment is inferred using sequence-alignment methods, and intermediate when structure-guided alignments or alignment-integrated ASR is used. We simulated extant and ancestral sequences for 5 protein domain families, using empirically-derived conditions, and aligned the resulting extant sequence data using structure-guided and 7 different sequence-alignment methods (see

Methods). We used each alignment to infer the most likely ancestral sequence at each node on the phylogeny. Additionally, alignment-integrated ancestral sequences were generated by combining inferences from the 7 sequence-alignment methods (see Methods). In each case, we compared the inferred ancestral sequences to the correct simulated sequence to estimate ancestral sequence reconstruction (ASR) error rates (see Methods). ASR errors were divided into 4 “errorType” categories: 1) residue errors, in which both correct and inferred ancestral sequences have a residue at a given position in the alignment, but the residues differ; 2) insertion errors, in which the inferred sequence has a residue at a given alignment position, but the correct sequence has a gap state; 3) deletion errors, in which the inferred sequence has a gap, but the correct sequence has a residue, and 4) total errors, which combine residue- insertion- and deletion-errors. Sequence-wide error rates (errors/site) were computed by dividing the number of errors by the length of the pairwise alignment of the inferred and correct ancestral sequences. Error rate distributions were inferred by kernel density estimation. For each protein domain family, we report the mean (and standard error), median and mode of the deletion ASR-error distribution.

errorType	alignment	meanErrorPP	medianErrorPP	highestMode	SEmean
Total	aligned_clustalw	9.48E-01	9.94E-01	9.96E-01	1.36E-03
	aligned_mafft	9.37E-01	9.92E-01	9.94E-01	1.50E-03
	aligned_msaprobs	9.24E-01	9.83E-01	9.94E-01	1.56E-03
	aligned_muscle	9.02E-01	9.64E-01	9.90E-01	1.72E-03
	aligned_probalign	9.35E-01	9.89E-01	9.94E-01	1.43E-03
	aligned_probcons	9.22E-01	9.83E-01	9.94E-01	1.58E-03
	aligned_tcoffee	9.41E-01	9.92E-01	9.94E-01	1.47E-03
	structaligned	9.21E-01	9.84E-01	9.94E-01	1.55E-03
	correctaligned	6.46E-01	7.05E-01	6.82E-01	4.28E-03
	integratedaln	6.74E-01	6.86E-01	7.04E-01	1.35E-03
Residues	aligned_clustalw	9.23E-01	9.86E-01	9.94E-01	1.72E-03
	aligned_mafft	9.03E-01	9.78E-01	9.94E-01	1.88E-03
	aligned_msaprobs	8.91E-01	9.59E-01	9.94E-01	1.90E-03
	aligned_muscle	8.71E-01	9.40E-01	9.88E-01	2.11E-03
	aligned_probalign	9.05E-01	9.75E-01	9.92E-01	1.84E-03
	aligned_probcons	8.86E-01	9.58E-01	9.92E-01	2.07E-03
	aligned_tcoffee	8.93E-01	9.62E-01	9.92E-01	1.94E-03
	structaligned	8.89E-01	9.53E-01	9.94E-01	1.85E-03
	correctaligned	5.66E-01	6.56E-01	6.66E-01	4.33E-03
	integratedaln	5.86E-01	6.05E-01	6.18E-01	1.54E-03
Insertions	aligned_clustalw	9.50E-01	9.97E-01	9.96E-01	1.55E-03
	aligned_mafft	9.48E-01	9.98E-01	9.96E-01	1.65E-03
	aligned_msaprobs	9.43E-01	9.99E-01	9.96E-01	1.63E-03
	aligned_muscle	9.07E-01	9.85E-01	9.92E-01	2.04E-03
	aligned_probalign	9.40E-01	9.97E-01	9.96E-01	1.69E-03
	aligned_probcons	9.47E-01	9.99E-01	9.96E-01	1.55E-03
	aligned_tcoffee	9.42E-01	9.96E-01	9.96E-01	1.68E-03
	structaligned	9.54E-01	1.00E+00	9.98E-01	1.45E-03
	correctaligned	4.21E-01	0.00E+00	0.00E+00	6.30E-03
	integratedaln	7.17E-01	8.00E-01	8.58E-01	2.84E-03
Deletions	aligned_clustalw	9.98E-01	1.00E+00	1.00E+00	1.03E-04
	aligned_mafft	9.96E-01	1.00E+00	1.00E+00	2.68E-04
	aligned_msaprobs	9.99E-01	1.00E+00	1.00E+00	8.91E-05
	aligned_muscle	9.86E-01	1.00E+00	1.00E+00	3.10E-04
	aligned_probalign	9.98E-01	1.00E+00	1.00E+00	1.30E-04
	aligned_probcons	9.98E-01	1.00E+00	1.00E+00	1.12E-04
	aligned_tcoffee	9.97E-01	1.00E+00	1.00E+00	1.90E-04
	structaligned	9.97E-01	1.00E+00	1.00E+00	4.35E-04
	correctaligned	4.29E-01	0.00E+00	0.00E+00	6.47E-03
	integratedaln	7.46E-01	7.42E-01	7.32E-01	1.36E-03

Table S9. Support for erroneously-inferred ancestral states is reduced by alignment-integrated ancestral sequence reconstruction. We simulated extant and ancestral sequences for 5 protein domain families, using empirically-derived conditions, and aligned the resulting extant sequence data using structure-guided and 7 different sequence-alignment methods (see Methods). We used each alignment to infer the most likely ancestral sequence at each node on the phylogeny. Additionally, alignment-integrated ancestral sequences were generated by combining inferences from the 7 sequence-alignment methods (see Methods). In each case, we compared the inferred ancestral sequences to the correct simulated sequence to estimate ancestral sequence reconstruction (ASR) error rates (see Methods). ASR errors were divided into 4 “errorType” categories: 1) residue errors, in which both correct and inferred ancestral sequences have a residue at a given position in the alignment, but the residues differ; 2) insertion errors, in which the inferred sequence has a residue at a given alignment position, but the correct sequence has a gap state; 3) deletion errors, in which the inferred sequence has a gap, but the correct sequence has a residue, and 4) total errors, which combine residue-

insertion- and deletion-errors. The posterior probability (PP) of each erroneously-inferred ancestral state was determined using an empirical-Bayesian approach (see Methods). The distribution of posterior probabilities for erroneously-inferred ancestral states was calculated by kernel density estimation. We report the mean (and standard error), median and mode of the posterior-probability distribution for each type of ASR error.

errorType	alignment	meanCorrectPP	medianCorrectPP	highestMode	SEmean
Total	aligned_clustalw	1.64E-02	2.60E-03	2.00E-03	3.94E-04
	aligned_mafft	2.66E-02	2.80E-03	2.00E-03	6.36E-04
	aligned_msaprobs	3.33E-02	7.20E-03	2.00E-03	6.48E-04
	aligned_muscle	4.53E-02	2.81E-02	4.00E-03	6.80E-04
	aligned_probalign	2.35E-02	3.90E-03	2.00E-03	5.70E-04
	aligned_probcons	3.68E-02	8.80E-03	2.00E-03	6.87E-04
	aligned_tcoffee	2.09E-02	4.00E-03	2.00E-03	4.61E-04
	structaligned	4.23E-02	8.10E-03	2.00E-03	8.03E-04
	correctaligned	1.39E-01	1.34E-01	2.00E-03	1.75E-03
	integratedaln	3.29E-01	3.29E-01	4.30E-01	1.69E-03
Residues	aligned_clustalw	2.98E-02	6.70E-03	1.99E-03	5.92E-04
	aligned_mafft	4.80E-02	9.90E-03	1.99E-03	9.13E-04
	aligned_msaprobs	5.49E-02	2.21E-02	2.99E-03	9.06E-04
	aligned_muscle	5.01E-02	2.21E-02	3.98E-03	8.62E-04
	aligned_probalign	3.81E-02	8.60E-03	1.99E-03	7.78E-04
	aligned_probcons	5.72E-02	2.19E-02	2.99E-03	9.64E-04
	aligned_tcoffee	5.29E-02	1.97E-02	1.99E-03	8.88E-04
	structaligned	6.52E-02	2.77E-02	1.99E-03	1.01E-03
	correctaligned	1.61E-01	1.69E-01	9.95E-04	1.83E-03
	integratedaln	2.06E-01	2.12E-01	2.35E-01	1.11E-03
Insertions	aligned_clustalw	2.09E-03	0.00E+00	0.00E+00	1.39E-04
	aligned_mafft	5.10E-03	0.00E+00	0.00E+00	3.40E-04
	aligned_msaprobs	1.25E-03	0.00E+00	0.00E+00	9.21E-05
	aligned_muscle	2.28E-02	2.00E-04	9.88E-04	4.98E-04
	aligned_probalign	1.71E-03	0.00E+00	0.00E+00	1.15E-04
	aligned_probcons	2.02E-03	0.00E+00	0.00E+00	1.30E-04
	aligned_tcoffee	4.04E-03	0.00E+00	0.00E+00	2.23E-04
	structaligned	2.47E-03	0.00E+00	0.00E+00	2.09E-04
	correctaligned	9.90E-03	0.00E+00	0.00E+00	6.90E-04
	integratedaln	2.97E-01	3.10E-01	3.09E-01	1.02E-03
Deletions	aligned_clustalw	4.59E-03	0.00E+00	0.00E+00	2.59E-04
	aligned_mafft	4.39E-03	0.00E+00	0.00E+00	2.62E-04
	aligned_msaprobs	3.06E-03	0.00E+00	0.00E+00	2.10E-04
	aligned_muscle	3.22E-02	1.87E-02	2.00E-03	6.86E-04
	aligned_probalign	6.98E-03	0.00E+00	0.00E+00	3.23E-04
	aligned_probcons	5.38E-03	0.00E+00	0.00E+00	2.63E-04
	aligned_tcoffee	8.14E-03	0.00E+00	0.00E+00	3.48E-04
	structaligned	4.39E-03	0.00E+00	0.00E+00	4.07E-04
	correctaligned	8.41E-03	0.00E+00	0.00E+00	9.29E-04
	integratedaln	4.63E-01	4.93E-01	5.14E-01	2.89E-03

Table S10. Support for the correct ancestral state is increased by alignment-integrated ancestral sequence reconstruction when an incorrect ancestral state is inferred. We simulated extant and ancestral sequences for 5 protein domain families, using empirically-derived conditions, and aligned the resulting extant sequence data using structure-guided and 7 different sequence-alignment methods (see Methods). We used each alignment to infer the most likely ancestral sequence at each node on the phylogeny. Additionally, alignment-integrated ancestral sequences were generated by combining inferences from the 7 sequence-alignment methods (see Methods). In each case, we compared the inferred ancestral sequences to the correct simulated sequence to estimate ancestral sequence reconstruction (ASR) error rates (see Methods). ASR errors were divided into 4 “errorType” categories: 1) residue errors, in which both correct and inferred ancestral sequences have a residue at a given position in the alignment, but the residues differ; 2) insertion errors, in which the inferred sequence has a residue at a given alignment position, but the correct sequence has a gap state; 3) deletion errors, in which the inferred sequence has a gap, but the correct sequence has a

residue, and 4) total errors, which combine residue- insertion- and deletion-errors. For each error type, when an erroneous ancestral state of that error type was inferred as the maximum-likelihood state, we calculated the posterior probability (PP) of the correct state using an empirical-Bayesian approach (see Methods). The distribution of posterior probabilities for the correct ancestral state, when ASR errors of each type were made, was inferred by kernel density estimation. We report the mean (and standard error), median and mode of the posterior-probability distribution of correct ancestral states for each type of ASR error.

ancState Type	alignment	meanCorrectPP	medianCorrectPP	highestMode	SEmean
Total	aligned_clustalw	9.98E-01	1.00E+00	1.00E+00	8.60E-05
	aligned_mafft	9.97E-01	1.00E+00	1.00E+00	1.05E-04
	aligned_msaprobs	9.97E-01	1.00E+00	1.00E+00	9.17E-05
	aligned_muscle	9.95E-01	9.99E-01	9.99E-01	1.05E-04
	aligned_probalign	9.98E-01	1.00E+00	1.00E+00	8.34E-05
	aligned_probcons	9.97E-01	1.00E+00	1.00E+00	9.21E-05
	aligned_tcoffee	9.97E-01	1.00E+00	1.00E+00	9.85E-05
	structaligned	9.97E-01	1.00E+00	1.00E+00	9.31E-05
	correctaligned	9.97E-01	1.00E+00	1.00E+00	9.56E-05
	integratedaln	9.74E-01	9.76E-01	9.80E-01	1.75E-04
Residues	aligned_clustalw	9.65E-01	9.98E-01	9.97E-01	1.19E-03
	aligned_mafft	9.63E-01	9.98E-01	9.97E-01	1.25E-03
	aligned_msaprobs	9.64E-01	9.98E-01	9.97E-01	1.11E-03
	aligned_muscle	9.48E-01	9.94E-01	9.95E-01	1.31E-03
	aligned_probalign	9.56E-01	9.98E-01	9.97E-01	1.35E-03
	aligned_probcons	9.65E-01	9.98E-01	9.97E-01	1.10E-03
	aligned_tcoffee	9.60E-01	9.97E-01	9.97E-01	1.24E-03
	structaligned	9.69E-01	9.98E-01	9.97E-01	9.71E-04
	correctaligned	9.69E-01	9.98E-01	9.97E-01	9.69E-04
	integratedaln	8.39E-01	8.93E-01	9.07E-01	1.87E-03
Gaps	aligned_clustalw	1.00E+00	1.00E+00	1.00E+00	2.76E-06
	aligned_mafft	9.99E-01	1.00E+00	1.00E+00	4.02E-05
	aligned_msaprobs	1.00E+00	1.00E+00	1.00E+00	3.27E-06
	aligned_muscle	9.99E-01	1.00E+00	1.00E+00	4.18E-05
	aligned_probalign	1.00E+00	1.00E+00	1.00E+00	1.31E-05
	aligned_probcons	1.00E+00	1.00E+00	1.00E+00	9.23E-06
	aligned_tcoffee	1.00E+00	1.00E+00	1.00E+00	1.60E-05
	structaligned	1.00E+00	1.00E+00	1.00E+00	8.91E-06
	correctaligned	1.00E+00	1.00E+00	1.00E+00	6.77E-07
	integratedaln	9.83E-01	9.86E-01	9.87E-01	1.05E-04

Table S11. Support for the correct ancestral state is reduced by alignment-integrated ancestral sequence reconstruction when the correct state is inferred by maximum-likelihood. We simulated extant and ancestral sequences for 5 protein domain families, using empirically-derived conditions, and aligned the resulting extant sequence data using structure-guided and 7 different sequence-alignment methods (see Methods). We used each alignment to infer the most likely ancestral sequence at each node on the phylogeny. Additionally, alignment-integrated ancestral sequences were generated by combining inferences from the 7 sequence-alignment methods (see Methods). In each case, we compared the inferred ancestral sequences to the correct simulated sequence to identify ancestral sequence reconstruction (ASR) errors (see Methods). When the correct ancestral state was inferred as the maximum-likelihood state, we calculated the posterior probability (PP) of the correct state using an empirical-Bayesian approach (see Methods). The distribution of posterior probabilities for the correct ancestral state, when no ASR error was made, was inferred by kernel density estimation. We report the mean (and standard error), median and mode of the posterior-probability distribution of correct ancestral states for each type of ancestral state (residues, gaps).

Property	alignment	meanError	medianError	highestMode	SEmean
Structural Stability	aligned_clustalw	3.81E-02	3.74E-02	3.95E-02	3.39E-04
	aligned_mafft	4.58E-02	4.38E-02	4.22E-02	3.78E-04
	aligned_msaprobs	2.95E-02	2.79E-02	2.63E-02	2.80E-04
	aligned_muscle	3.65E-02	3.32E-02	2.77E-02	3.53E-04
	aligned_probalign	5.12E-02	4.92E-02	4.33E-02	4.01E-04
	aligned_probcons	2.75E-02	2.43E-02	2.05E-02	3.02E-04
	aligned_tcoffee	3.34E-02	3.18E-02	3.12E-02	2.70E-04
	structaligned	2.51E-02	2.35E-02	2.32E-02	2.72E-04
	correctaligned	1.40E-02	1.01E-02	7.32E-03	3.11E-04
	integratedaln	2.60E-02	2.35E-02	2.01E-02	2.87E-04
Binding Affinity	aligned_clustalw	4.58E-01	4.58E-01	4.73E-01	3.22E-03
	aligned_mafft	4.84E-01	4.27E-01	4.03E-01	5.43E-03
	aligned_msaprobs	3.26E-01	3.05E-01	2.73E-01	3.41E-03
	aligned_muscle	4.10E-01	3.93E-01	3.67E-01	2.93E-03
	aligned_probalign	8.25E-01	7.37E-01	7.38E-01	1.22E-02
	aligned_probcons	3.55E-01	3.48E-01	3.50E-01	2.69E-03
	aligned_tcoffee	3.70E-01	3.30E-01	2.79E-01	3.99E-03
	structaligned	3.46E-01	3.30E-01	3.03E-01	3.03E-03
	correctaligned	1.70E-01	1.42E-01	1.08E-01	2.62E-03
	integratedaln	3.13E-01	2.99E-01	2.61E-01	2.70E-03

Table S12. Structure-guided and alignment-integrated methods reduce errors in estimated structural and functional properties of ancestral sequences, compared to sequence-alignment methods. We simulated replicate extant and ancestral sequence data sets by evolving the DSRM1 protein domain family along its empirically-determined phylogeny, using a structure-guided alignment of empirical DSRM1 sequences to determine the amino-acid composition and pattern of insertions/deletions (see Methods). Structural and functional properties were unconstrained during the simulations. We modeled the structure of each simulated ancestral sequence and estimated its structural stability (ΔG) and dsRNA-binding affinity (pKd) using computational approaches (see Methods). For each alignment method, we inferred the maximum-likelihood ancestral sequence at each node on the phylogeny, modeled the structure of the inferred ancestral sequence at each node, and estimated structural stability and binding affinity from the modeled structure. Structural stability and binding affinity errors were calculated as the absolute value of the difference between the value calculated from the correct simulated ancestral sequence and that calculated from the inferred ancestral sequence. The distributions of structural-stability and binding-affinity errors were calculated by kernel density estimation. We report the mean (and standard error), median and mode of each error distribution.

Structural Stability		linear regression				Binding Affinity		linear regression			
errorType	alignment	slope	intercept	rsquared	SEslope	errorType	alignment	slope	intercept	rsquared	SEslope
Total	aligned_clustalw	0.4149	0.0177	0.2828	5.64E-03	Total	aligned_clustalw	1.0682	0.406	0.0208	6.26E-02
	aligned_mafft	0.2011	0.0347	0.1135	4.80E-03		aligned_mafft	4.599	0.2302	0.2886	6.16E-02
	aligned_msaprobs	0.4331	0.0158	0.4482	4.10E-03		aligned_msaprobs	5.1293	0.1642	0.4238	5.10E-02
	aligned_muscle	0.4757	0.0152	0.4142	4.83E-03		aligned_muscle	2.2903	0.3071	0.1394	4.86E-02
	aligned_probalign	0.1684	0.0385	0.1771	3.10E-03		aligned_probalign	7.0038	0.2962	0.3337	8.44E-02
	aligned_probcons	0.5531	0.0122	0.5207	4.53E-03		aligned_probcons	3.4615	0.2597	0.2562	5.03E-02
	aligned_tcoffee	0.3193	0.019	0.2203	5.13E-03		aligned_tcoffee	5.1166	0.1383	0.2581	7.40E-02
	structaligned	0.5173	0.0136	0.4956	4.45E-03		structaligned	4.0694	0.2558	0.2471	6.06E-02
	correctaligned	0.7388	0.0085	0.7793	3.35E-03		correctaligned	5.6261	0.1274	0.6371	3.62E-02
	integratedaln	0.6029	0.0128	0.5495	4.66E-03		integratedaln	4.1519	0.2221	0.2935	5.50E-02
Residues	aligned_clustalw	0.4498	0.0263	0.2596	6.48E-03	Residues	aligned_clustalw	1.3831	0.4223	0.0272	7.06E-02
	aligned_mafft	0.4367	0.0363	0.1723	8.17E-03		aligned_mafft	7.2244	0.3271	0.2293	1.13E-01
	aligned_msaprobs	0.5147	0.0212	0.4089	5.28E-03		aligned_msaprobs	5.3511	0.2396	0.2979	7.01E-02
	aligned_muscle	0.5907	0.0236	0.3928	6.27E-03		aligned_muscle	2.528	0.3542	0.1045	6.32E-02
	aligned_probalign	0.32	0.0421	0.1559	6.35E-03		aligned_probalign	13.6434	0.4396	0.3085	1.74E-01
	aligned_probcons	0.5895	0.0189	0.4579	5.47E-03		aligned_probcons	3.6324	0.302	0.2184	5.86E-02
	aligned_tcoffee	0.4168	0.0276	0.2906	5.56E-03		aligned_tcoffee	4.0606	0.3138	0.1259	9.13E-02
	structaligned	0.5626	0.019	0.526	4.56E-03		structaligned	3.9771	0.3029	0.2117	6.55E-02
	correctaligned	0.74	0.0085	0.7779	3.37E-03		correctaligned	5.6492	0.1278	0.6391	3.62E-02
	integratedaln	0.6054	0.018	0.5111	5.05E-03		integratedaln	3.8968	0.2615	0.2386	5.94E-02
Insertions	aligned_clustalw	3.5195	-0.002	0.1648	6.76E-02	Insertions	aligned_clustalw	-3.4471	0.4977	0.0018	7.02E-01
	aligned_mafft	0.5275	0.0368	0.0612	1.76E-02		aligned_mafft	15.9376	0.2128	0.2716	2.23E-01
	aligned_msaprobs	1.7365	0.0161	0.2098	2.88E-02		aligned_msaprobs	28.7101	0.1048	0.3867	3.09E-01
	aligned_muscle	1.6341	0.0174	0.1822	2.95E-02		aligned_muscle	11.2059	0.2785	0.1244	2.54E-01
	aligned_probalign	0.6086	0.0368	0.174	1.13E-02		aligned_probalign	24.651	0.2413	0.3109	3.13E-01
	aligned_probcons	3.0809	0.0075	0.2543	4.50E-02		aligned_probcons	20.7379	0.2208	0.1447	4.30E-01
	aligned_tcoffee	0.1163	0.0316	0.001	3.21E-02		aligned_tcoffee	31.1141	-0.1216	0.3119	3.94E-01
	structaligned	0.8825	0.0201	0.0072	8.87E-02		structaligned	48.5075	0.0716	0.174	9.02E-01
	correctaligned	15.692	0.0132	0.029	7.75E-01		correctaligned	55.4388	0.1667	0.0051	6.61E+00
	integratedaln	3.5127	0.0187	0.0639	1.15E-01		integratedaln	33.1239	0.244	0.064	1.08E+00
Deletions	aligned_clustalw	4.5652	-0.0144	0.2634	6.51E-02	Deletions	aligned_clustalw	2.5011	0.4297	0.0009	7.20E-01
	aligned_mafft	0.3128	0.0407	0.0168	2.04E-02		aligned_mafft	16.7955	0.2078	0.2348	2.59E-01
	aligned_msaprobs	1.7272	0.0162	0.2066	2.89E-02		aligned_msaprobs	28.7887	0.1044	0.387	3.09E-01
	aligned_muscle	2.1685	0.0123	0.2041	3.65E-02		aligned_muscle	13.372	0.2601	0.1127	3.20E-01
	aligned_probalign	0.6004	0.037	0.1676	1.14E-02		aligned_probalign	24.5637	0.2459	0.3055	3.16E-01
	aligned_probcons	3.1011	0.0074	0.2542	4.53E-02		aligned_probcons	20.6997	0.2212	0.1423	4.34E-01
	aligned_tcoffee	-0.2079	0.0367	0.0032	3.11E-02		aligned_tcoffee	27.4858	-0.0589	0.2592	3.96E-01
	structaligned	1.5557	0.0162	0.0227	8.71E-02		structaligned	50.9999	0.0561	0.1963	8.80E-01
	correctaligned	13.362	0.0134	0.0254	7.06E-01		correctaligned	18.2189	0.1687	0.0007	6.02E+00
	integratedaln	2.285	0.0109	0.111	5.52E-02		integratedaln	31.1916	0.1065	0.2329	4.83E-01

Table S13. Errors in estimated structural stability and binding affinity of ancestral sequences are weakly correlated with errors in the inferred ancestral sequences. We simulated replicate extant and ancestral sequence data sets by evolving the DSRM1 protein domain family along its empirically-determined phylogeny, using a structure-guided alignment of empirical DSRM1 sequences to determine the amino-acid composition and pattern of insertions/ deletions (see Methods). Structural and functional properties were unconstrained during the simulations. We modeled the structure of each simulated ancestral sequence and estimated its structural stability (ΔG) and dsRNA-binding affinity (pKd) using computational approaches (see Methods). For each alignment method, we inferred the maximum-likelihood ancestral sequence at each node on the phylogeny, modeled the structure of the inferred ancestral sequence at each node, and estimated structural stability and binding affinity from the modeled structure. Structural stability and binding affinity errors were calculated as the absolute value of the difference between the value calculated from the correct simulated ancestral sequence and that calculated from the inferred ancestral sequence. ASR errors were divided into 4 “errorType”

categories: 1) residue errors, in which both correct and inferred ancestral sequences have a residue at a given position in the alignment, but the residues differ; 2) insertion errors, in which the inferred sequence has a residue at a given alignment position, but the correct sequence has a gap state; 3) deletion errors, in which the inferred sequence has a gap, but the correct sequence has a residue, and 4) total errors, which combine residue- insertion- and deletion-errors. Sequence-wide error rates (errors/site) were computed by dividing the number of errors by the length of the pairwise alignment of the inferred and correct ancestral sequences. For each type of ASR error and alignment method, we determined the best-fit linear relationship between ASR error rate and structural stability (left) or binding affinity (right) using ordinary least squares linear regression. We report the slope (and standard error), intercept and correlation (rsquared) of each linear regression.

IndelRate	Alignment	Slope	SE(Slope)	P(Slope>0)	Intercept	r^2
0.01	aligned_clustalw	0.5008	4.29E-02	2.38E-05	0.08	0.9578
	aligned_mafft	0.4825	4.15E-02	2.44E-05	0.0802	0.9575
	aligned_msaprobs	0.4871	4.18E-02	2.41E-05	0.0816	0.9577
	aligned_muscle	0.4876	4.38E-02	3.12E-05	0.0813	0.9539
	aligned_probalign	0.4941	4.16E-02	2.17E-05	0.0799	0.9591
	aligned_probcons	0.4866	4.31E-02	2.87E-05	0.081	0.9551
	aligned_tcoffee	0.4921	4.28E-02	2.59E-05	0.0804	0.9567
	correctaligned	0.4725	4.47E-02	4.19E-05	0.0819	0.9491
	integratedaln	0.4623	4.46E-02	4.69E-05	0.0847	0.9472
0.02	aligned_clustalw	0.4992	5.02E-02	5.97E-05	0.0882	0.9428
	aligned_mafft	0.4779	4.74E-02	5.49E-05	0.0861	0.9444
	aligned_msaprobs	0.4838	4.78E-02	5.43E-05	0.0862	0.9446
	aligned_muscle	0.4916	4.73E-02	4.67E-05	0.0825	0.9473
	aligned_probalign	0.4944	4.75E-02	4.63E-05	0.0851	0.9474
	aligned_probcons	0.4877	4.84E-02	5.54E-05	0.0847	0.9442
	aligned_tcoffee	0.4922	4.92E-02	5.76E-05	0.0858	0.9435
	correctaligned	0.4643	4.83E-02	7.28E-05	0.0876	0.9389
	integratedaln	0.4524	4.92E-02	9.36E-05	0.0888	0.9337
0.03	aligned_clustalw	0.5046	5.35E-02	8.09E-05	0.0926	0.9368
	aligned_mafft	0.4744	4.71E-02	5.53E-05	0.0881	0.9443
	aligned_msaprobs	0.4846	4.79E-02	5.43E-05	0.0884	0.9446
	aligned_muscle	0.492	5.00E-02	6.32E-05	0.0872	0.9417
	aligned_probalign	0.4894	4.81E-02	5.26E-05	0.0894	0.9452
	aligned_probcons	0.4873	4.69E-02	4.63E-05	0.0868	0.9474
	aligned_tcoffee	0.4942	4.71E-02	4.38E-05	0.0874	0.9484
	correctaligned	0.4587	4.99E-02	9.29E-05	0.0905	0.9338
	integratedaln	0.4472	4.82E-02	8.86E-05	0.0914	0.9348
0.04	aligned_clustalw	0.483	6.39E-02	2.79E-04	0.1068	0.9048
	aligned_mafft	0.4556	5.51E-02	1.70E-04	0.0978	0.9192
	aligned_msaprobs	0.4564	5.77E-02	2.18E-04	0.1028	0.9124
	aligned_muscle	0.4765	5.86E-02	1.86E-04	0.0977	0.9167
	aligned_probalign	0.4703	5.72E-02	1.75E-04	0.1022	0.9185
	aligned_probcons	0.4655	5.62E-02	1.68E-04	0.0989	0.9195
	aligned_tcoffee	0.4696	5.84E-02	1.99E-04	0.1021	0.915
	correctaligned	0.4398	5.71E-02	2.52E-04	0.0999	0.9081
	integratedaln	0.4187	5.59E-02	2.92E-04	0.1022	0.9035
0.05	aligned_clustalw	0.459	6.68E-02	4.68E-04	0.1228	0.8873
	aligned_mafft	0.4437	5.47E-02	1.89E-04	0.1057	0.9164
	aligned_msaprobs	0.4425	5.54E-02	2.05E-04	0.1121	0.914
	aligned_muscle	0.473	5.43E-02	1.27E-04	0.1047	0.9266
	aligned_probalign	0.4535	5.69E-02	2.07E-04	0.1141	0.9138
	aligned_probcons	0.4523	5.37E-02	1.53E-04	0.1076	0.922

	aligned_tcoffee	0.4592	5.69E-02	1.94E-04	0.1108	0.9156
	correctaligned	0.4268	5.70E-02	2.92E-04	0.1072	0.9034
	integratedaln	0.4086	5.35E-02	2.63E-04	0.1098	0.9067
0.06	aligned_clustalw	0.4581	7.16E-02	6.86E-04	0.1286	0.8722
	aligned_mafft	0.437	5.66E-02	2.47E-04	0.1142	0.9086
	aligned_msaprobs	0.4418	5.88E-02	2.88E-04	0.117	0.9038
	aligned_muscle	0.4884	5.56E-02	1.21E-04	0.1045	0.9278
	aligned_probalign	0.4446	6.01E-02	3.15E-04	0.1211	0.9011
	aligned_probcons	0.4485	5.75E-02	2.33E-04	0.1138	0.9103
	aligned_tcoffee	0.4515	6.20E-02	3.41E-04	0.1185	0.8984
	correctaligned	0.4252	5.63E-02	2.79E-04	0.1123	0.9049
	integratedaln	0.4044	5.52E-02	3.30E-04	0.1144	0.8995
0.07	aligned_clustalw	0.4409	7.53E-02	1.09E-03	0.139	0.8512
	aligned_mafft	0.4233	6.35E-02	5.50E-04	0.1234	0.8811
	aligned_msaprobs	0.4183	6.35E-02	5.90E-04	0.1281	0.8784
	aligned_muscle	0.4777	5.82E-02	1.77E-04	0.1127	0.9182
	aligned_probalign	0.4246	6.66E-02	7.00E-04	0.1328	0.8713
	aligned_probcons	0.4275	6.29E-02	4.96E-04	0.1247	0.8851
	aligned_tcoffee	0.4327	6.42E-02	5.19E-04	0.1283	0.8834
	correctaligned	0.3991	6.47E-02	8.32E-04	0.1253	0.8639
	integratedaln	0.3793	6.03E-02	7.53E-04	0.1242	0.8682
0.08	aligned_clustalw	0.435	7.33E-02	1.02E-03	0.1444	0.8543
	aligned_mafft	0.4398	6.10E-02	3.62E-04	0.119	0.8964
	aligned_msaprobs	0.4316	5.80E-02	3.04E-04	0.1267	0.9021
	aligned_muscle	0.4947	5.80E-02	1.43E-04	0.1135	0.9237
	aligned_probalign	0.4309	6.14E-02	4.17E-04	0.1321	0.8914
	aligned_probcons	0.4331	5.88E-02	3.21E-04	0.1257	0.9004
	aligned_tcoffee	0.4416	5.78E-02	2.63E-04	0.1291	0.9068
	correctaligned	0.4031	6.27E-02	6.71E-04	0.125	0.8732
	integratedaln	0.3924	5.59E-02	4.18E-04	0.1216	0.8914

BranchLength	Alignment	Slope	SE(Slope)	P(Slope>0)	Intercept	r2
0.1	aligned_clustalw	0.4271	3.29E-02	1.29E-05	0.0858	0.9656
	aligned_mafft	0.2894	2.87E-02	5.49E-05	0.086	0.9444
	aligned_msaprobs	0.327	4.07E-02	1.98E-04	0.087	0.915
	aligned_muscle	0.2825	2.82E-02	5.70E-05	0.0865	0.9437
	aligned_probalign	0.3726	3.55E-02	4.38E-05	0.0861	0.9484
	aligned_probcons	0.3268	3.59E-02	9.86E-05	0.0857	0.9325
	aligned_tcoffee	0.3725	4.02E-02	8.93E-05	0.0852	0.9347
	correctaligned	0.2727	3.13E-02	1.27E-04	0.0858	0.9267
	integratedaln	0.2656	3.11E-02	1.41E-04	0.0863	0.924
0.2	aligned_clustalw	0.7636	4.91E-02	4.48E-06	0.1666	0.9758
	aligned_mafft	0.5093	8.42E-02	9.25E-04	0.1659	0.8591
	aligned_msaprobs	0.6071	5.46E-02	3.15E-05	0.1654	0.9537
	aligned_muscle	0.5685	5.23E-02	3.60E-05	0.1646	0.9516
	aligned_probalign	0.7061	6.78E-02	4.59E-05	0.164	0.9476
	aligned_probcons	0.6021	6.19E-02	6.75E-05	0.1639	0.9405
	aligned_tcoffee	0.6739	4.89E-02	9.11E-06	0.1644	0.9693
	correctaligned	0.4964	6.20E-02	2.02E-04	0.1642	0.9145
	integratedaln	0.4562	6.51E-02	4.21E-04	0.1645	0.8911
0.3	aligned_clustalw	0.8162	8.92E-02	9.61E-05	0.2429	0.9331
	aligned_mafft	0.43	6.73E-02	6.90E-04	0.2401	0.8719
	aligned_msaprobs	0.5168	6.85E-02	2.80E-04	0.2423	0.9047
	aligned_muscle	0.62	5.88E-02	4.30E-05	0.2381	0.9487
	aligned_probalign	0.6067	8.12E-02	2.97E-04	0.2433	0.903
	aligned_probcons	0.4725	7.27E-02	6.30E-04	0.2427	0.8757
	aligned_tcoffee	0.579	7.07E-02	1.79E-04	0.2428	0.9178
	correctaligned	0.3969	5.67E-02	4.24E-04	0.2388	0.8909
	integratedaln	0.2893	5.52E-02	1.94E-03	0.2408	0.8206
0.4	aligned_clustalw	0.846	1.13E-01	2.97E-04	0.3028	0.9029

	aligned_mafft	0.5282	1.17E-01	4.09E-03	0.2941	0.7717
	aligned_msaprobs	0.4907	8.84E-02	1.44E-03	0.299	0.8372
	aligned_muscle	0.5299	4.03E-02	1.19E-05	0.2996	0.9665
	aligned_probalign	0.585	9.01E-02	6.36E-04	0.3	0.8754
	aligned_probcons	0.5427	7.56E-02	3.70E-04	0.2973	0.8956
	aligned_tcoffee	0.5755	9.60E-02	9.71E-04	0.3	0.8568
	correctaligned	0.4323	1.02E-01	5.35E-03	0.2933	0.7511
	integratedaln	0.2458	6.79E-02	1.11E-02	0.2957	0.6859
0.5	aligned_clustalw	0.8567	7.68E-02	3.11E-05	0.3483	0.9539
	aligned_mafft	0.4829	6.94E-02	4.39E-04	0.3379	0.8896
	aligned_msaprobs	0.4794	4.89E-02	6.49E-05	0.3424	0.9412
	aligned_muscle	0.7386	7.79E-02	7.82E-05	0.3386	0.9375
	aligned_probalign	0.5631	5.16E-02	3.53E-05	0.3436	0.952
	aligned_probcons	0.4999	3.92E-02	1.42E-05	0.3413	0.9644
	aligned_tcoffee	0.5512	5.06E-02	3.54E-05	0.3453	0.9519
	correctaligned	0.3511	6.41E-02	1.55E-03	0.3371	0.8334
	integratedaln	0.1962	5.49E-02	1.17E-02	0.3352	0.6807
0.6	aligned_clustalw	0.4164	8.01E-02	2.02E-03	0.3961	0.8183
	aligned_mafft	0.2517	5.63E-02	4.24E-03	0.3797	0.769
	aligned_msaprobs	0.1785	5.81E-02	2.20E-02	0.3863	0.6109
	aligned_muscle	0.4879	5.89E-02	1.67E-04	0.3848	0.9196
	aligned_probalign	0.2488	5.85E-02	5.36E-03	0.3893	0.751
	aligned_probcons	0.1417	6.27E-02	6.45E-02	0.3881	0.46
	aligned_tcoffee	0.2664	3.89E-02	4.77E-04	0.3901	0.8866
	correctaligned	0.0923	6.26E-02	1.91E-01	0.3802	0.266
	integratedaln	-0.0495	5.64E-02	4.14E-01	0.3748	0.1138
0.7	aligned_clustalw	0.0237	4.89E-02	6.46E-01	0.4347	0.0376
	aligned_mafft	-0.1107	9.98E-02	3.10E-01	0.4162	0.1701
	aligned_msaprobs	-0.191	8.84E-02	7.41E-02	0.4261	0.4375
	aligned_muscle	0.3887	8.70E-02	4.24E-03	0.4146	0.7691
	aligned_probalign	-0.1379	4.69E-02	2.59E-02	0.4302	0.5905
	aligned_probcons	-0.1813	5.41E-02	1.54E-02	0.4265	0.6521
	aligned_tcoffee	-0.0925	6.88E-02	2.28E-01	0.4278	0.2314
	correctaligned	-0.3151	7.82E-02	6.86E-03	0.415	0.7304
	integratedaln	-0.403	7.76E-02	2.03E-03	0.4103	0.8179
0.8	aligned_clustalw	-0.1818	7.08E-02	4.25E-02	0.4481	0.5234
	aligned_mafft	-0.1796	4.45E-02	6.79E-03	0.431	0.7313
	aligned_msaprobs	-0.1835	9.13E-02	9.11E-02	0.435	0.4025
	aligned_muscle	0.3887	9.04E-02	5.10E-03	0.4239	0.7549
	aligned_probalign	-0.2145	8.54E-02	4.57E-02	0.443	0.5127
	aligned_probcons	-0.1157	8.34E-02	2.15E-01	0.4345	0.2429
	aligned_tcoffee	-0.0704	1.04E-01	5.23E-01	0.4382	0.0712
	correctaligned	-0.3271	6.47E-02	2.32E-03	0.4226	0.8099
	integratedaln	-0.3873	9.44E-02	6.34E-03	0.4163	0.7372

Table S14. Ancestral sequence reconstruction error rates were positively correlated with increasing branch lengths and weakly correlated with increasing insertion-deletion rates in 3-taxon simulations. We simulated protein sequences along a 3-taxon phylogeny with equal branch lengths and the same insertion-deletion (indel) rate across the phylogeny (see Methods, Supplementary Fig. S12). The resulting extant sequence data was aligned using 7 different sequence-alignment methods (see Methods). We used each alignment to infer the most likely ancestral sequence at the single node on the 3-taxon phylogeny. Additionally, alignment-integrated ancestral sequences were generated by combining inferences from the 7 sequence-alignment methods (see Methods). In each case, we compared the inferred ancestral sequence to the correct simulated sequence to estimate ancestral sequence reconstruction (ASR) error rates (see Methods). Sequence-wide error rates (errors/site) were computed by dividing the total number of errors by the length of the pairwise alignment of the inferred and correct

ancestral sequences. Top table reports results from linear-regression of indel rate vs ASR error rate, for each branch length and alignment method. Bottom table reports results from linear-regression of branch length vs ASR error rate, for each indel rate and alignment method. In each case, we report the slope (and standard error) of the best-fit regression line, the *p*-value obtained by testing if the slope is significantly different from zero, the intercept of the best-fit regression line, and the correlation (r^2) between the predictive (branch length or indel rate) and response (ASR error rate) variable.

Supplementary Figures

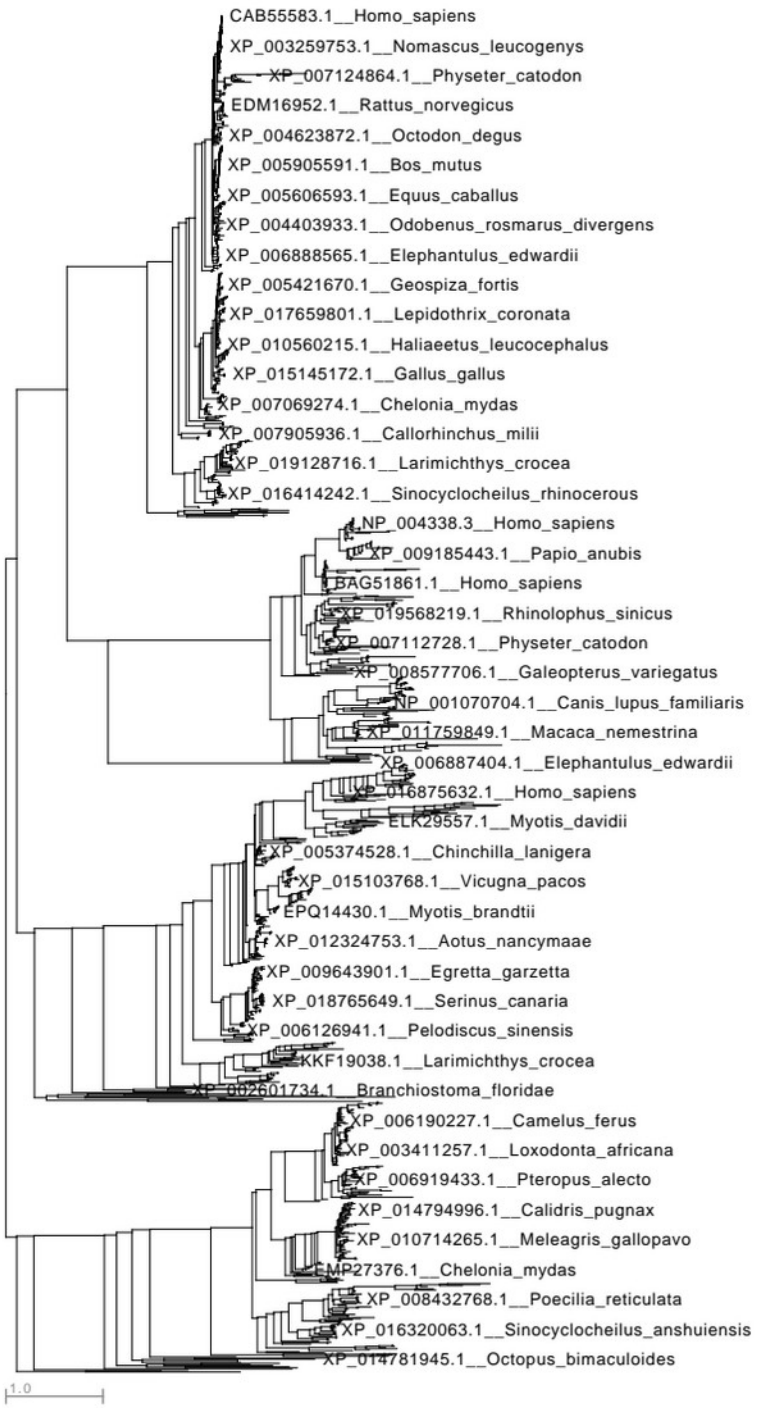

Figure S1 Part 1, CARD domain. Empirical protein domain family phylogenies used to simulate sequence data. We simulated protein sequence data along the indicated empirical phylogenies. Branch lengths are scaled to the expected number of substitutions/site. Part 1 shows the empirical phylogeny of the CARD domain family; other domain family phylogenies are shown in parts 2-5, below.

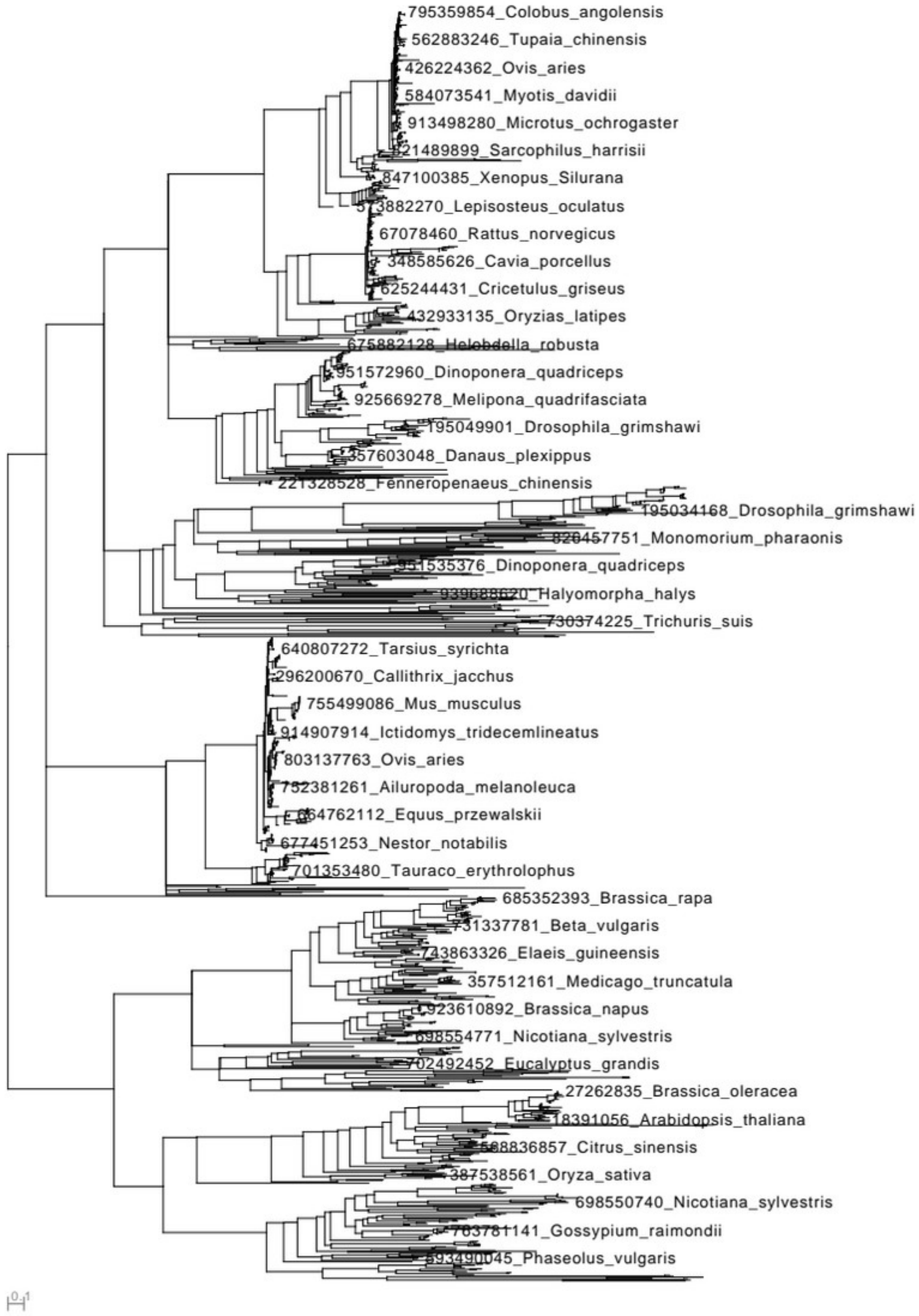

Figure S1 Part 2, DSRM1 domain.

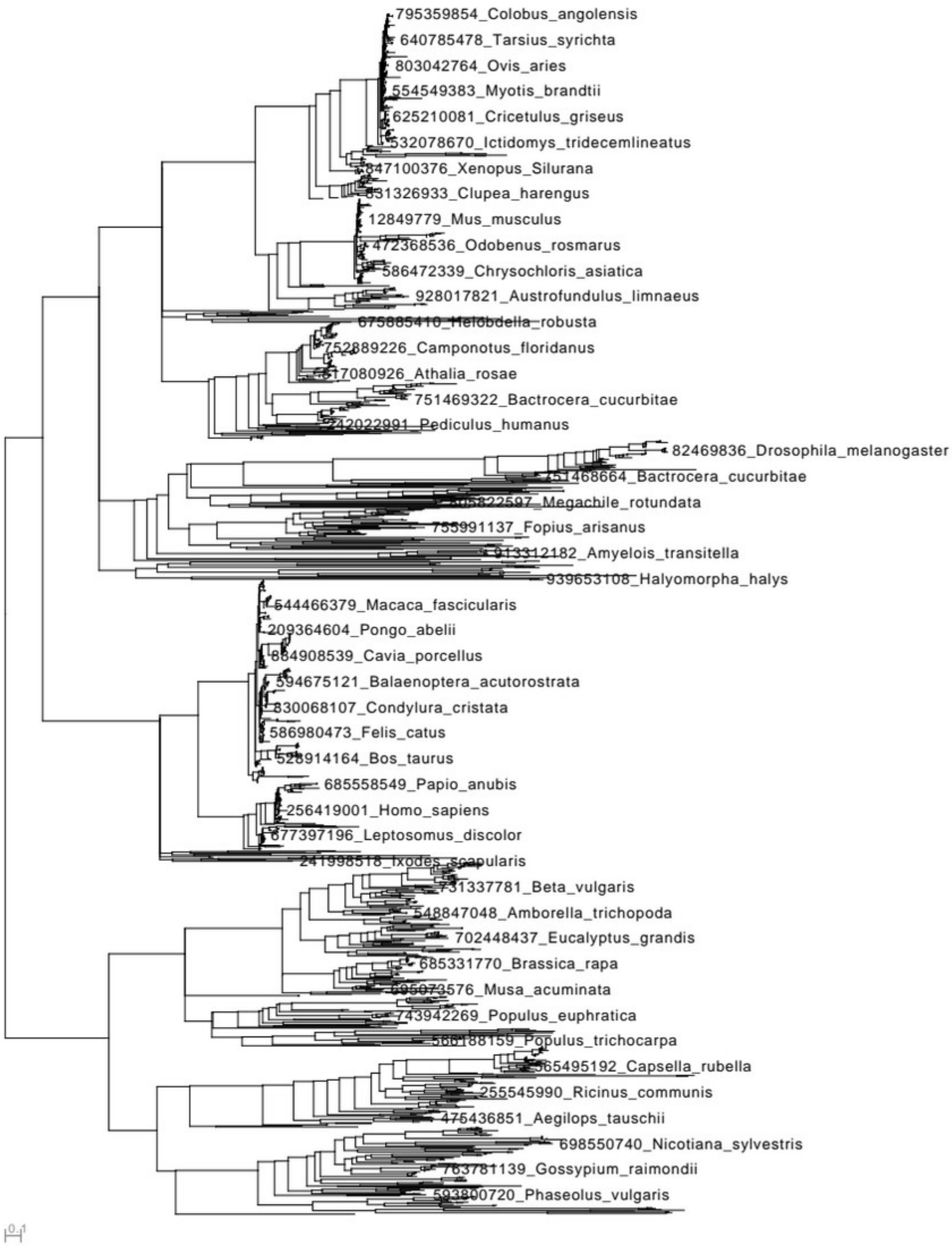

Figure S1 Part 3, DSRM2 domain.

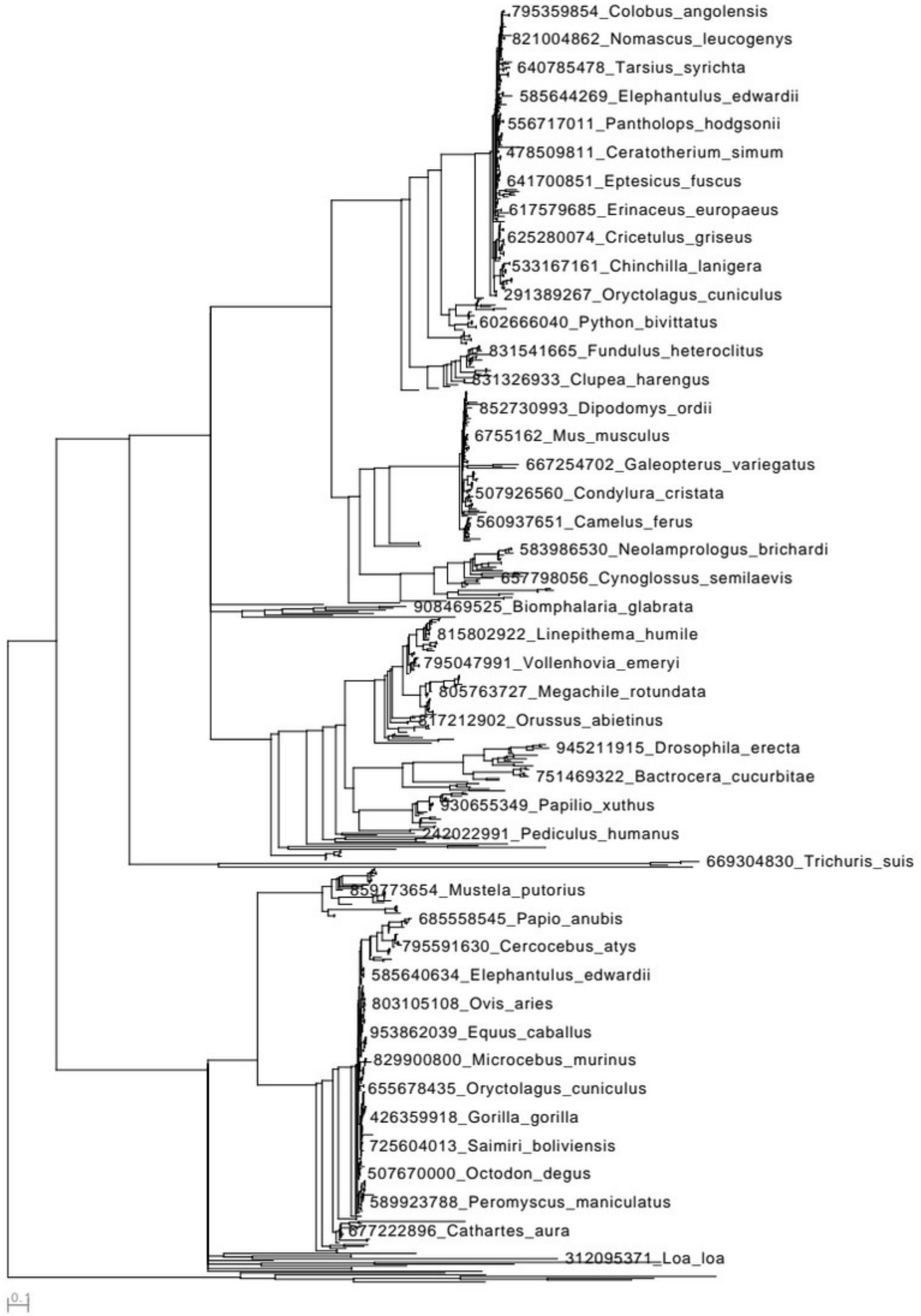

Figure S1 Part 4, DSRM3 domain.

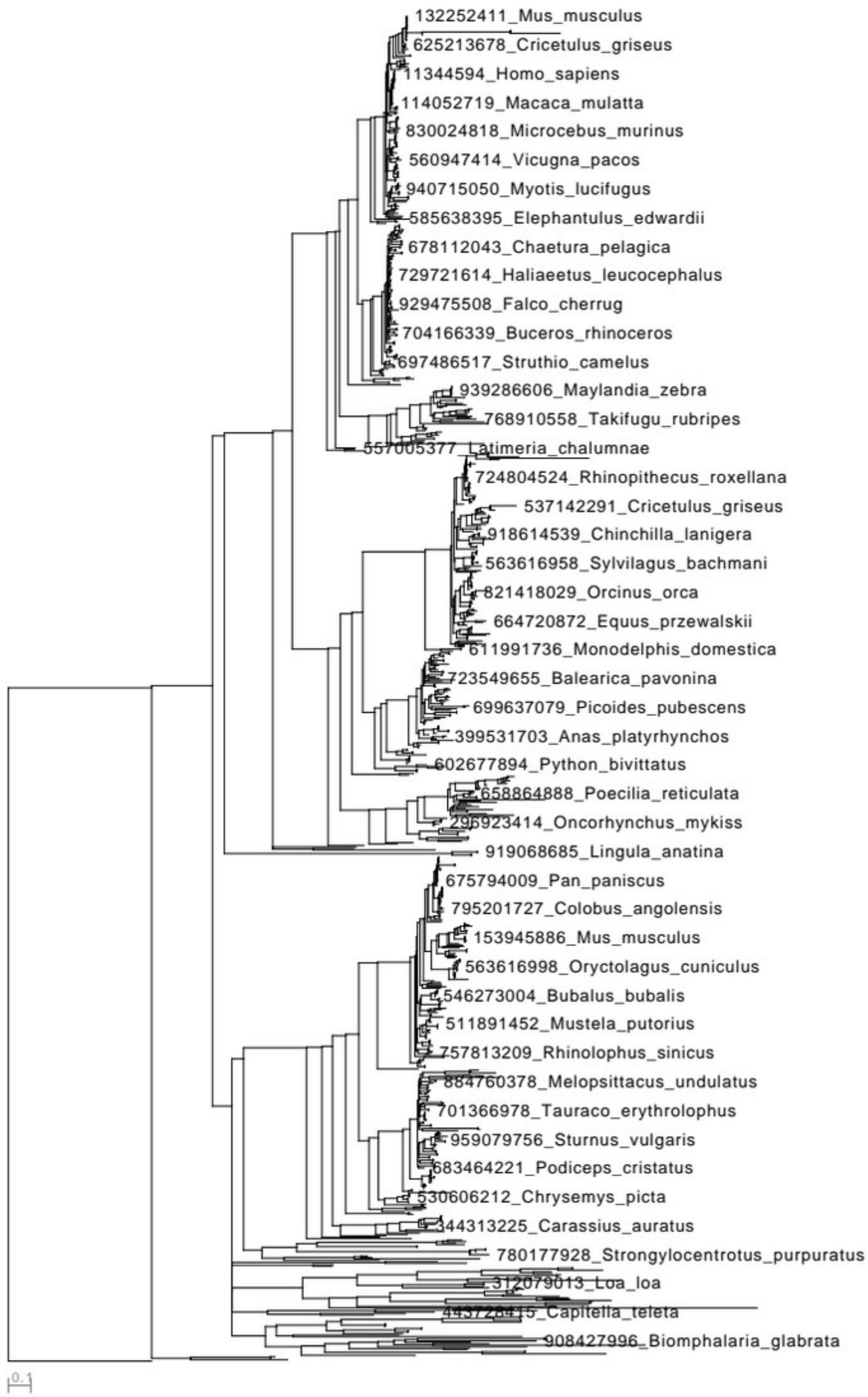

Figure S1 Part 5, RD domain.

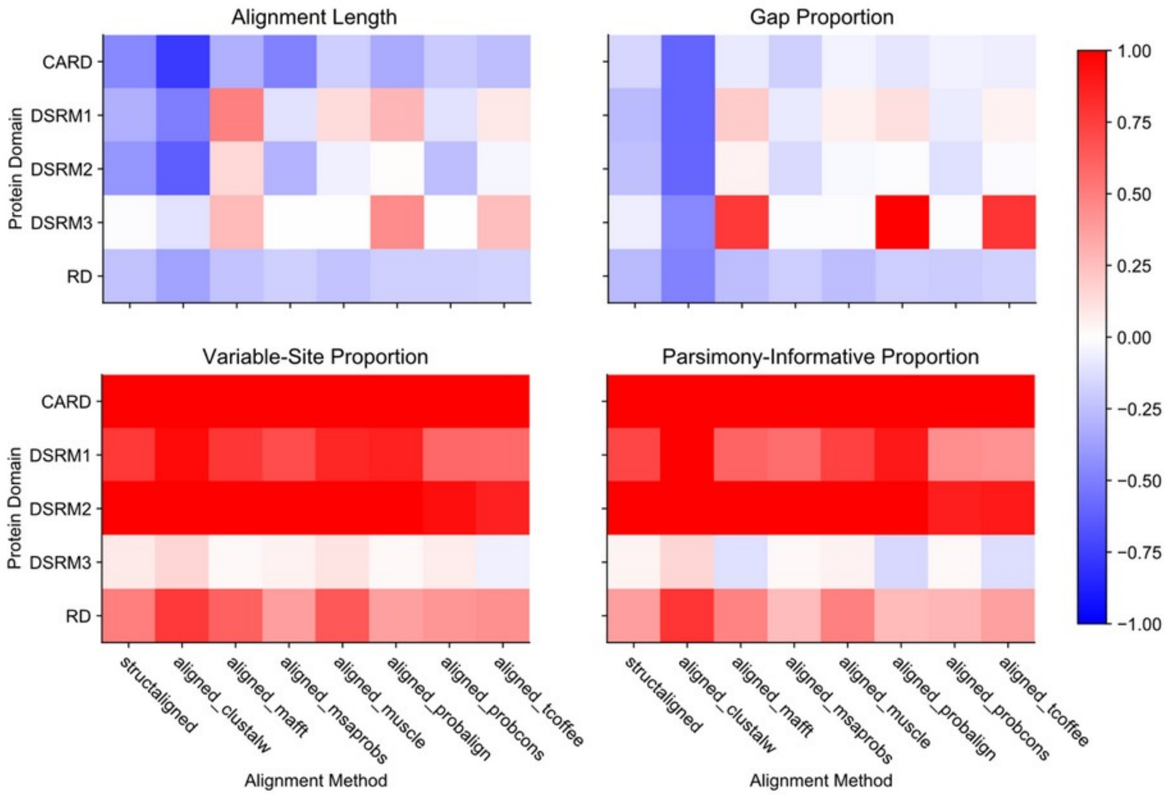

Figure S2. Structure-guided and sequence-alignment methods typically underestimate correct alignment lengths and gap proportions while overestimating the proportions of variable and parsimony-informative sites. We simulated alignments of 5 protein domain families, using empirically-derived simulation conditions, and aligned the resulting replicate sequence data using structure-guided and 7 different sequence-alignment methods (see Methods). Colors indicate the proportion of the correct-alignment's value by which the inferred value deviates from the correct-alignment's value, with negative numbers (blue) indicating underestimation of the correct-alignment's value, and positive numbers (red) indicating overestimation of the correct-alignment's value. A value of 1 (-1) indicates over- (under-) estimation of the correct value by 50%.

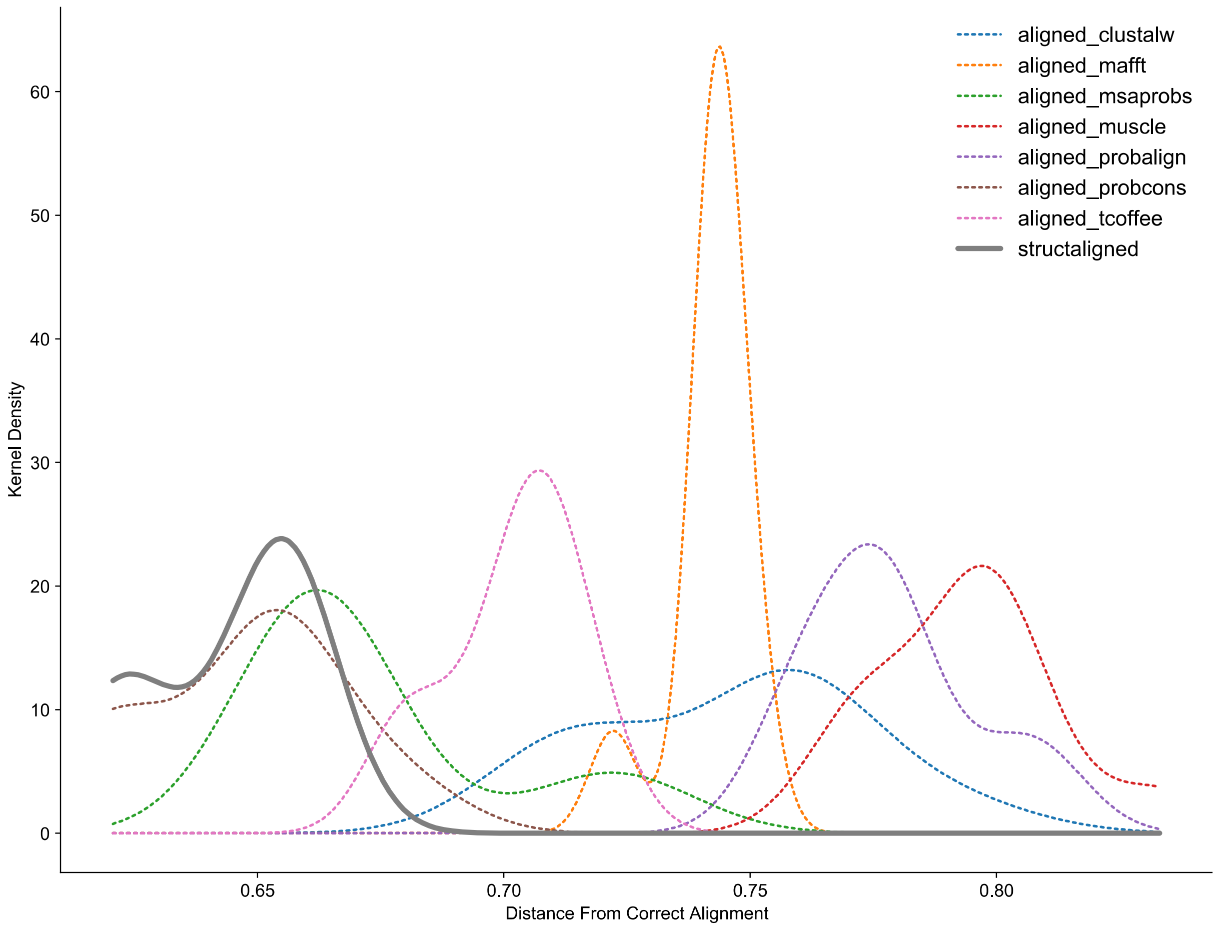

Figure S3 Part 1, CARD Domain. Alignment distance distributions differ across alignment methods and protein domain families. We simulated alignments of 5 protein domain families, using empirically-derived simulation conditions, and aligned the resulting replicate sequence data using structure-guided and 7 different sequence-alignment methods (see Methods). For each sequence alignment, we measured the position-wise distance of that alignment to the correct simulated alignment, which estimates the probability of randomly selecting an incorrectly-aligned residue from the alignment (see Methods). We estimated the distribution of alignment distances across replicate simulations using kernel density estimation. Part 1 shows results for the CARD domain family; results for other domain families are shown in parts 2-5, below.

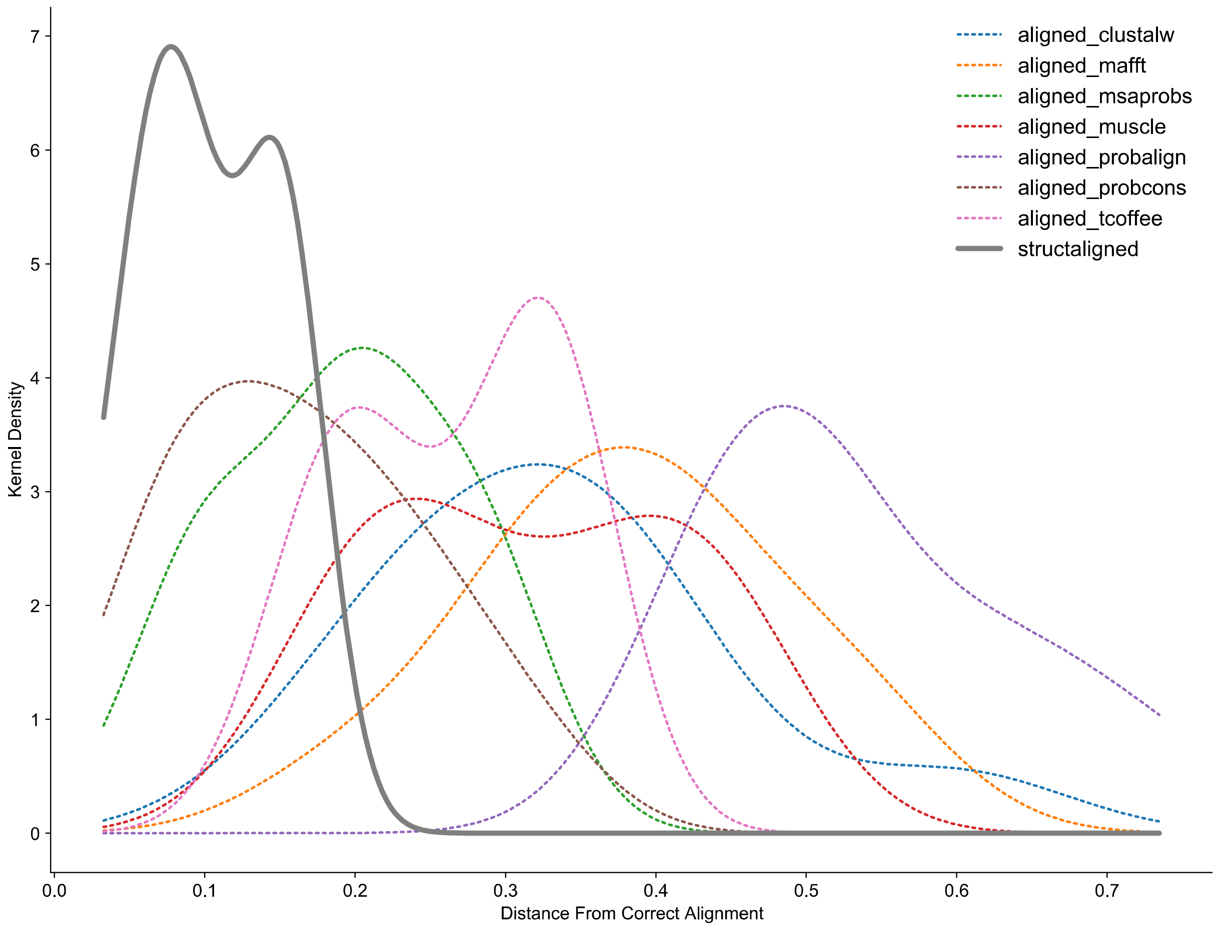

Figure S3 Part 2, DSRM1 Domain.

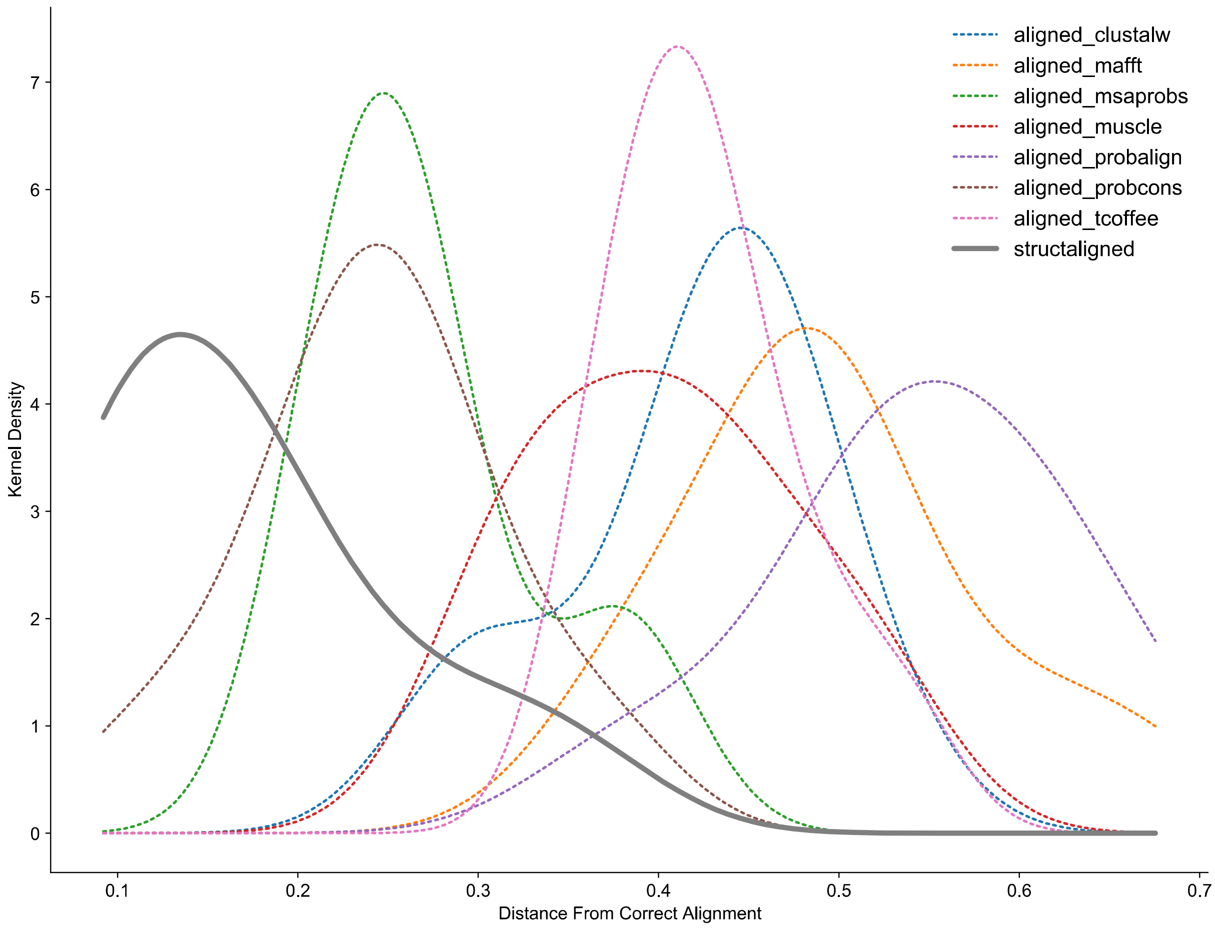

Figure S3 Part 3, DSRM2 Domain.

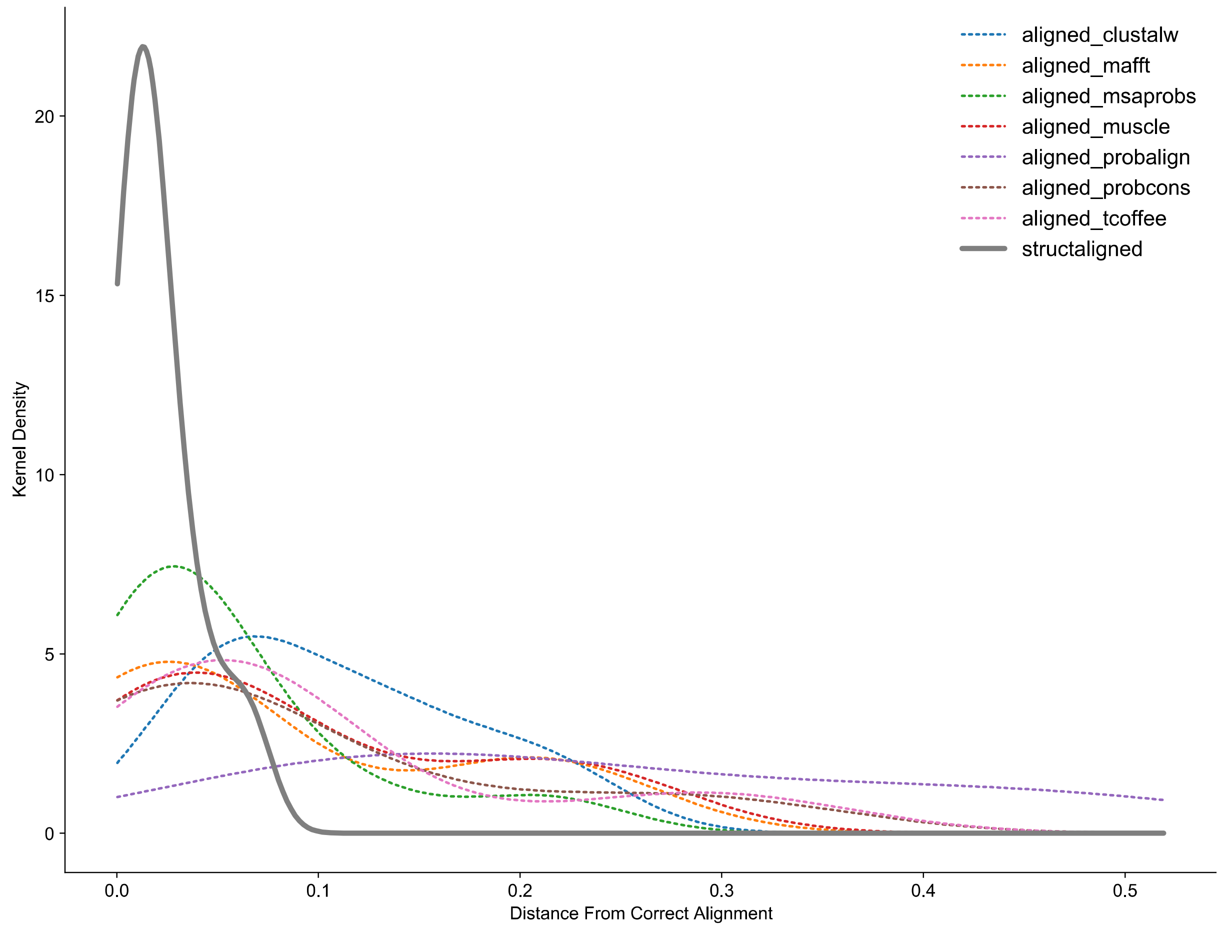

Figure S3 Part 4, DSRM3 Domain.

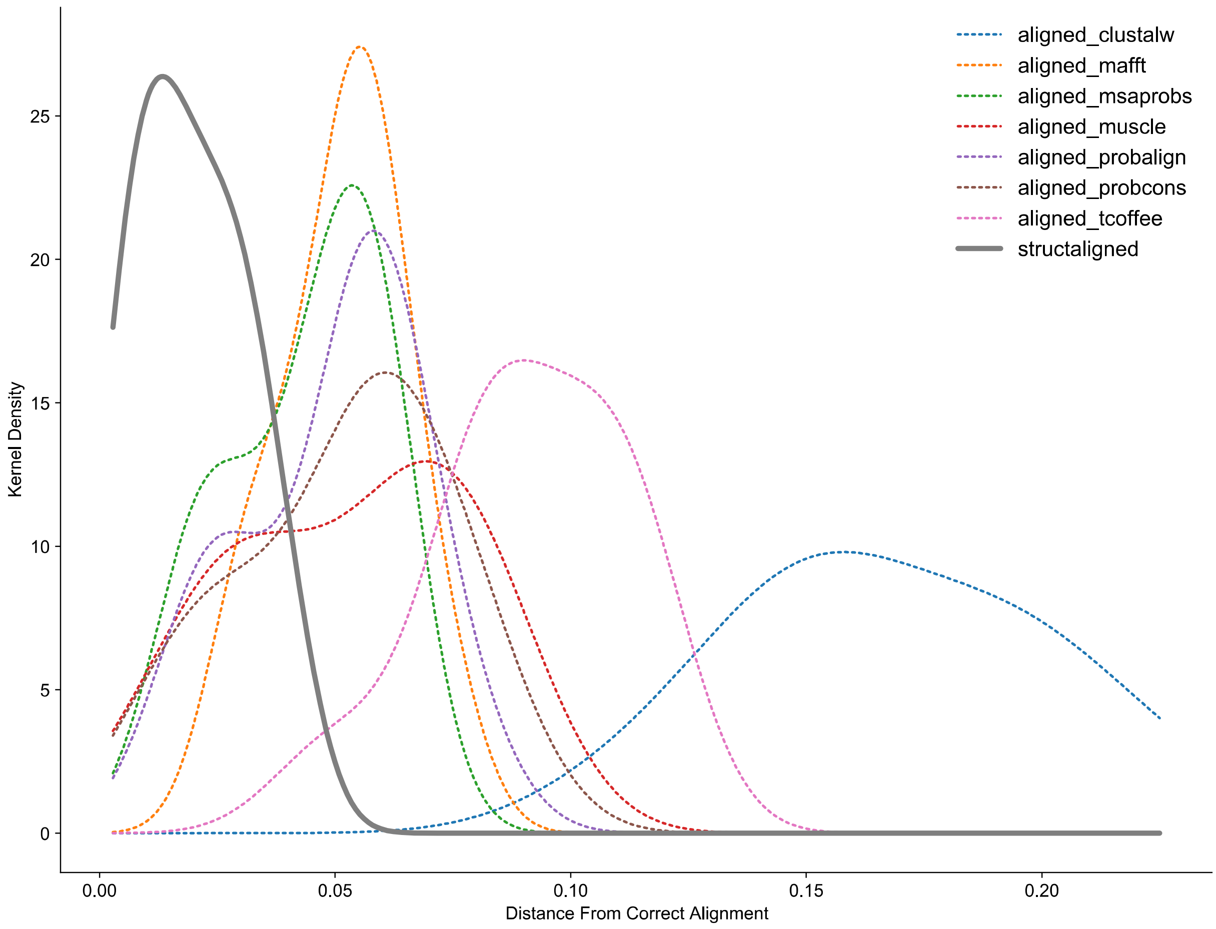

Figure S3 Part 5, RD Domain.

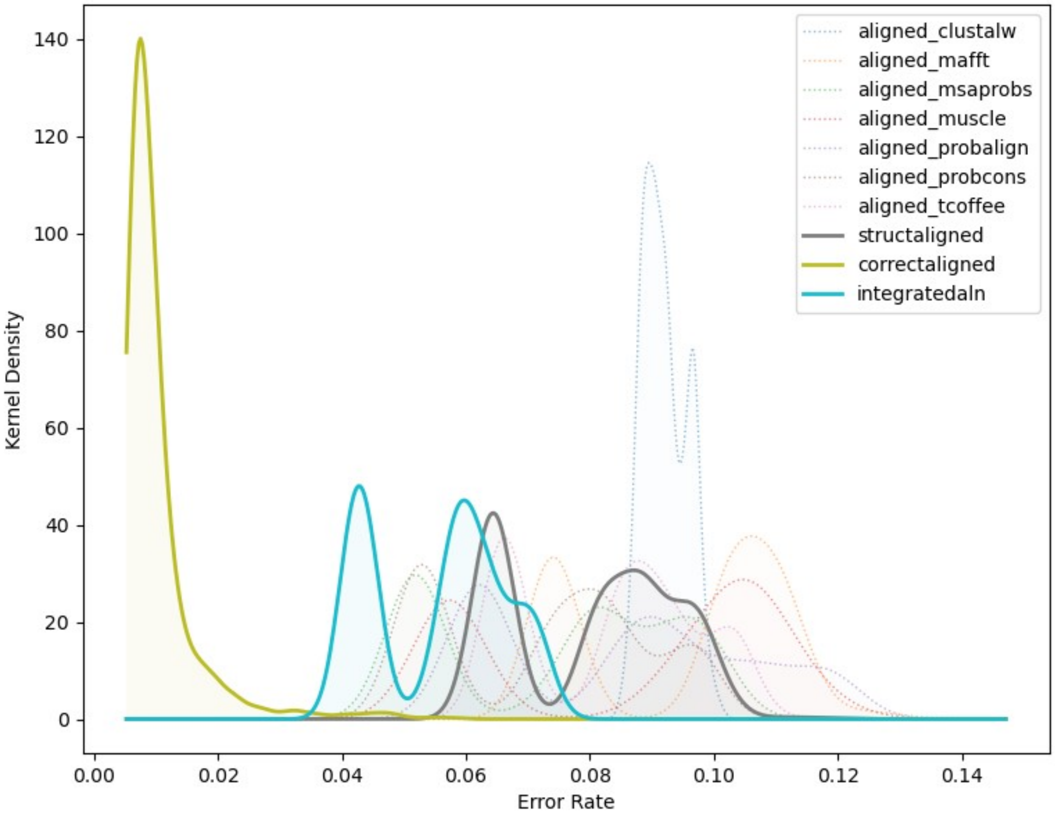

Figure S4 Part 1, CARD Domain. Total ancestral sequence reconstruction (ASR) error rates are low when the correct alignment is known in advance, higher when the alignment is inferred using sequence-alignment methods, and intermediate when structure-guided alignments or alignment-integrated ASR is used. We simulated extant and ancestral sequences for 5 protein domain families, using empirically-derived conditions, and aligned the resulting extant sequence data using structure-guided and 7 different sequence-alignment methods (see Methods). We used each alignment to infer the most likely ancestral sequence at each node on the phylogeny. Additionally, alignment-integrated ancestral sequences were generated by combining inferences from the 7 sequence-alignment methods (see Methods). In each case, we compared the inferred ancestral sequences to the correct simulated sequence to estimate ancestral sequence reconstruction (ASR) error rates (see Methods). ASR errors were divided into 4 “errorType” categories: 1) residue errors, in which both correct and inferred ancestral sequences have a residue at a given position in the alignment, but the residues differ; 2) insertion errors, in which the inferred sequence has a residue at a given alignment position, but the correct sequence has a gap state; 3) deletion errors, in which the inferred sequence has a gap, but the correct sequence has a residue, and 4) total errors, which combine residue- insertion- and deletion-errors. Sequence-wide error rates (errors/site) were computed by dividing the number of errors by the length of the pairwise alignment of the inferred and correct ancestral sequences. Error rate distributions were inferred by kernel density estimation. Here we report the total ASR-error distributions. Part 1 shows results for the CARD domain family; results for other domains are shown in parts 2-5 below.

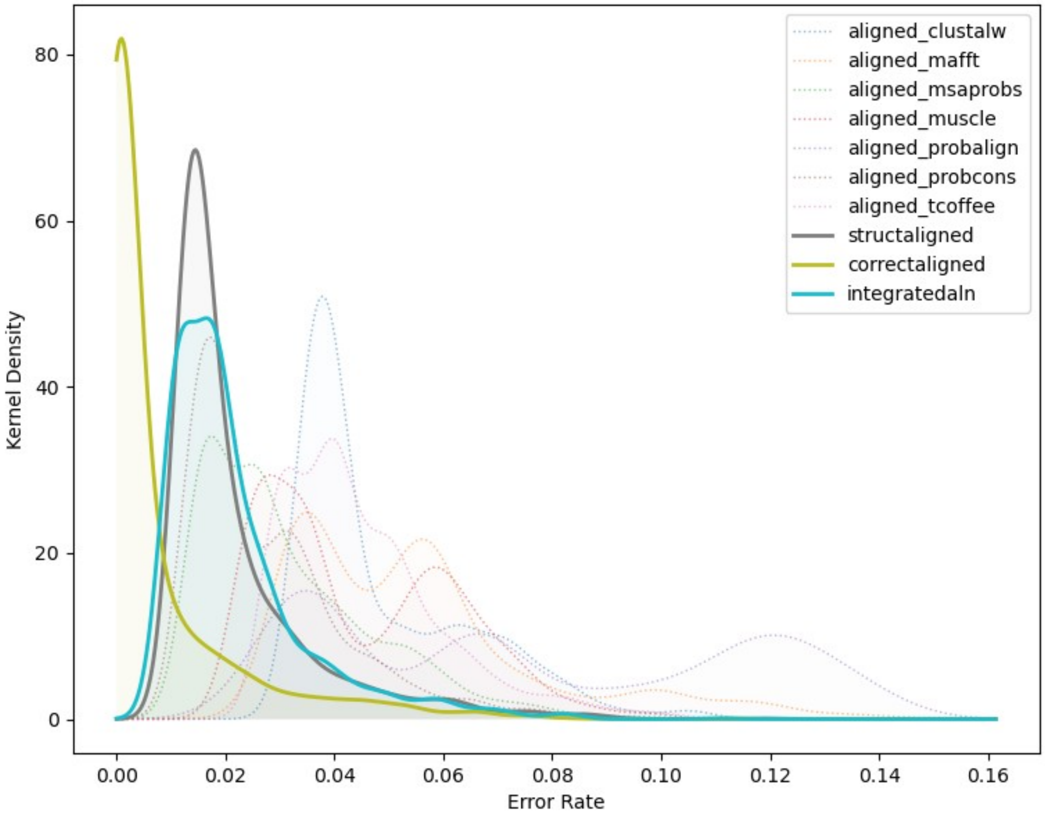

Figure S4 Part 2, DSRM1 Domain.

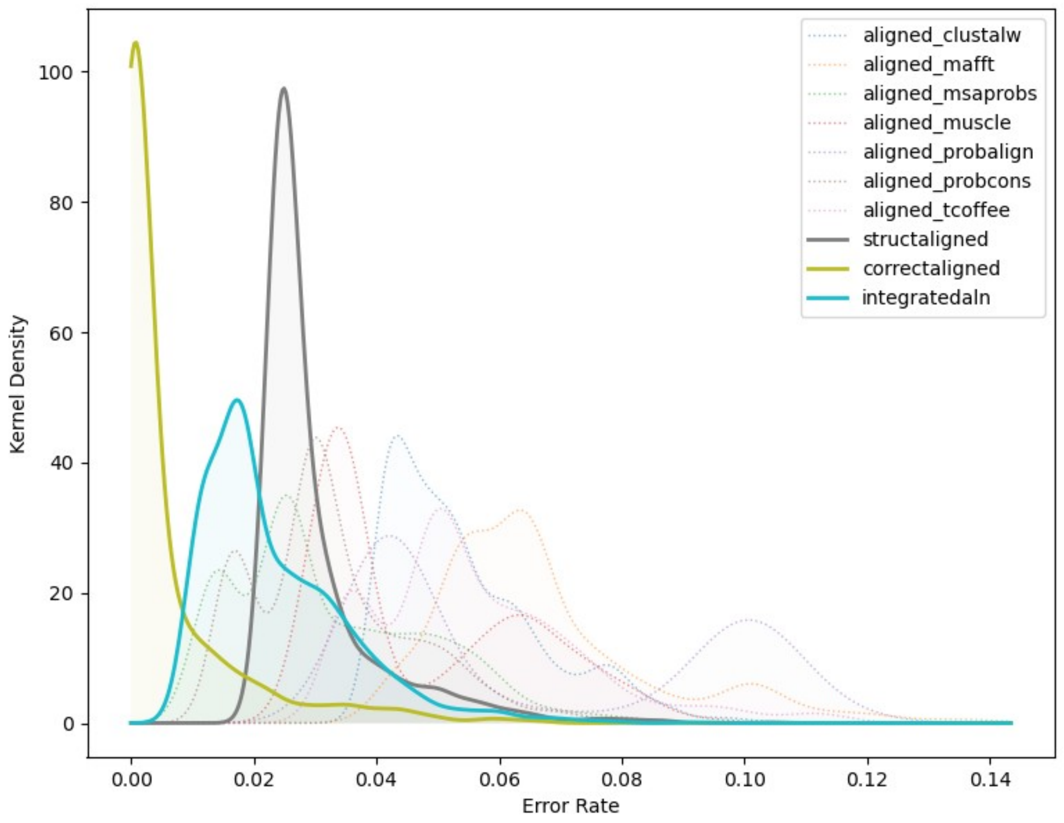

Figure S4 Part 3, DSRM2 Domain.

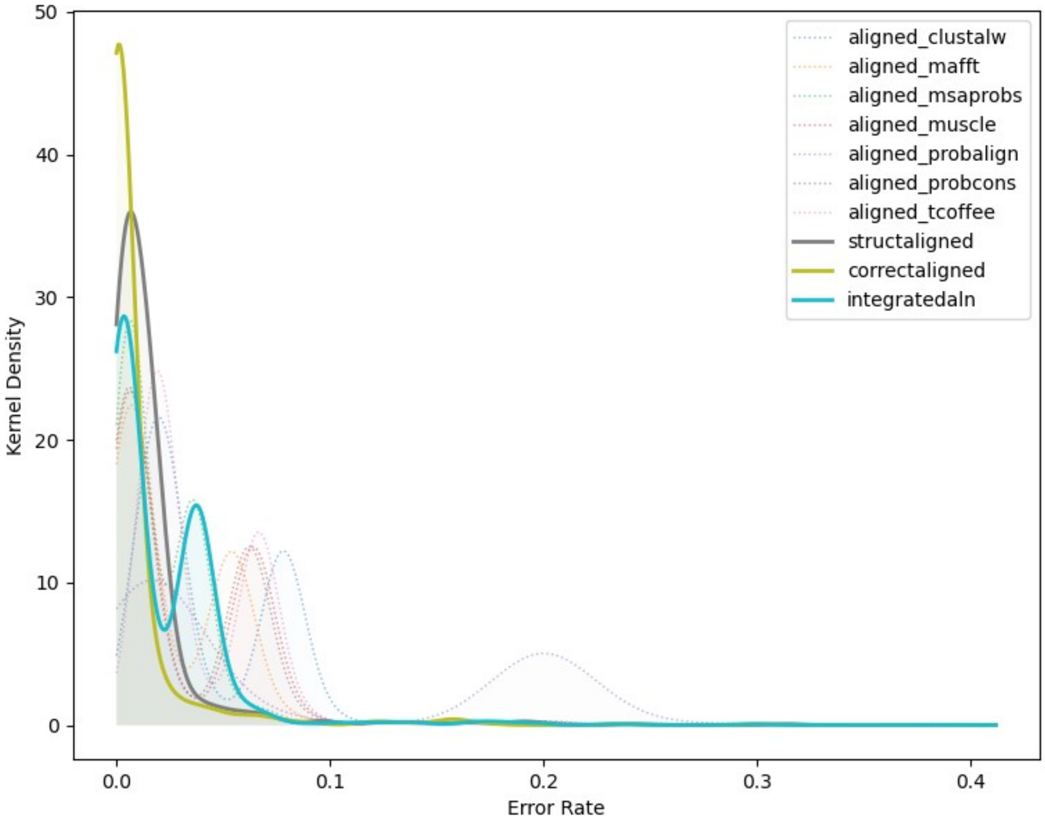

Figure S4 Part 4, DSRM3 Domain.

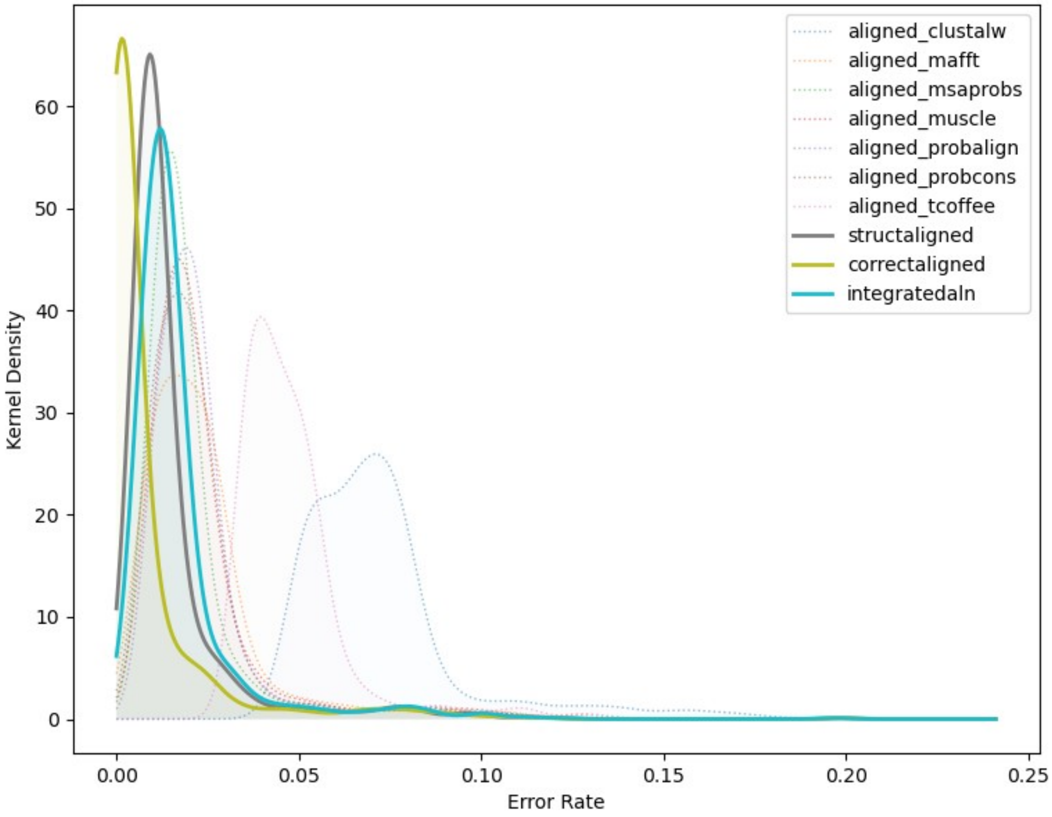

Figure S4 Part 5, RD Domain.

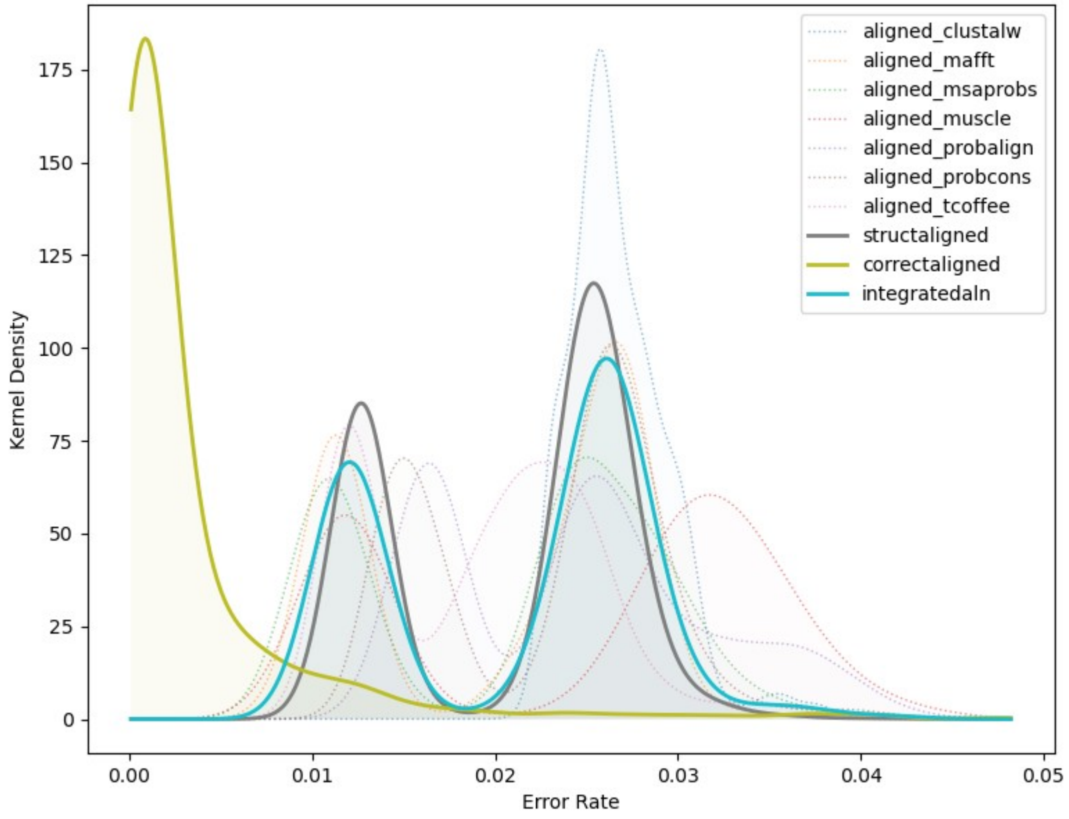

Figure S5 Part 1, CARD Domain. Ancestral sequence reconstruction (ASR) residue-reconstruction error rates are low when the correct alignment is known in advance, higher when the alignment is inferred using sequence-alignment methods, and intermediate when structure-guided alignments or alignment-integrated ASR is used. We simulated extant and ancestral sequences for 5 protein domain families, using empirically-derived conditions, and aligned the resulting extant sequence data using structure-guided and 7 different sequence-alignment methods (see Methods). We used each alignment to infer the most likely ancestral sequence at each node on the phylogeny. Additionally, alignment-integrated ancestral sequences were generated by combining inferences from the 7 sequence-alignment methods (see Methods). In each case, we compared the inferred ancestral sequences to the correct simulated sequence to estimate ancestral sequence reconstruction (ASR) error rates (see Methods). ASR errors were divided into 4 “errorType” categories: 1) residue errors, in which both correct and inferred ancestral sequences have a residue at a given position in the alignment, but the residues differ; 2) insertion errors, in which the inferred sequence has a residue at a given alignment position, but the correct sequence has a gap state; 3) deletion errors, in which the inferred sequence has a gap, but the correct sequence has a residue, and 4) total errors, which combine residue- insertion- and deletion-errors. Sequence-wide error rates (errors/site) were computed by dividing the number of errors by the length of the pairwise alignment of the inferred and correct ancestral sequences. Error rate distributions were inferred by kernel density estimation. Here we report the ASR-error distributions for residue errors. Part 1 shows results for the CARD domain family; results for other domains are shown in parts 2-5 below.

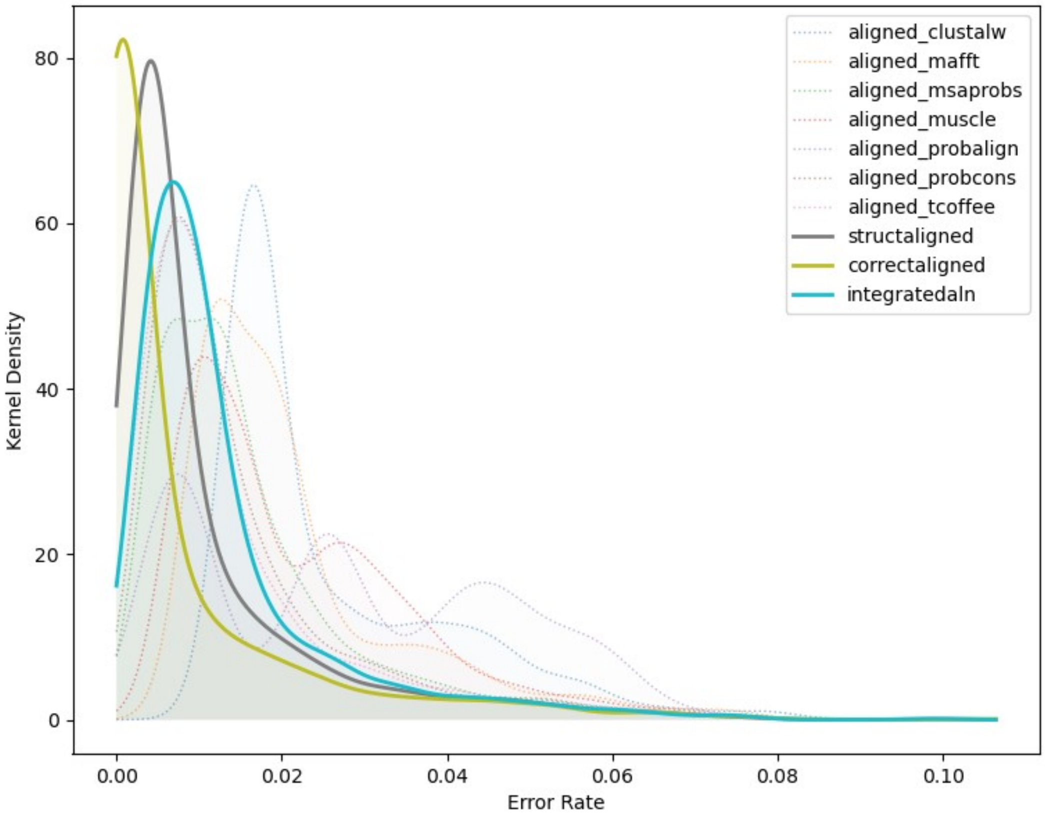

Figure S5 Part 2, DSRM1 Domain.

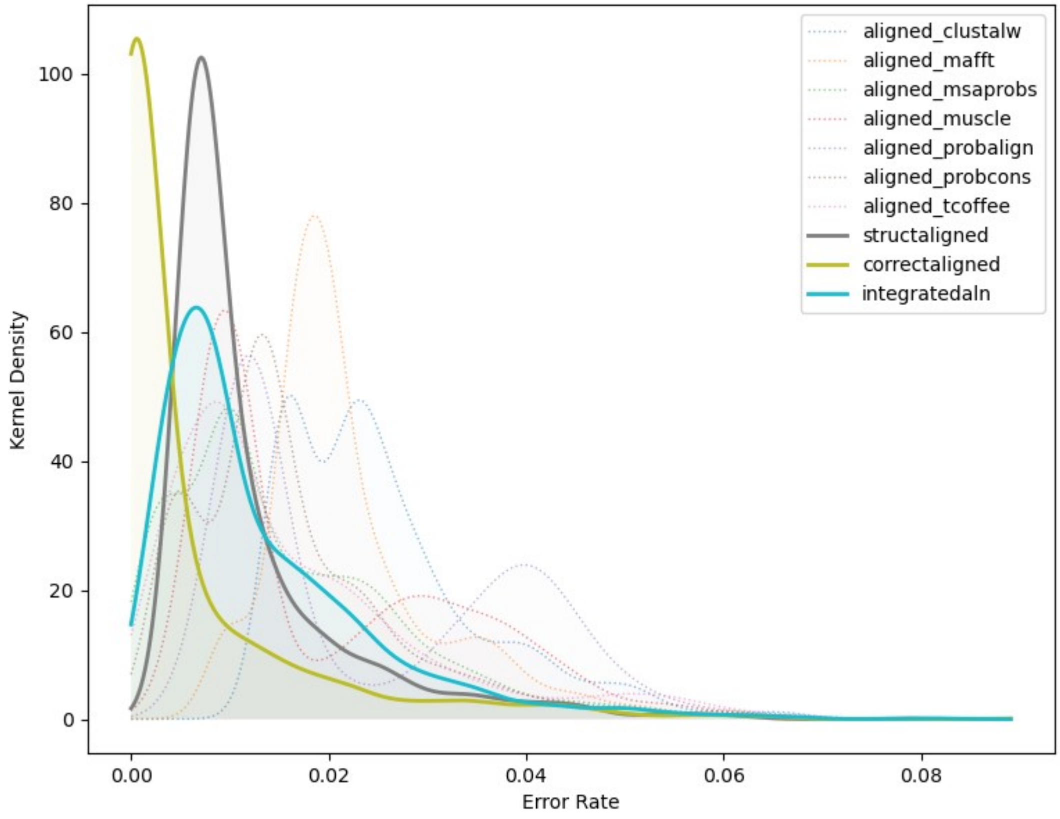

Figure S5 Part 3, DSRM2 Domain.

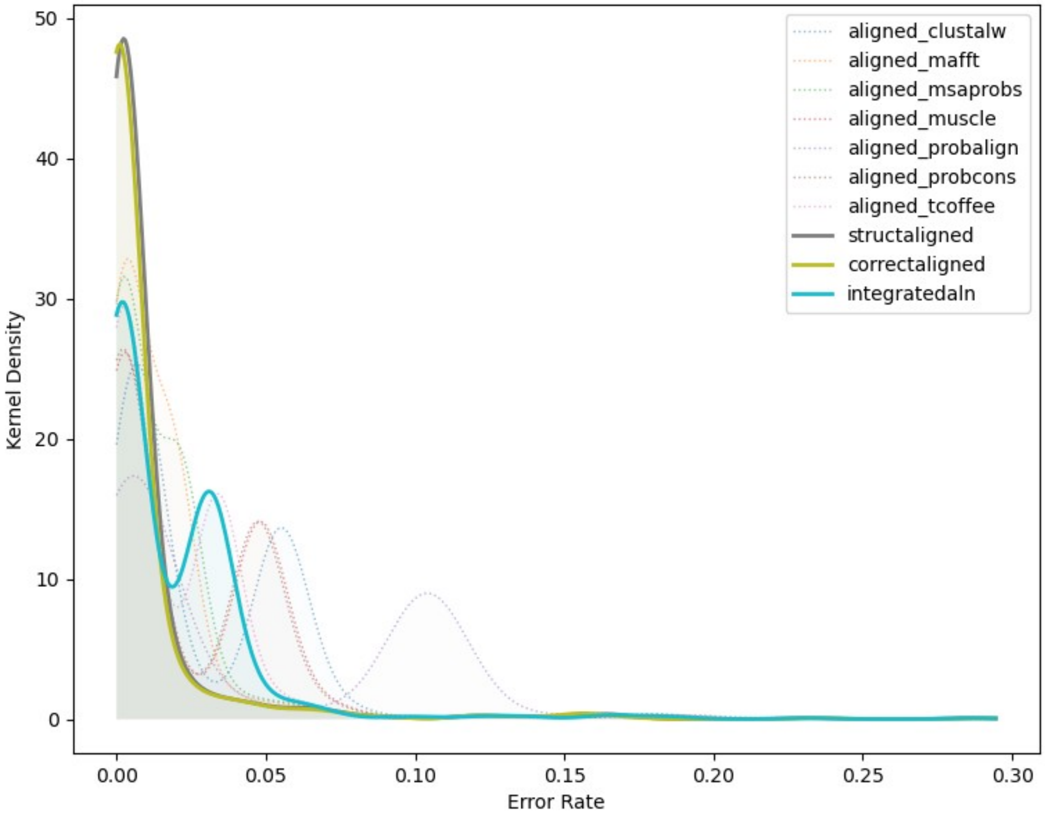

Figure S5 Part 4, DSRM3 Domain.

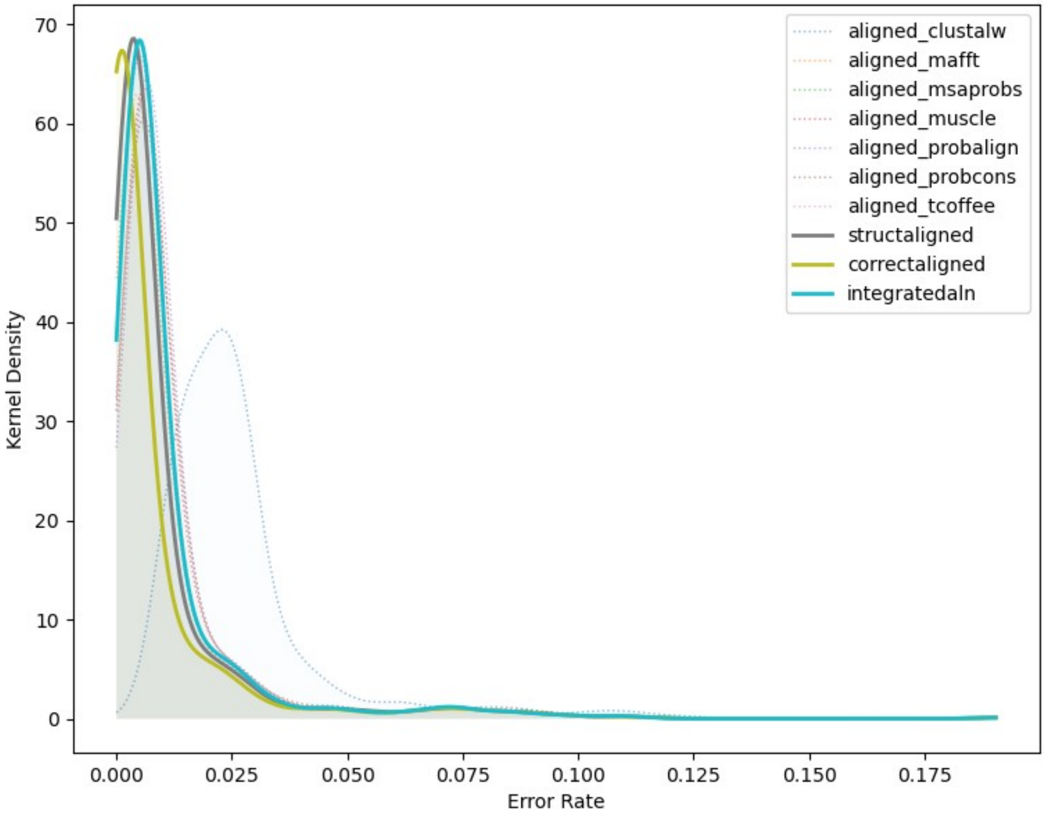

Figure S5 Part 5, RD Domain.

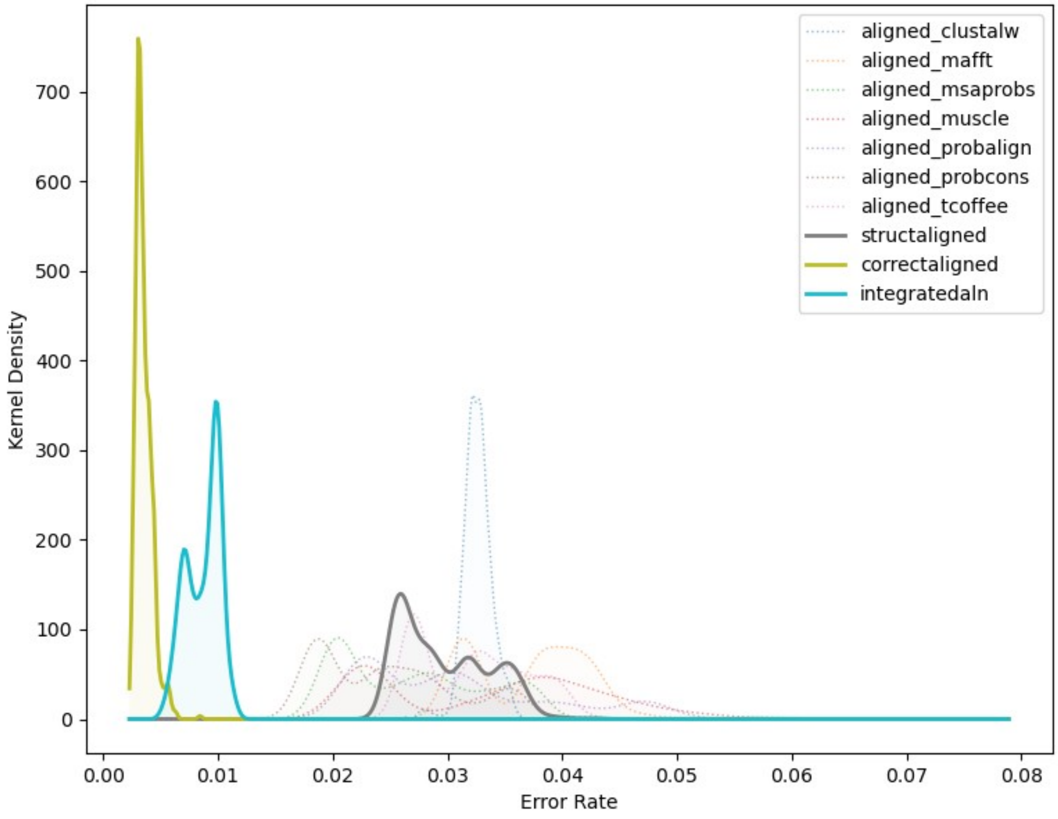

Figure S6 Part 1, CARD Domain. Ancestral sequence reconstruction (ASR) insertion error rates are low when the correct alignment is known in advance, higher when the alignment is inferred using sequence-alignment methods, and intermediate when structure-guided alignments or alignment-integrated ASR is used. We simulated extant and ancestral sequences for 5 protein domain families, using empirically-derived conditions, and aligned the resulting extant sequence data using structure-guided and 7 different sequence-alignment methods (see Methods). We used each alignment to infer the most likely ancestral sequence at each node on the phylogeny. Additionally, alignment-integrated ancestral sequences were generated by combining inferences from the 7 sequence-alignment methods (see Methods). In each case, we compared the inferred ancestral sequences to the correct simulated sequence to estimate ancestral sequence reconstruction (ASR) error rates (see Methods). ASR errors were divided into 4 “errorType” categories: 1) residue errors, in which both correct and inferred ancestral sequences have a residue at a given position in the alignment, but the residues differ; 2) insertion errors, in which the inferred sequence has a residue at a given alignment position, but the correct sequence has a gap state; 3) deletion errors, in which the inferred sequence has a gap, but the correct sequence has a residue, and 4) total errors, which combine residue- insertion- and deletion-errors. Sequence-wide error rates (errors/site) were computed by dividing the number of errors by the length of the pairwise alignment of the inferred and correct ancestral sequences. Error rate distributions were inferred by kernel density estimation. Here we report the ASR-error distributions for insertion errors. Part 1 shows results for the CARD domain family; results for other domains are shown in parts 2-5 below.

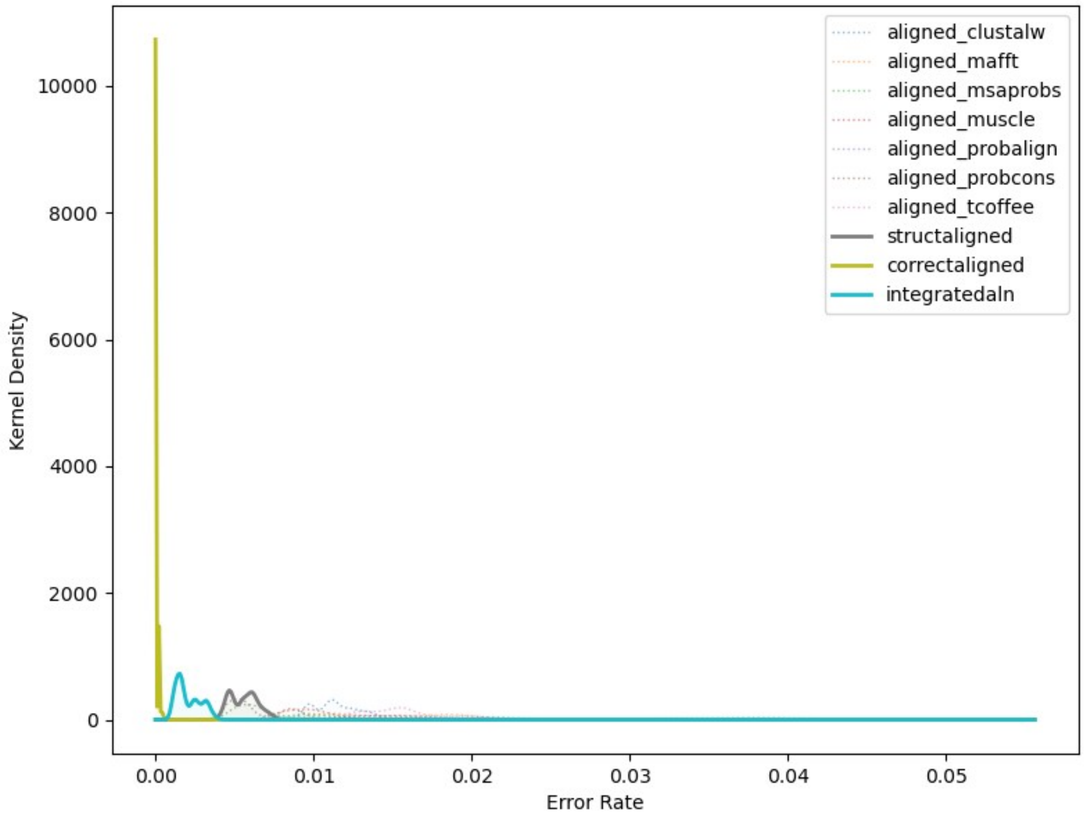

Figure S6 Part 2, DSRM1 Domain.

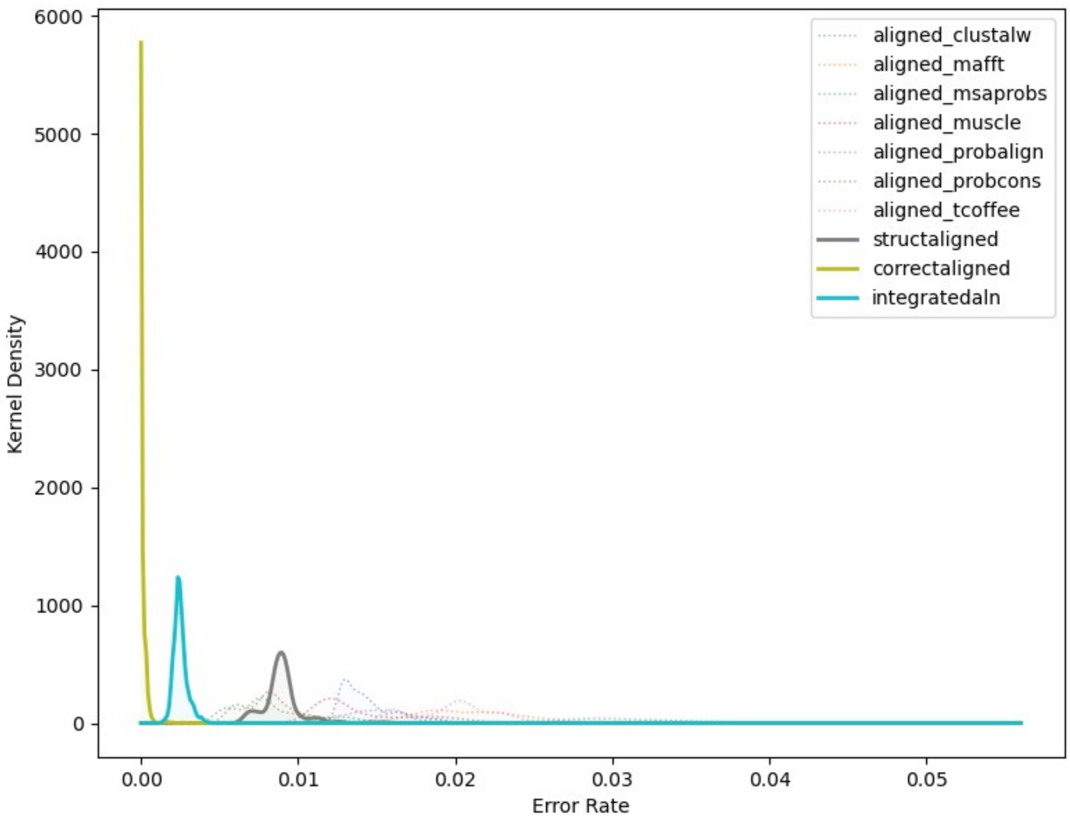

Figure S6 Part 3, DSRM2 Domain.

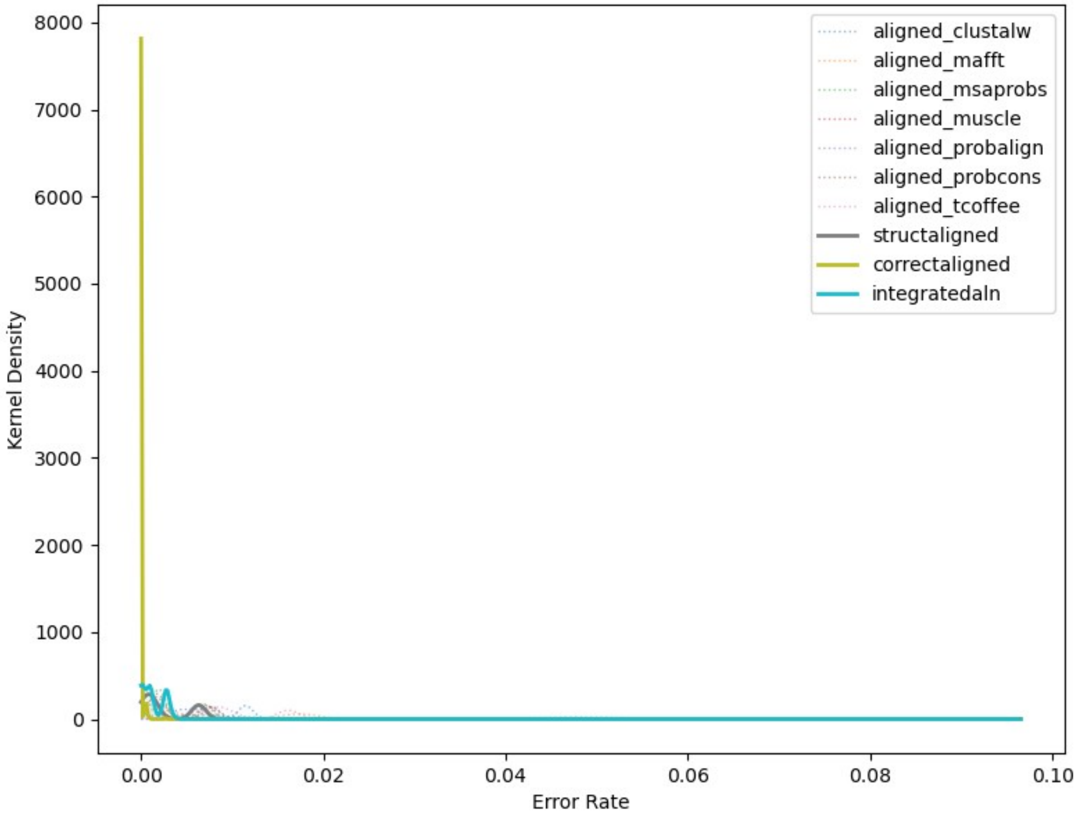

Figure S6 Part 4, DSRM3 Domain.

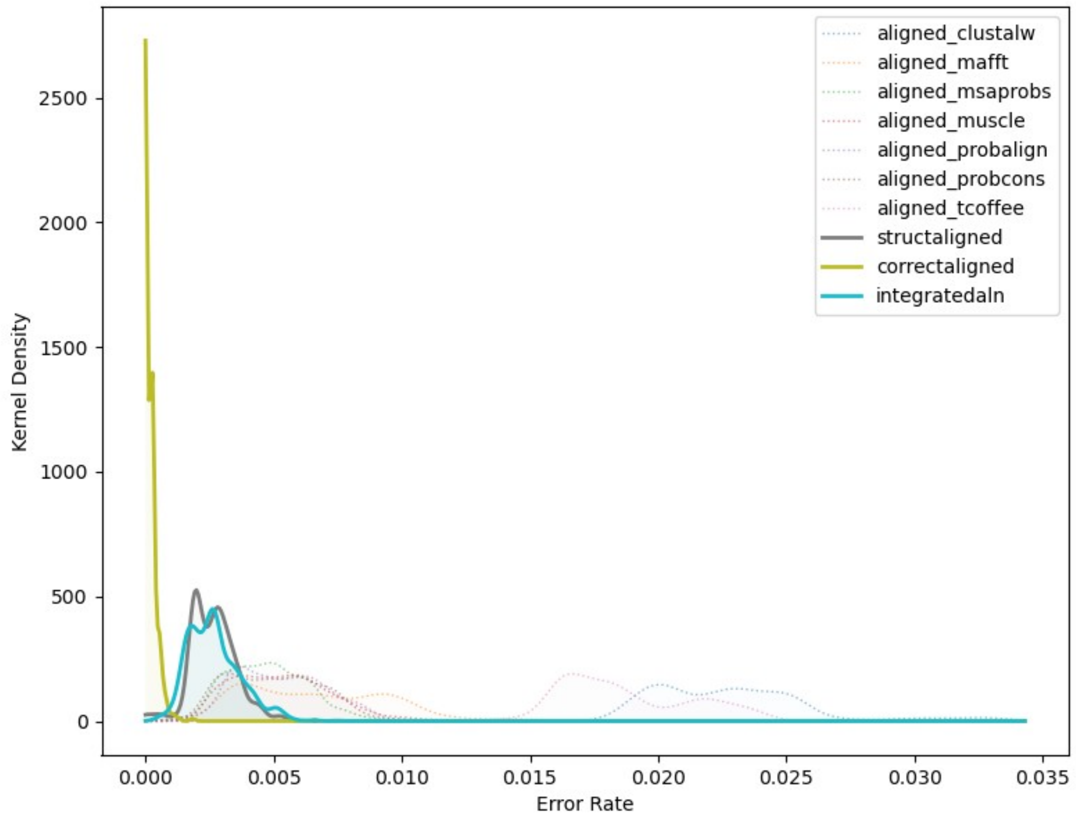

Figure S6 Part 5, RD Domain.

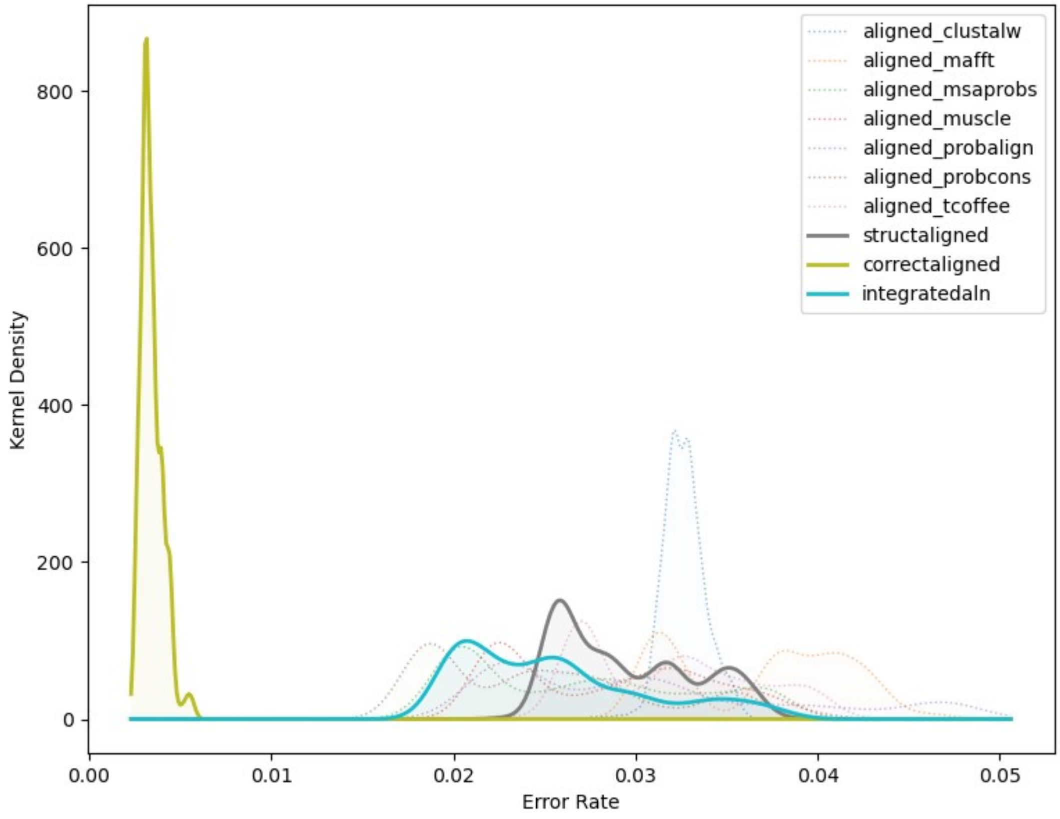

Figure S7 Part 1, CARD Domain. Ancestral sequence reconstruction (ASR) deletion error rates are low when the correct alignment is known in advance, higher when the alignment is inferred using sequence-alignment methods, and intermediate when structure-guided alignments or alignment-integrated ASR is used. We simulated extant and ancestral sequences for 5 protein domain families, using empirically-derived conditions, and aligned the resulting extant sequence data using structure-guided and 7 different sequence-alignment methods (see Methods). We used each alignment to infer the most likely ancestral sequence at each node on the phylogeny. Additionally, alignment-integrated ancestral sequences were generated by combining inferences from the 7 sequence-alignment methods (see Methods). In each case, we compared the inferred ancestral sequences to the correct simulated sequence to estimate ancestral sequence reconstruction (ASR) error rates (see Methods). ASR errors were divided into 4 “errorType” categories: 1) residue errors, in which both correct and inferred ancestral sequences have a residue at a given position in the alignment, but the residues differ; 2) insertion errors, in which the inferred sequence has a residue at a given alignment position, but the correct sequence has a gap state; 3) deletion errors, in which the inferred sequence has a gap, but the correct sequence has a residue, and 4) total errors, which combine residue- insertion- and deletion-errors. Sequence-wide error rates (errors/site) were computed by dividing the number of errors by the length of the pairwise alignment of the inferred and correct ancestral sequences. Error rate distributions were inferred by kernel density estimation. Here we report the ASR-error distributions for deletion errors. Part 1 shows results for the CARD domain family; results for other domains are shown in parts 2-5 below.

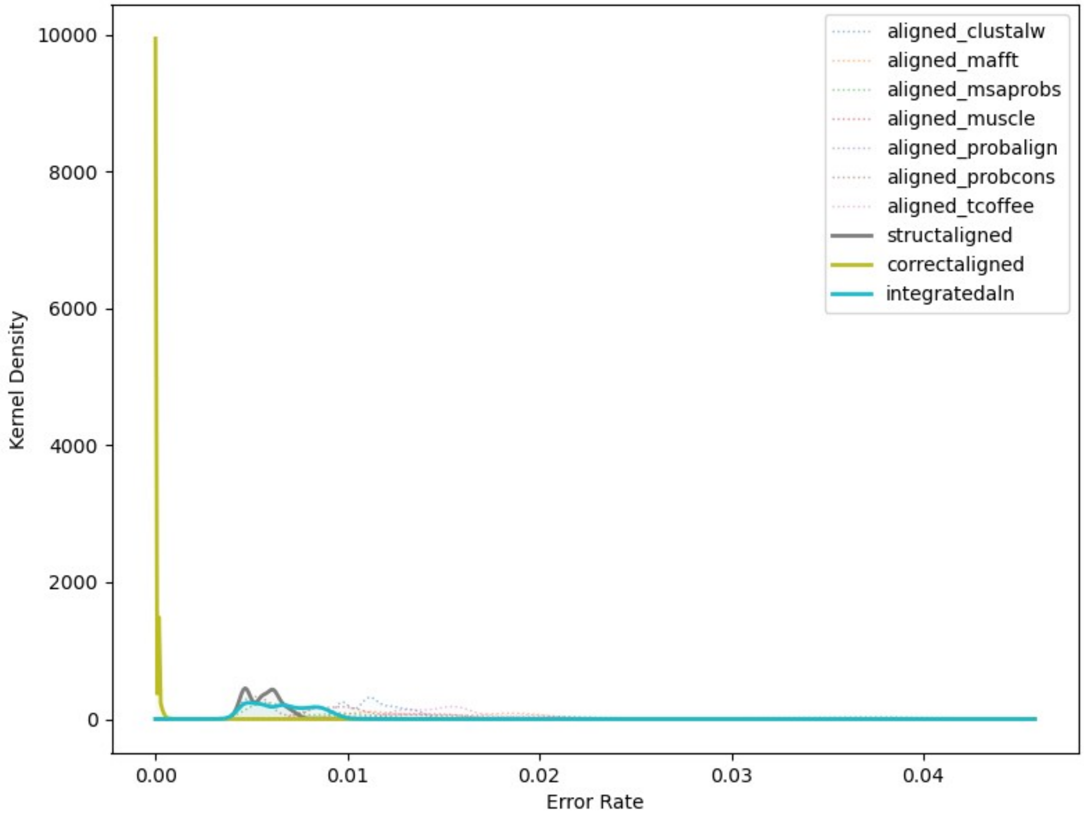

Figure S7 Part 2, DSRM1 Domain.

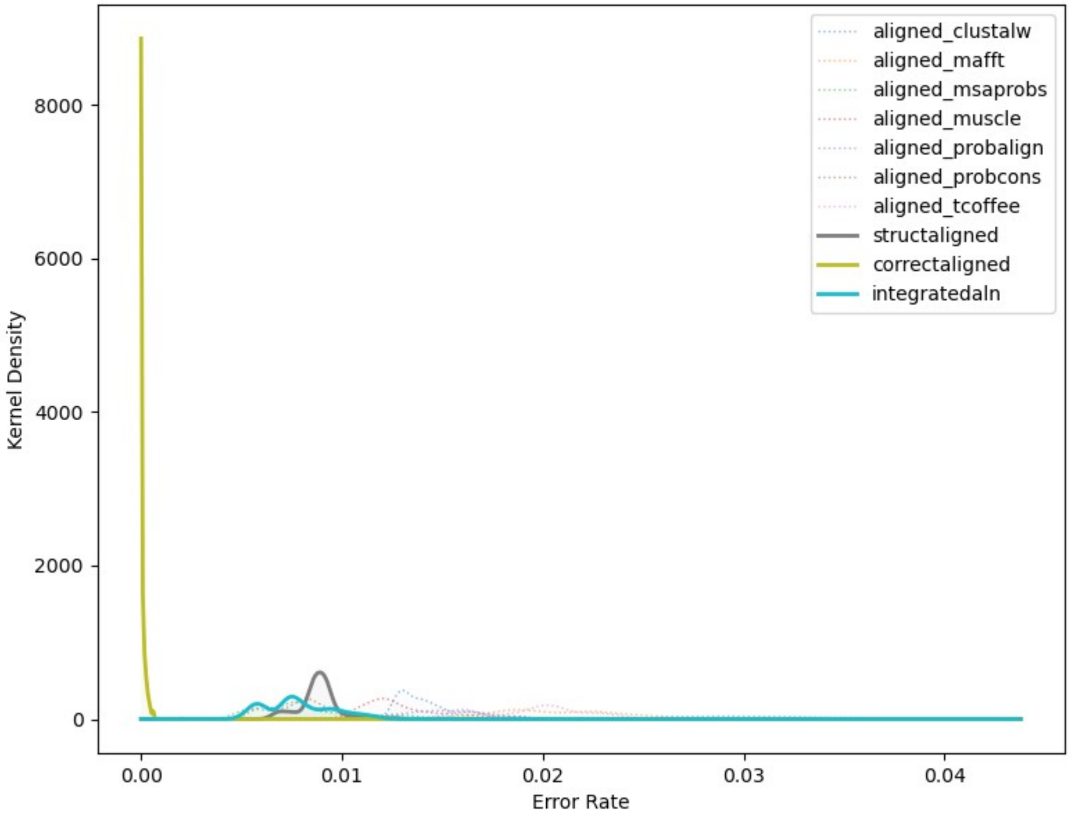

Figure S7 Part 3, DSRM2 Domain.

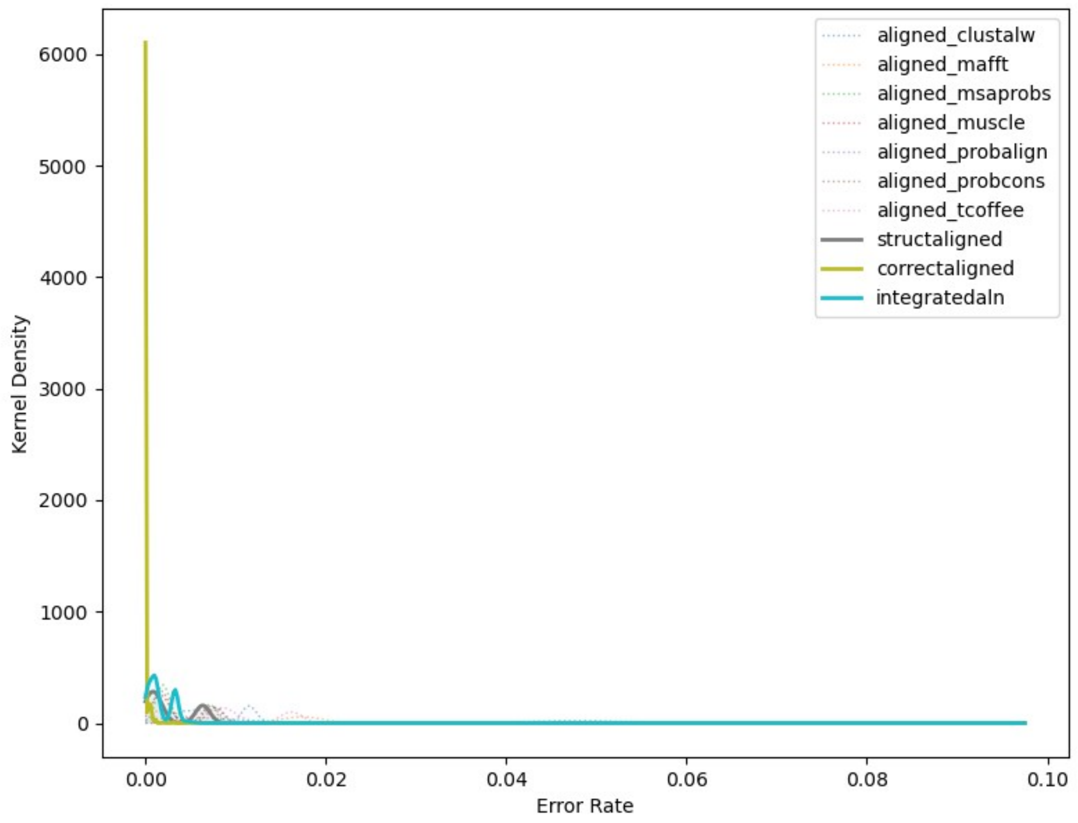

Figure S7 Part 4, DSRM3 Domain.

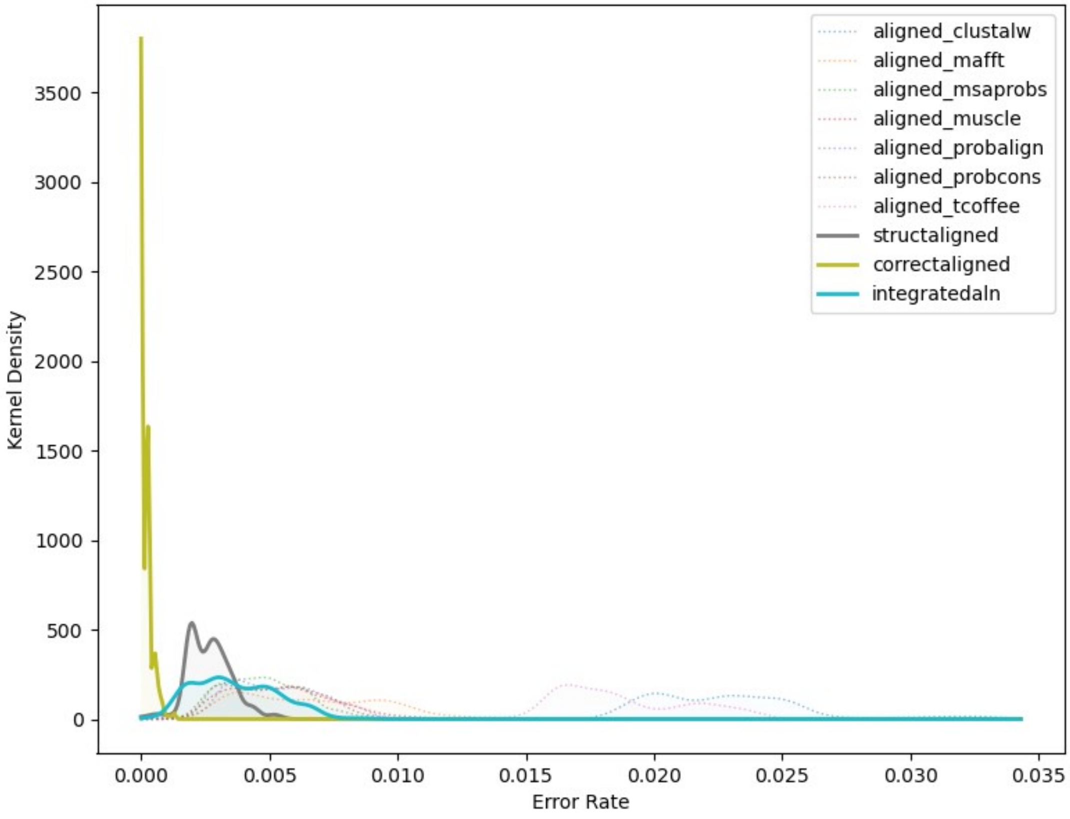

Figure S7 Part 5, RD Domain.

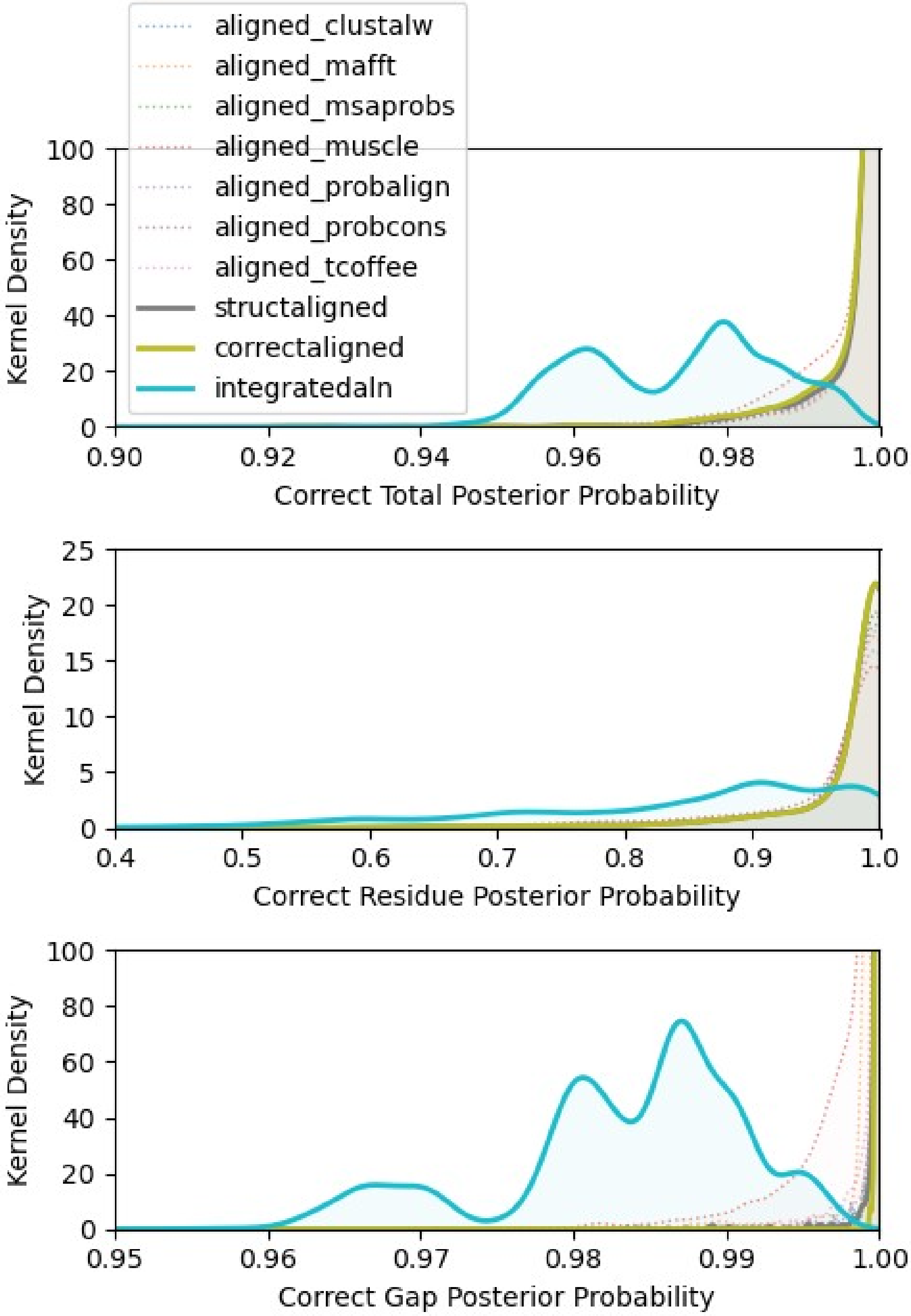

Figure S8. Support for the correct ancestral state is reduced by alignment-integrated ancestral sequence reconstruction when the correct state is inferred by maximum-likelihood. We simulated extant and ancestral sequences for 5 protein domain families, using empirically-derived conditions, and aligned the resulting extant sequence data using structure-guided and 7 different sequence-alignment methods (see Methods). We used each alignment to infer the most likely ancestral sequence at each node on the phylogeny. Additionally, alignment-integrated ancestral sequences were generated by combining inferences from the 7 sequence-alignment methods (see Methods). In each case, we compared the inferred ancestral sequences to the correct simulated sequence to identify ancestral sequence reconstruction (ASR) errors (see Methods). When the correct ancestral state was inferred as the maximum-likelihood state, we calculated the posterior probability (PP) of the correct state using an

empirical-Bayesian approach (see Methods). The distribution of posterior probabilities for the correct ancestral state, when no ASR error was made, was inferred by kernel density estimation. We report kernel density distributions for correctly-inferred ancestral gap states, residues and total (gaps + residues).

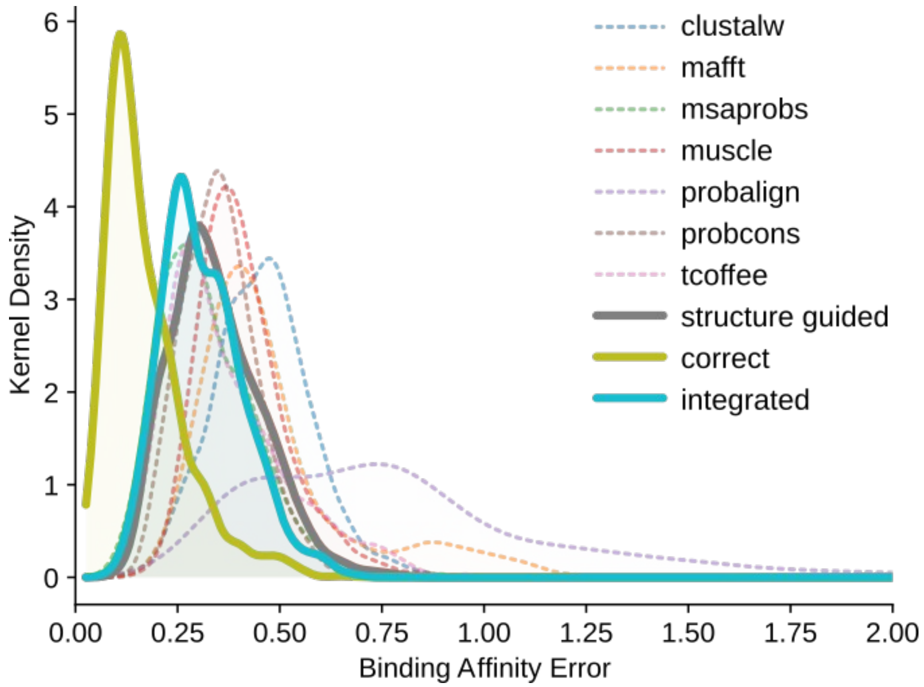

Figure S9. Alignment-integrated and structure-guided approaches produce less error in inferred structural and functional properties of ancestral proteins than single sequence-alignment methods. We simulated replicate extant and ancestral sequences by evolving an RNA-binding protein domain along its empirically-determined phylogeny, using a structure-guided alignment to determine the amino-acid composition and pattern of insertions/deletions. Ancestral sequences were inferred using structure-guided alignment, 7 different sequence-alignment methods and alignment-integration. We modeled the structure of each ancestral sequence and estimated its dsRNA binding affinity (pKd) using a computational approach. Errors in binding affinity were calculated by comparing values estimated from the correct ancestral sequences to those estimated using each alignment method. We used kernel density estimation to calculate the frequency distribution of binding-affinity errors.

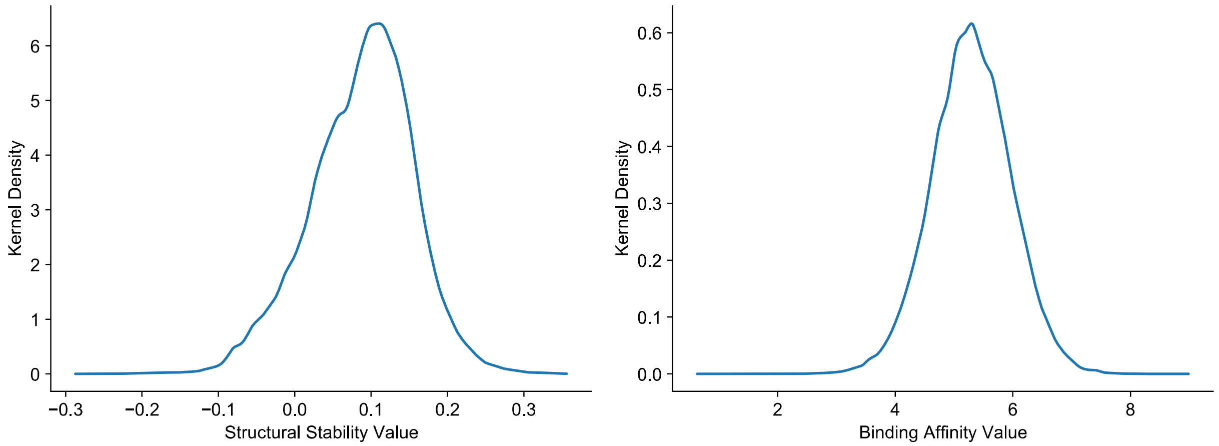

property	meanValue	medianValue	highestMode	StdDev	SEmean	| max |
Structural Stability	8.74E-02	9.37E-02	1.10E-01	6.74E-02	1.82E-04	3.56E-01
Binding Affinity	5.29E+00	5.29E+00	5.29E+00	6.72E-01	1.93E-03	8.99E+00

Figure S10. Simulated ancestral DSRM1 protein domains generated a range of values for structural stability and dsRNA-binding affinity. We simulated replicate extant and ancestral sequence data sets by evolving the DSRM1 protein domain family along its empirically-determined phylogeny, using a structure-guided alignment of empirical DSRM1 sequences to determine the amino-acid composition and pattern of insertions/deletions (see Methods). Structural and functional properties were unconstrained during the simulations. We modeled the structure of each simulated ancestral sequence and estimated its structural stability (ΔG) and dsRNA-binding affinity (pKd) using computational approaches (see Methods). The distributions of simulated structural stability (left) and binding affinity (right) values were inferred using kernel density estimation. Table inset reports the mean (and standard error), median, mode, standard deviation and absolute-maximum of each distribution.

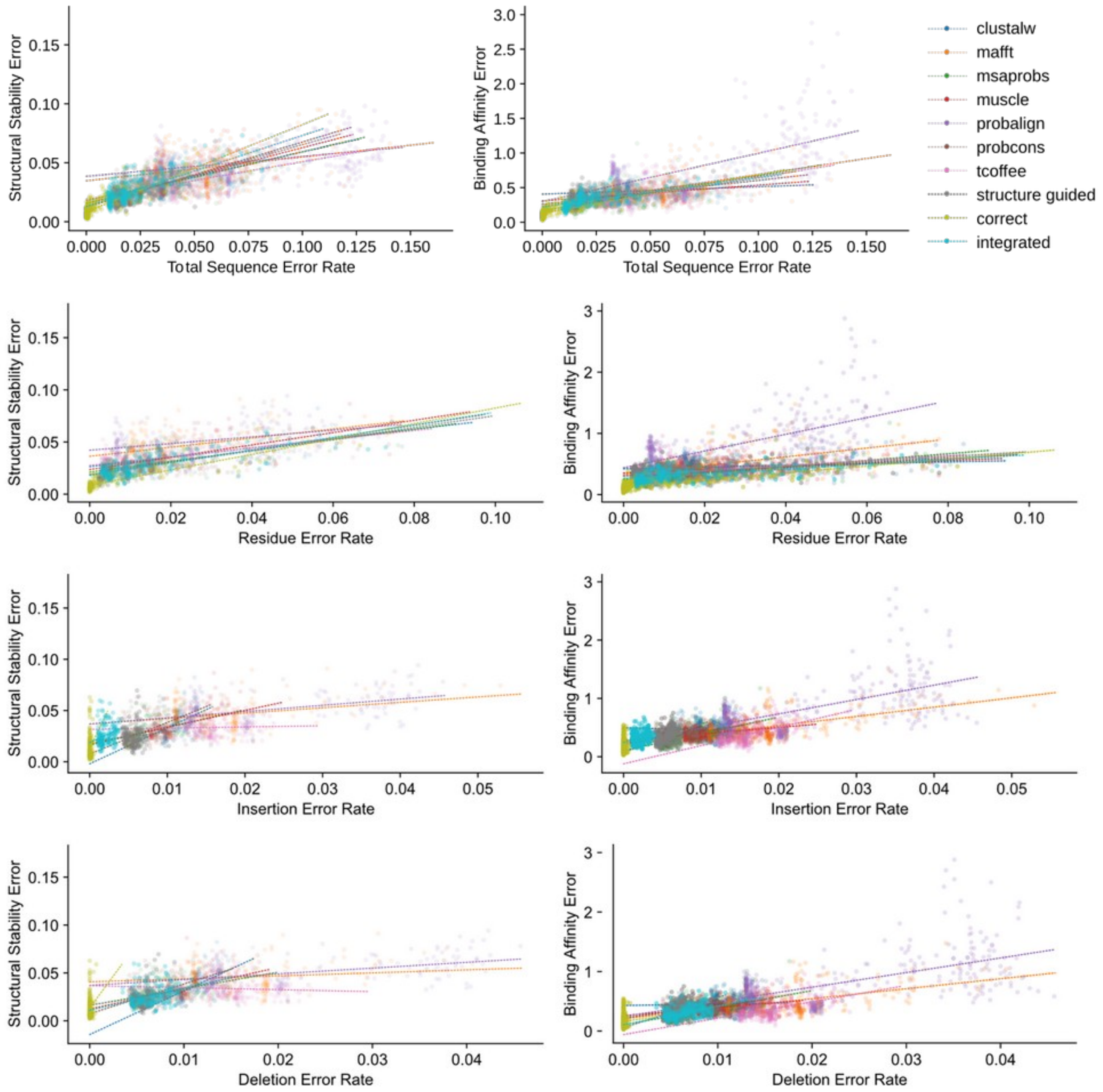

Figure S11. Errors in estimated structural stability and binding affinity of ancestral sequences are weakly correlated with errors in the inferred ancestral sequences. We simulated replicate extant and ancestral sequence data sets by evolving the DSRM1 protein domain family along its empirically-determined phylogeny, using a structure-guided alignment of empirical DSRM1 sequences to determine the amino-acid composition and pattern of insertions/ deletions (see Methods). Structural and functional properties were unconstrained during the simulations. We modeled the structure of each simulated ancestral sequence and estimated its structural stability (ΔG) and dsRNA-binding affinity (pKd) using computational approaches (see Methods). For each alignment method, we inferred the maximum-likelihood ancestral sequence at each node on the phylogeny, modeled the structure of the inferred ancestral sequence at each node, and estimated structural stability and binding affinity from the modeled structure. Structural stability and binding affinity errors were calculated as the absolute value of the difference between the value calculated from the correct simulated ancestral sequence and that

calculated from the inferred ancestral sequence. ASR errors were divided into 4 “errorType” categories: 1) residue errors, in which both correct and inferred ancestral sequences have a residue at a given position in the alignment, but the residues differ; 2) insertion errors, in which the inferred sequence has a residue at a given alignment position, but the correct sequence has a gap state; 3) deletion errors, in which the inferred sequence has a gap, but the correct sequence has a residue, and 4) total errors, which combine residue- insertion- and deletion-errors. Sequence-wide error rates (errors/site) were computed by dividing the number of errors by the length of the pairwise alignment of the inferred and correct ancestral sequences. For each type of ASR error and alignment method, we determined the best-fit linear relationship between ASR error rate and structural stability (left column) or binding affinity (right column) using ordinary least squares linear regression (dotted lines). Results for total ASR error rates are reported in the main text.

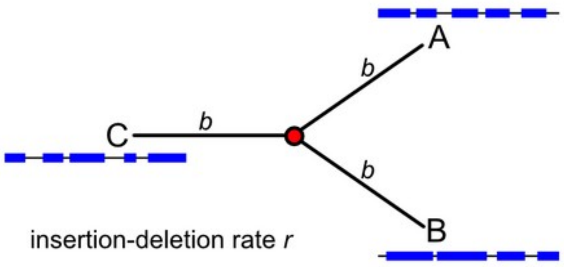

Figure S12. A minimal 3-taxon simulation provides a model system for investigating ancestral sequence reconstruction errors. We simulated protein sequences along a 3-taxon phylogeny with equal branch lengths (*b* substitutions/site) and the same insertion-deletion (indel) rate across the phylogeny (*r* indels/substitution/site). Red dot indicates the single ancestral node, and blue bars indicate possible aligned extant sequences generated by the simulation process.

While large-scale simulations are useful for evaluating the potential impact of alignment errors on ancestral sequence reconstruction studies in practice, it is difficult to systematically isolate the relevant variables necessary to determine why alignment errors generate ASR errors from such large-scale systems. Small model problems are needed to reliably infer causative mechanisms. In phylogenetic inference, the ‘4-taxon problem’ represents the smallest possible tree with multiple unrooted topologies, and studies of this simple ‘toy’ problem have led to major advancements in our understanding of the causes of phylogenetic inference errors and their potential solutions.

Here we propose a ‘3-taxon problem’ as the equivalent minimalist system for examining ancestral sequence reconstruction accuracy. For each node on the phylogeny, the most commonly-used marginal ASR algorithms use information from the 3 directly-connected nodes (typically 2 descendent nodes (eg, A,B) and one node representing the local ‘root’ (eg, C)) to infer the ancestral state probability distribution. The simple 3-taxon problem therefore allows us to learn potentially generalizable information about the factors impacting ASR accuracy while limiting the number of simulation variables to a small number.

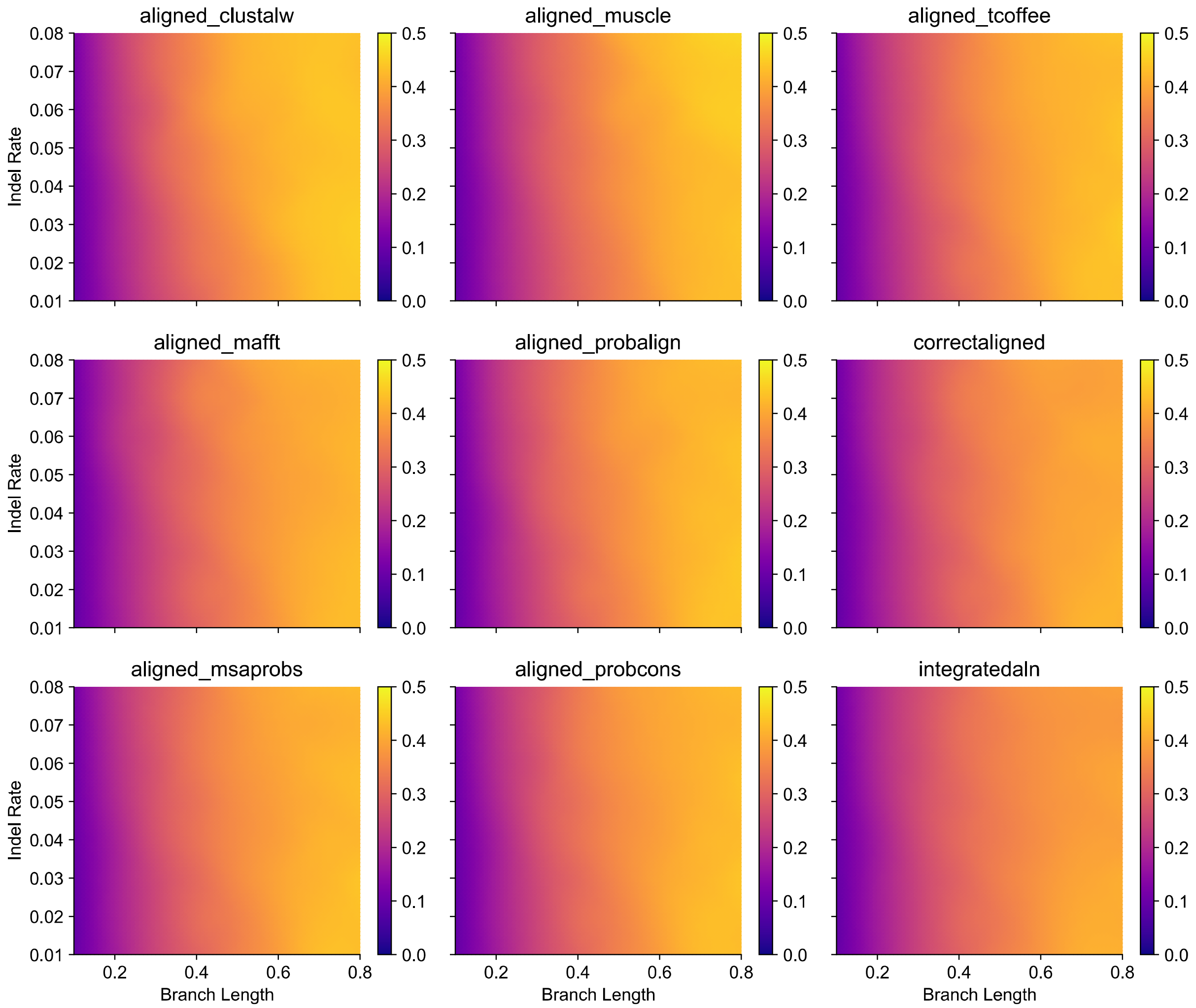

Figure S13. Alignment-integrated ancestral sequence reconstruction reduces total ASR error rates in 3-taxon simulations across a variety of conditions. We simulated protein sequences along a 3-taxon phylogeny with equal branch lengths and the same insertion-deletion (indel) rate across the phylogeny (see Methods, Supplementary Fig. S12). The resulting extant sequence data was aligned using 7 different sequence-alignment methods (see Methods). We used each alignment to infer the most likely ancestral sequence at the single node on the 3-taxon phylogeny. Additionally, alignment-integrated ancestral sequences were generated by combining inferences from the 7 sequence-alignment methods (see Methods). In each case, we compared the inferred ancestral sequence to the correct simulated sequence to estimate ancestral sequence reconstruction (ASR) error rates (see Methods). ASR errors were divided into 4 categories: 1) residue errors, in which both correct and inferred ancestral sequences have a residue at a given position in the alignment, but the residues differ; 2) insertion errors, in which the inferred sequence has a residue at a given alignment position, but the correct sequence has a gap state; 3) deletion errors, in which the inferred sequence has a gap, but the correct sequence has a residue, and 4) total errors, which combine residue-insertion- and deletion-errors. Sequence-wide error rates (errors/site) were computed by dividing the number of errors by the length of the pairwise alignment of the inferred and correct ancestral sequences. For each combination of branch-length and indel-rate, we plot the average

rate of total ASR errors over 100 replicate data sets for each alignment method, with purple indicating low ASR error rates, and yellow indicating high rates of ASR errors.

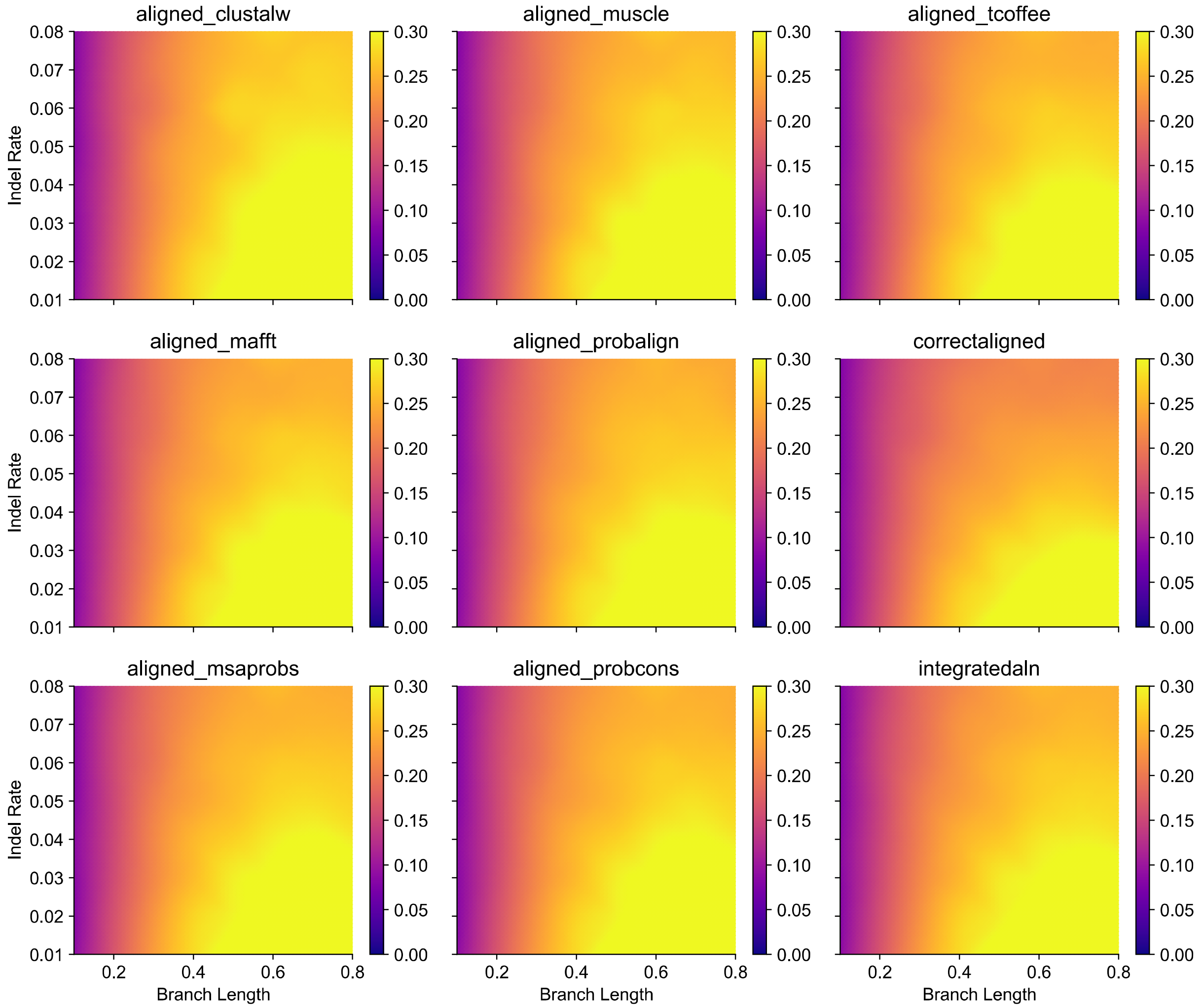

Figure S14. Alignment-integrated ancestral sequence reconstruction reduces residue ASR error rates in 3-taxon simulations across a variety of conditions. We simulated protein sequences along a 3-taxon phylogeny with equal branch lengths and the same insertion-deletion (indel) rate across the phylogeny (see Methods, Supplementary Fig. S12). The resulting extant sequence data was aligned using 7 different sequence-alignment methods (see Methods). We used each alignment to infer the most likely ancestral sequence at the single node on the 3-taxon phylogeny. Additionally, alignment-integrated ancestral sequences were generated by combining inferences from the 7 sequence-alignment methods (see Methods). In each case, we compared the inferred ancestral sequence to the correct simulated sequence to estimate ancestral sequence reconstruction (ASR) error rates (see Methods). ASR errors were divided into 4 categories: 1) residue errors, in which both correct and inferred ancestral sequences have a residue at a given position in the alignment, but the residues differ; 2) insertion errors, in which the inferred sequence has a residue at a given alignment position, but the correct sequence has a gap state; 3) deletion errors, in which the inferred sequence has a gap, but the correct sequence has a residue, and 4) total errors, which combine residue-

insertion- and deletion-errors. Sequence-wide error rates (errors/site) were computed by dividing the number of errors by the length of the pairwise alignment of the inferred and correct ancestral sequences. For each combination of branch-length and indel-rate, we plot the average rate of residue ASR errors over 100 replicate data sets for each alignment method, with purple indicating low ASR error rates, and yellow indicating high rates of ASR errors.

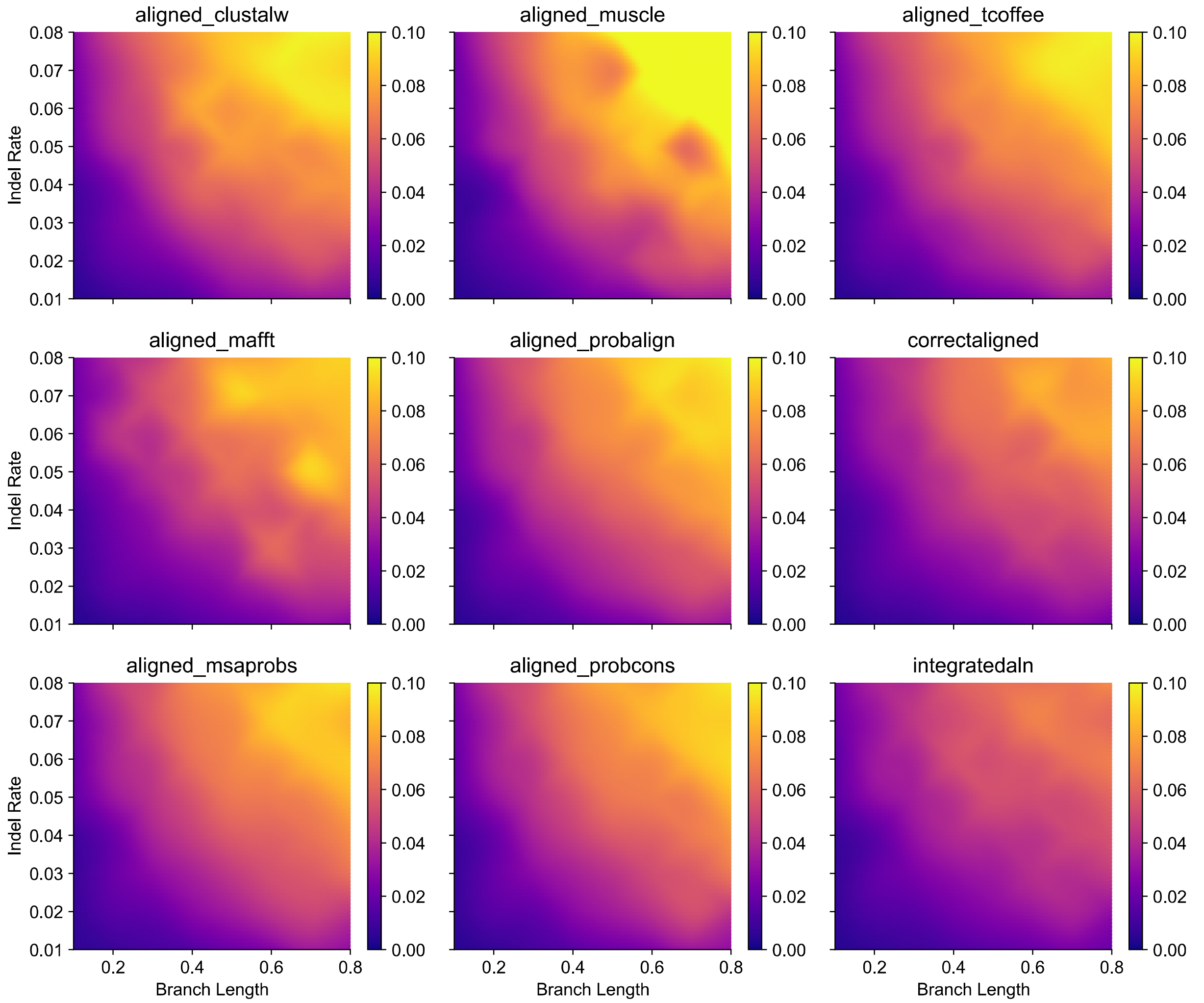

Figure S15. Alignment-integrated ancestral sequence reconstruction reduces insertion ASR error rates in 3-taxon simulations across a variety of conditions. We simulated protein sequences along a 3-taxon phylogeny with equal branch lengths and the same insertion-deletion (indel) rate across the phylogeny (see Methods, Supplementary Fig. S12). The resulting extant sequence data was aligned using 7 different sequence-alignment methods (see Methods). We used each alignment to infer the most likely ancestral sequence at the single node on the 3-taxon phylogeny. Additionally, alignment-integrated ancestral sequences were generated by combining inferences from the 7 sequence-alignment methods (see Methods). In each case, we compared the inferred ancestral sequence to the correct simulated sequence to estimate ancestral sequence reconstruction (ASR) error rates (see Methods). ASR errors were divided into 4 categories: 1) residue errors, in which both correct and inferred ancestral

sequences have a residue at a given position in the alignment, but the residues differ; 2) insertion errors, in which the inferred sequence has a residue at a given alignment position, but the correct sequence has a gap state; 3) deletion errors, in which the inferred sequence has a gap, but the correct sequence has a residue, and 4) total errors, which combine residue- insertion- and deletion-errors. Sequence-wide error rates (errors/site) were computed by dividing the number of errors by the length of the pairwise alignment of the inferred and correct ancestral sequences. For each combination of branch-length and indel-rate, we plot the average rate of insertion ASR errors over 100 replicate data sets for each alignment method, with purple indicating low ASR error rates, and yellow indicating high rates of ASR errors.

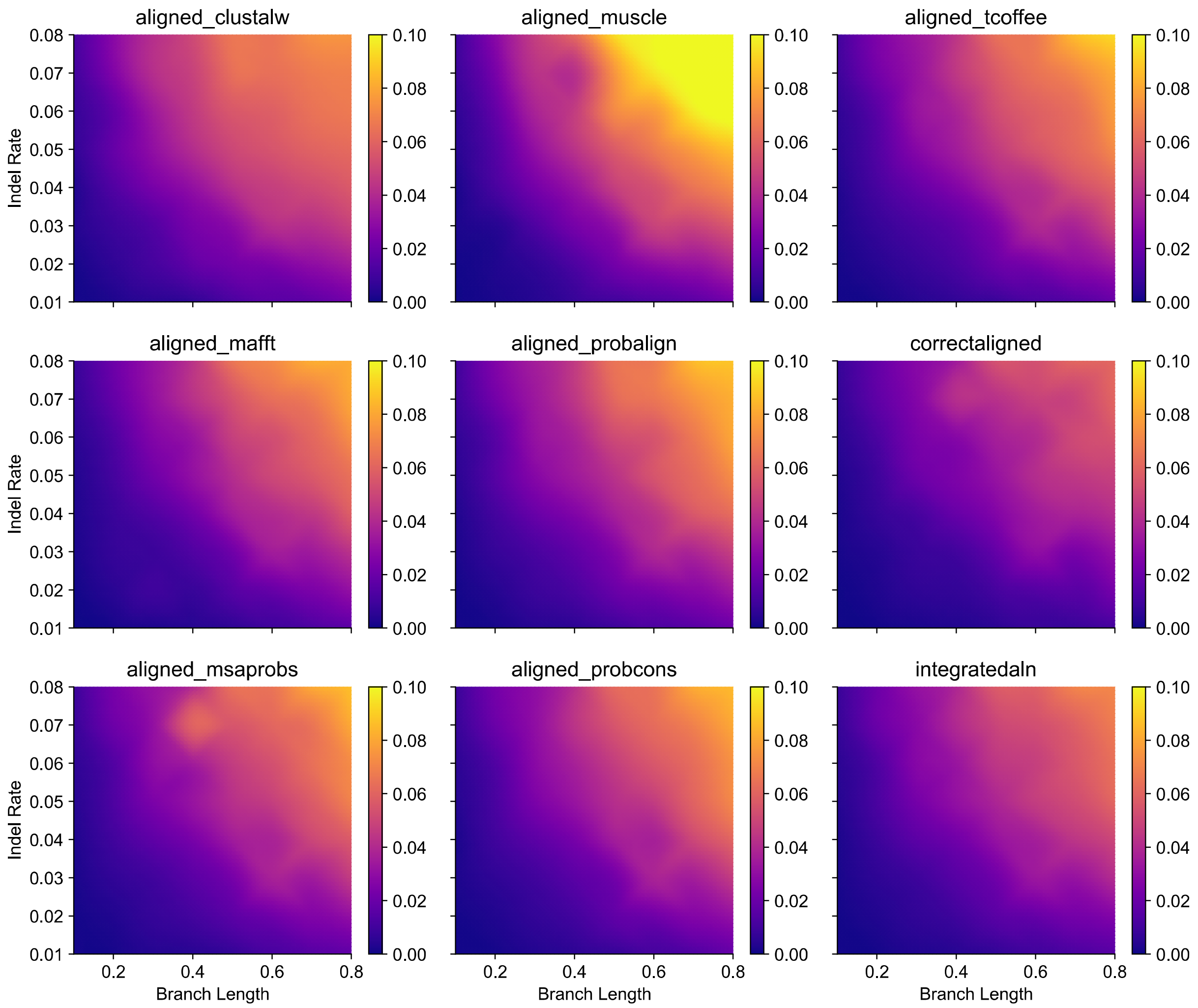

Figure S16. Alignment-integrated ancestral sequence reconstruction reduces deletion ASR error rates in 3-taxon simulations across a variety of conditions. We simulated protein sequences along a 3-taxon phylogeny with equal branch lengths and the same insertion-deletion (indel) rate across the phylogeny (see Methods, Supplementary Fig. S12). The resulting extant sequence data was aligned using 7 different sequence-alignment methods (see Methods). We used each alignment to infer the most likely ancestral sequence at the single node on the 3-taxon phylogeny. Additionally, alignment-integrated ancestral sequences were generated by combining inferences from the 7 sequence-alignment methods (see Methods). In

each case, we compared the inferred ancestral sequence to the correct simulated sequence to estimate ancestral sequence reconstruction (ASR) error rates (see Methods). ASR errors were divided into 4 categories: 1) residue errors, in which both correct and inferred ancestral sequences have a residue at a given position in the alignment, but the residues differ; 2) insertion errors, in which the inferred sequence has a residue at a given alignment position, but the correct sequence has a gap state; 3) deletion errors, in which the inferred sequence has a gap, but the correct sequence has a residue, and 4) total errors, which combine residue- insertion- and deletion-errors. Sequence-wide error rates (errors/site) were computed by dividing the number of errors by the length of the pairwise alignment of the inferred and correct ancestral sequences. For each combination of branch-length and indel-rate, we plot the average rate of deletion ASR errors over 100 replicate data sets for each alignment method, with purple indicating low ASR error rates, and yellow indicating high rates of ASR errors.
